# Twenty-Five Years of Research and Monitoring Using PUF Disk Passive Air Samplers

**DOI:** 10.1021/acs.est.5c17602

**Published:** 2026-04-16

**Authors:** Anita Eng, Yu-Mei Hsu, Tom Harner, Jacob Mastin, Samantha Wheadon

**Affiliations:** Air Quality Processes Research Section, 6347 Environment and Climate Change Canada, Toronto, Ontario M3H 5T4, Canada

**Keywords:** passive air sampler, polyurethane foam, sorbent-impregnated PUF, passive dry deposition sampler, persistent organic pollutants, emerging chemicals

## Abstract

For the past quarter century, polyurethane foam (PUF) disk-based passive air samplers (PASs) have grown as a pivotal tool in research and monitoring of persistent organic pollutants and emerging chemicals in ambient air in both the gas phase and on ambient particulate matter. Their low cost and ease of use have facilitated deployment at the regional and global scales. Modifications to the PUF–PAS have expanded its use for more specific purposes, such as the sorbent-impregnated PUF–PAS (SIP-PAS), which improves sorptive capacity for more volatile chemicals, and the passive dry deposition (PAS-DD) sampler, which captures larger particles and enables estimation of gas and particle deposition. This review summarizes studies characterizing uptake rates and partition coefficients of PUF disks for a wide range of compounds and evaluations of sampler design and performance. It synthesizes applications of PUF–PAS, SIP-PAS, and PAS-DD from 2000 to 2024 across diverse topics: ambient air measurements, indoor air quality, source emissions, health, and, most recently, biodiversity. Approximately 650 publications employing PUF disk-based PASs are summarized herein, demonstrating their increasing use and diversification. On the horizon, we envisage that the PUF–PAS will continue to transform and integrate fields of science and inform policy.

## Graphical abstract

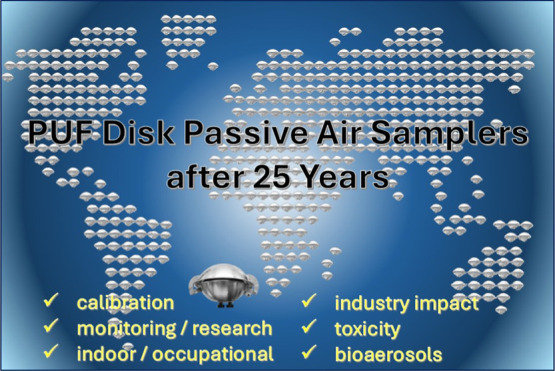

## 1 Introduction

Polyurethane foam (PUF) was first identified as an effective sorbent for sampling semivolatile organic compounds (SVOCs) in air by “Terry and Charlie” in 1973.^1^ Building on this finding, they later developed an active air sampler that incorporated a glass fiber filter (GFF)–PUF cartridge.^2,3^ Shoeib and Harner^4^ later evaluated PUF disks in 2000, demonstrating their applicability as passive air samplers for persistent organic pollutants (POPs).

Over the past ∼25 years, PUF disk passive air samplers (PUF–PAS) (Figure 1) have been extensively applied as a cost-effective and practical approach to quantify atmospheric concentrations of SVOCs, including POPs and emerging chemicals, in both the gaseous and particle-associated phases.^5,6^ The sampler operates on the principle of diffusive mass transfer driven by concentration gradients, enabling the accumulation of analytes without the need for electricity or active airflow. This fundamental simplicity allows for deployment at remote and infrastructure-limited locations and supports extensive spatial coverage in regional, continental, and global monitoring networks.

**Figure 1:**
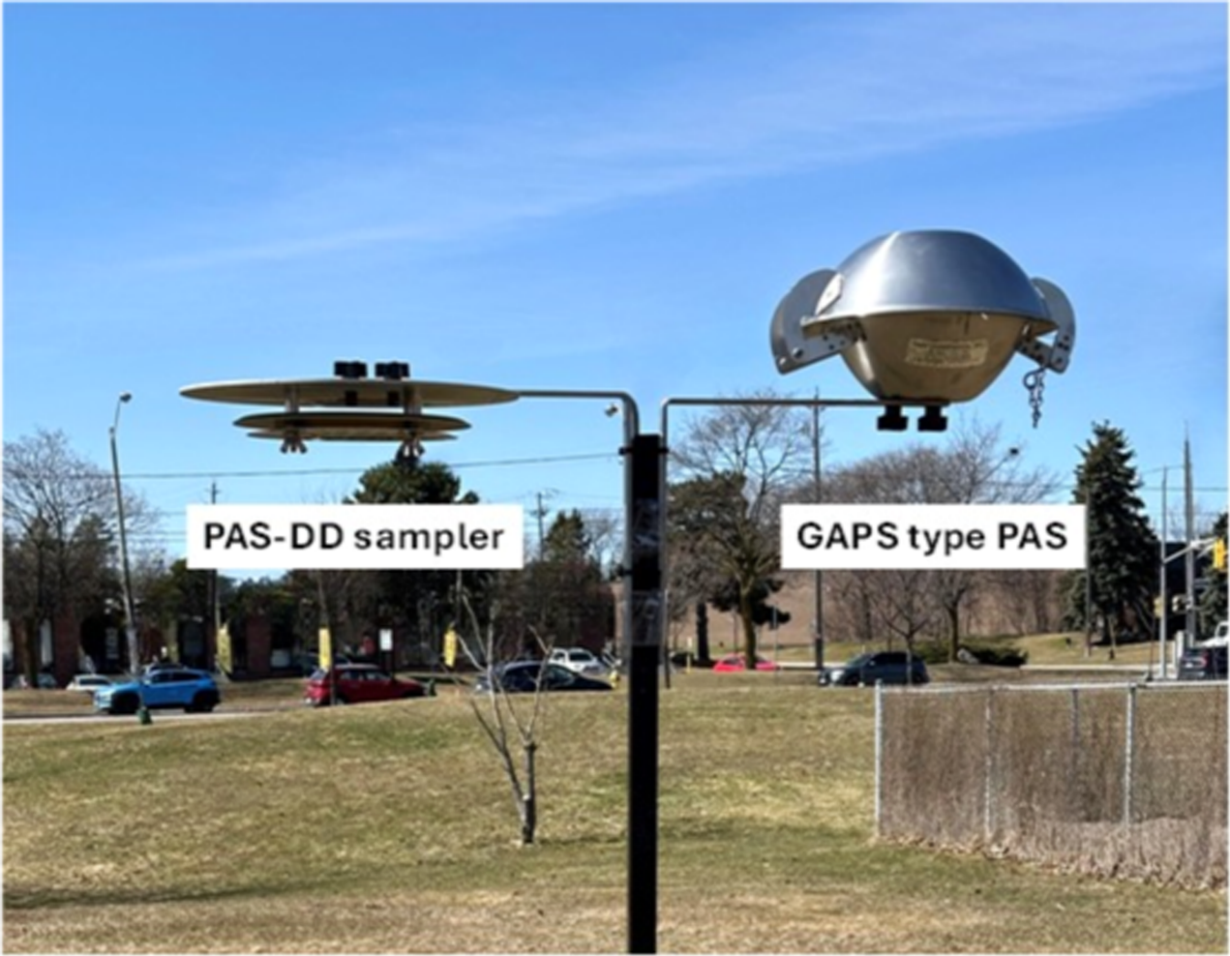
Passive dry deposition (PAS-DD) sampler and Global Atmospheric Passive Sampling (GAPS) network type passive air sampler (i.e., PUF–PAS or SIP-PAS).

A key advantage of the PUF–PAS is its capacity to integrate contaminant levels over extended sampling durations, typically several weeks to months and, in some cases, up to one year, thereby providing time-weighted average concentrations that smooth short-term variability. This temporal integration enhances the representativeness of the data for evaluating long-term atmospheric trends, source–receptor relationships, and interannual changes in pollutant transport dynamics.

The low cost, ease of deployment, and negligible maintenance requirements of PUF–PAS have facilitated their widespread use in both developed and developing regions, helping to close critical spatial data gaps in global air quality and chemical monitoring.^7–13^ Their affordability enables simultaneous deployment across large networks of sampling sites, supporting comparative assessments of pollutant gradients and long-range atmospheric transport. Data derived from such coordinated networks have informed national and international chemical management programs by documenting spatial and temporal patterns of legacy and emerging pollutants^14–18^ while also identifying emerging challenges associated with the occurrence and transformation of emerging chemicals in the atmosphere.^19^

Performance evaluation and intercomparison studies conducted by multiple research institutions have collectively demonstrated the robustness and reproducibility of the PUF–PAS method under diverse environmental conditions.^20–23^ To broaden its chemical capture capability, the original PUF disk was further modified into a sorbent-impregnated PUF (SIP),^24^ which enhances the collection of more volatile compounds. The PUF-based PAS configurations have been recognized and promoted by the United Nations Environment Programme (UNEP) as essential and pragmatic tools to address the growing global demand for cost-effective monitoring of POPs and other emerging environmental contaminants in air.^25^ Their success derives from their low cost, operational simplicity, and demonstrated capability to effectively capture both gas- and particle-associated SVOCs. These attributes have enabled unique spatial assessments of chemical distributions in air and have fostered innovative, cross-disciplinary applications that bridge scientific research and policy.^26^

This review details the technical evolution of the PUF-based PAS and provides a comprehensive overview of the diverse applications of the PUF-based PAS from 2000 to 2024, including evaluations of sampler performance across diverse chemical classes, research, and monitoring of POPs and emerging chemicals in (1) ambient air, (2) indoor and occupational exposure studies, (3) investigations of emission sources, and (4) health studies assessing inhalation exposure. Future directions and novel applications of the PUF-based PAS are suggested to guide methodological development and broaden their role in air monitoring and chemical management.

## 2 Evolution of PUF–PAS

### 2.1 PUF Passive Air Sampler (PUF–PAS)

The original PUF–PAS housing design (2000) is shown in Figure 2a. In this configuration, the PUF disk was mounted on a central rod, secured by a washer on one side for SVOCs. To replace the PUF disk, operators had to completely disassemble the housing, making field maintenance cumbersome and time-consuming. In approximately 2007, the sampler housing was redesigned to improve usability and performance under harsh environmental conditions (e.g., temperatures as low as −40 °C), as shown in Figure 2b (model: TE-200-PAS, Tisch Environment). The new hinged stainless-steel dome design allowed for quick and convenient replacement of PUF disks, eliminating the need to fully disassemble the housing, as required in the original configuration. This modification significantly reduced the time and effort needed for sample changes in the field and greatly improved the reliability during extreme weather. The hinged housing, now commercially available, represents a durable and user-friendly upgrade that enhances both operational efficiency and long-term sampler performance.

**Figure 2:**
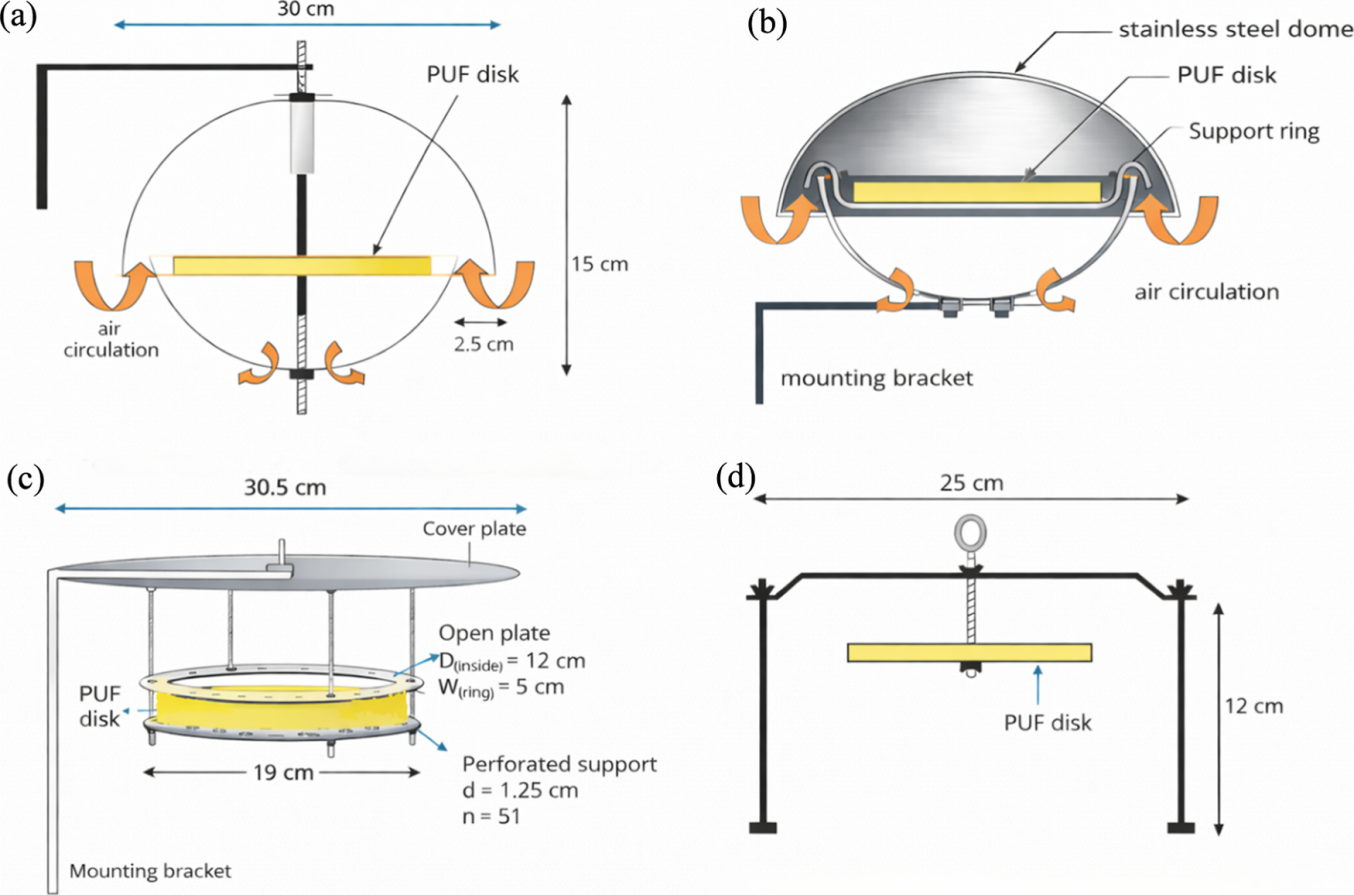
Schematic of (a) original double-dome passive air sampler developed by Shoeib and Harner,^5^ (b) double-dome passive air sampler modified in ∼2007, (c) passive dry deposition sampler, and (d) indoor passive air sampler with a facedown opening developed by Wilford et al.^36^

### 2.2 Sorbent-Impregnated PUF Passive Air Sampler (SIP-PAS)

The PUF–PAS was modified to enhance its sorptive capacity and improve retention of more volatile compounds, including fluorotelomer alcohols (FTOHs) and volatile POPs (e.g., perfluoroalkyl and polyfluoroalkyl substances (PFAS)). This modification involves impregnating the PUF substrate with a high surface area styrene–divinylbenzene copolymer (ground XAD-4 resin powder), producing a sorbent-impregnated PUF (SIP) disk, as first demonstrated by Shoeib et al.^24^ The incorporation of ground XAD-4 significantly increases the overall sorptive surface area and affinity for volatile species, thereby expanding the applicability of passive air sampling to chemicals with higher vapor pressures and lower octanol–air partition coefficients (*K*_OA_).

### 2.3 Passive Dry Deposition Sampler (PAS-DD)

The PUF–PAS has also been adapted into a passive dry deposition sampler (PAS-DD, model: TE-PAS-DD, Tisch Environment), which can be used to estimate atmospheric loadings (i.e., dry deposition fluxes) (Figure 2c).^27^ The PAS-DD is constructed from stainless steel and consists of a large circular cover plate positioned in parallel with a PUF disk, which is mounted 2 cm below on a perforated support plate (Figure 1). The PAS-DD functions by intercepting atmospheric contaminants deposited via gravitational settling and wind-driven transport. The cover plate shields the PUF disk from wet deposition, debris, and direct sunlight while enabling the collection of gas- and particle-phase compounds through dry deposition. This design has been successfully applied for monitoring polycyclic aromatic compounds (PACs),^28–30^ fugitive dust,^31^ trace metals,^31–33^ and current-use pesticides (CUPs).^27,29,30,34,35^

### 2.4 Indoor Sampler and Open-Face Design

In addition to SIP-PAS and PAS-DD, Wilford et al.^36^ introduced modified housing for indoor applications featuring a more open configuration (Figure 2d) to improve air circulation around the PUF disk under low-ventilation conditions typical of indoor settings.

Several modifications to the PUF–PAS have been developed by subsequent researchers to improve its performance under specific environmental conditions and for targeted applications.^37,38^ For example, Tao et al.^39^ and Abdallah and Harrad^40^ modified the PUF–PAS by incorporating a GFF to enhance the collection of particle-bound polycyclic aromatic hydrocarbons (PAHs) in indoor air.

Beyond incorporating a GFF or altering the housing design, the PUF–PAS has been further adapted into a personal air wearable sampler (PAWS)^41^ and a miniature bird-borne passive air sampler^42–45^ for assessing human and gull exposure. These developments highlight the versatility of passive air sampling and illustrate its potential for continued methodological refinement to address emerging research needs. In this broader context, Wania and Shunthirasingham^46^ have comprehensively compared different types of passive air samplers, including PUF-based PAS, with respect to their theoretical basis and applications.

## 3 Characterization and Performance Studies of PUF–PAS, SIP-PAS, and PAS-DD

### 3.1 PUF–PAS Theoretical Foundation

The operating principle of the PUF–PAS is grounded in Fick’s first law of diffusion, which describes the transport of molecules as a flux driven by a concentration gradient. In a PUF–PAS, airborne chemicals diffuse from air into a PUF disk, where they are sorbed by the polymer matrix. This process occurs through a stagnant air boundary layer adjacent to the PUF surface and continues until equilibrium is approached between the gas phase and the sorbent phase. The uptake flux, *F*(pg/day)), can be expressed as eq 1:^4,20,22,47–51^

1 $$F=\frac{{dM}_{PUF}}{dt}=k_{O}\times A_{PUF}\times \left[C_{A}-\frac{C_{PUF}}{K_{PUF-A}}\right]$$

where *M*_PUF_ is the accumulated mass in the PUF disk (pg), *t* is the time (day), *k*_O_ (m/day) is the overall mass transfer coefficient, *A*_PUF_ is the planar surface area (*m* ^2^) of the PUF disk, *C*_A_ and *C*_PUF_ are the concentrations in air and in the PUF disk (pg/m^3^), respectively, and *K*_PUF‑A_ is the PUF–air partition coefficient (dimensionless). The overall mass transfer coefficient, *k*_O_, depends on resistances to diffusion in both the air-side and PUF-side boundary layers as Whitman’s two-film theory, eq 2

2 $$\frac{1}{k_{O}}=\frac{1}{k_{A}}+\frac{1}{k_{PUF}\times K_{PUF-A}}$$

where *k*_A_ and *k*_PUF_ are the mass transfer coefficients (m/day) for the air side and PUF side, respectively. For most SVOCs, the process is air-side controlled because molecular diffusion through the boundary layer in air represents the dominant resistance.^52^

The air concentration (*C*_A_) can therefore be calculated from the total accumulated mass and the effective sampling rate (*R*_s_) as eq 3

3 $$C_{A}=\frac{M_{PUF}}{R_{s}\times t}$$

where *R*_s_ = *k*_O_ × *A*_PUF_ is the sampling rate (m^3^/day).

The theory of PUF–PAS and SIP-PAS has been comprehensively described and discussed by Wania and Shunthirasingham,^46^ Salim and Górecki,^53^ and Qu et al.^54^

### 3.2 Sampling Rate and Influencing Factors

The sampling rate (*R*_s_) is a pivotal parameter for converting the accumulated mass on PUF to air concentrations. Its magnitude is influenced by three primary factors: (a) physical design parameters (PUF geometry and housing type); (c) environmental conditions (temperature, wind speed, and turbulence); and (b) compound properties (volatility and diffusivity).

For the Global Atmospheric Passive Sampling (GAPS) network-type PUF–PAS (PUF disk: 14 cm diameter × 1.35 cm thickness),^55^ the nominal sampling rate is ∼4 m^3^/d for both gas-phase and fine-particle-bound compounds.^4^ Slightly higher rates have been reported for the MOnitoring NETwork (MONET) configuration (15 cm × 1.5 cm PUF disk)^15^ as the *R*_s_ is a function of the surface area of the planar PUF disk (*A*_PUF_). Housing geometry exerts a dominant influence on the sampling performance. Open-face designs (Figure 2d) allow unobstructed air flow across the PUF surface, thereby promoting rapid air exchange and mass transfer, yielding higher sampling rates.^36^ In contrast, double-dome or other enclosed housings, typically employed for outdoor monitoring to shield samplers from precipitation and direct sunlight, introduce additional diffusion barriers and maintain more consistent sampling rates.

Tuduri et al.^52^ demonstrated that mass transfer between air and the PUF disk is primarily governed by gas-phase diffusion through this boundary layer, rather than by internal diffusion within the PUF itself. Consequently, the air-side resistance dominates the overall uptake kinetics. Wind speed therefore becomes a critical environmental variable: elevated wind speeds thin the boundary layer, enhancing mass transfer and increasing the apparent sampling rate, often exceeding ∼4 m^3^/day under well-ventilated outdoor conditions. Conversely, under calm or stagnant air, the boundary layer thickens, suppressing diffusion and lowering the sampling rate. For indoor applications, where air movement is typically limited, the use of an enclosed or double-dome housing further impedes mass transfer and leads to substantial under-sampling. In such environments, an open-face housing design is preferred, as it minimizes boundary-layer resistance and provides a more representative measure of indoor air concentrations.^38^

Temperature also indirectly affects *R*_s_ by affecting both *K*_PUF‑A_ and compound diffusivity. Elevated temperatures lower *K*_PUF‑A_ values (reducing sorption capacity), potentially accelerating the approach to equilibrium and limiting the duration of the linear uptake phase. The same principles apply to the SIP–PAS and PAS–DD systems. In all cases, sampler geometry, environmental conditions, and chemical-specific partitioning properties collectively determine performance, effective sampling volume, and comparability across sites and studies.

### 3.3 Uptake Regimes and Partitioning Behavior

The uptake of SVOCs by PUF–PAS is governed by a combination of diffusive transport, partitioning, and chemical properties, such as volatility, polarity, and particle affinity. The overall uptake follows three kinetic regimes (eq 1):^4^

1. Linear (kinetic control): *C*_PUF_ ≪ *K*_PUF‑A_ × *C*_A_; uptake increases linearly with time, *M*_PUF_ ≈ *R*_s_ × *C*_A_ × *t*. In this regime, analyte accumulation is controlled primarily by air-side diffusion, and the sorptive medium remains far from saturation. The *R*_s_ is the dominant parameter.
2. Curvilinear (transition): As uptake proceeds, the air–PUF concentration gradient decreases, and the net diffusion flux begins to decline. The internal concentration in the PUF becomes appreciable, and both diffusion and equilibrium partitioning jointly govern uptake kinetics. The rate at which equilibrium is approached becomes increasingly dependent on the compound-specific partition coefficient (*K*_PUF‑A_). Both *R*_s_ and *K*_PUF‑A_ are critical in this regime.
3. Equilibrium (partition control): *C*_PUF_ ≈ *K*_PUF‑A_ × *C*_A_. At equilibrium regime, no net mass transfer occurs between air and the sorbent, as the PUF is in equilibrium with the surrounding air. Uptake is fully controlled by the partition coefficient, and the accumulated mass remains constant over time. This regime typically applies to more volatile or weakly sorbing compounds where *K*_PUF‑A_ is the key parameter.

For most SVOCs with intermediate volatility (log *K*_OA_ ≈ 9–12, *K*_OA_: octanol-air partition coefficient), the linear regime dominates over typical deployment periods (4–12 weeks), permitting use of the *R*_s_ = 4 m^3^/d for calculating effective air volume (Figure 3). More volatile species (log *K*_OA_ < 8) will approach equilibrium within this time frame, necessitating compound-specific corrections for nonlinear uptake using measured or modeled *K*_PUF‑A_ and the SIP–air partition coefficients (*K*_SIP‑A_) for estimating the effective air volume. In contrast, highly involatile chemicals (log *K*_OA_ > 13) exhibit strong sorption to PUF and maintain a linear uptake phase that can extend for several years. In addition, these high *K*_OA_ compounds exist mainly associated with particulate matter in ambient air and calibration results indicate that sampling rates are comparable to those of gas-phase compounds.^57^

**Figure 3:**
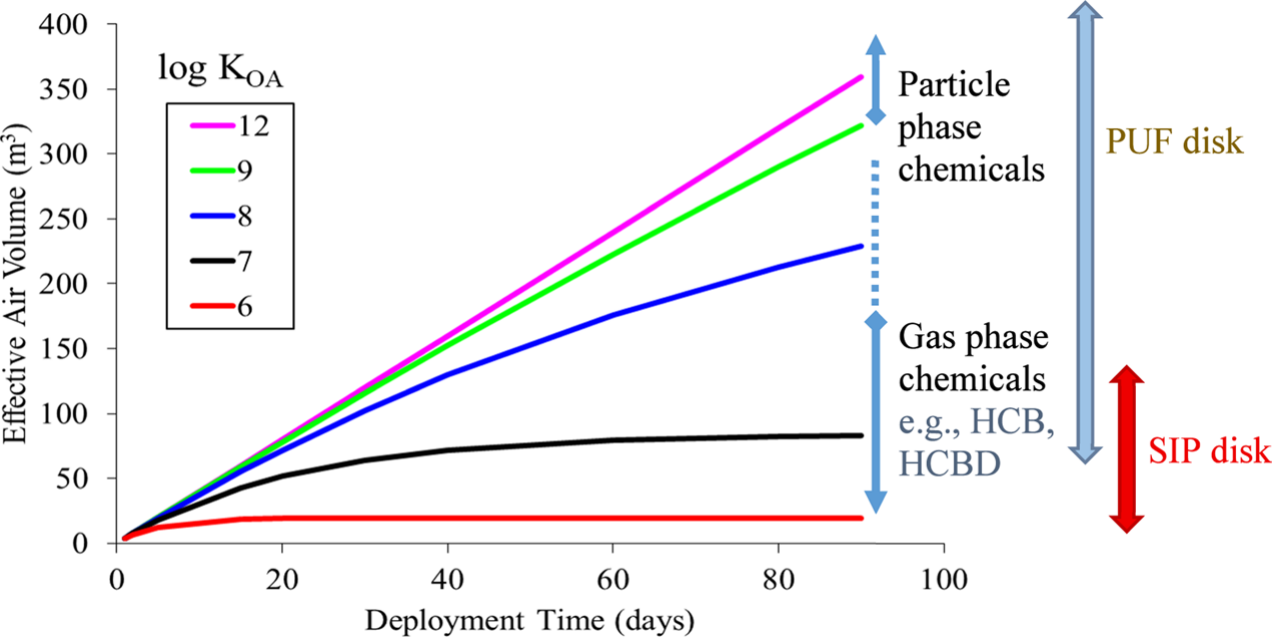
Uptake profile of the PUF disk sampler. The linear-phase sampling rate is ∼4 m^3^/day for GAPS-type samplers—for both gas-phase and particle-phase (up to 5 μm) chemicals. Arrows on the right indicate typical ranges of effective air volume for the application of PUF vs SIP disk samplers. The profile was constructed using the GAPS Network template,^56^ accounting for the approach to equilibrium for chemicals with low octanol-air partition coefficients (*K*_OA_) and low PUF-air partition coefficients (*K*_PUF‑A_).

### 3.4 Calibration and Modeling of the Sampling Rate and Partition Coefficients

To estimate ambient concentrations from PUF–PAS, numerous studies have been conducted to determine *R*_s_, *K*_PUF‑A_, and *K*_SIP‑A_. Table 1 summarizes the studies that have examined these parameters for a broad spectrum of SVOCs under various environmental conditions, including indoor,^58^ outdoor,^58^ chamber (or wind tunnel),^59–61^ and modeling studies.^22,62,63^ The SVOCs include CUPs,^64,65^ novel flame retardants (nFRs),^66^ organochlorine pesticides (OCPs),^67^ organophosphate ester (OPEs),^7^ PACs,^68^ polybrominated diphenyl ethers (PBDEs),^49,69^ polychlorinated biphenyls (PCBs),^70^ polychlorinated dibenzo-p-dioxins and polychlorinated dibenzofurans (PCDD/Fs),^71^ PFAS,^72^ and others.^73–76^ In practice, *R*_s_ values are typically established either through calibration against active air sampling (AAS) methods^77^ or by employing depuration compounds (DCs) preloaded into the PUF or SIP prior to deployment.^50,78^ The PUF–PAS has been applied to a broad spectrum of SVOCs with diverse chemical and physical properties. Accordingly, different DCs are selected to match the characteristics of the target analytes. Several DCs have been reported as effective for estimating *R*_s_, including isotopically labeled or unlabeled PCB congeners,^51,78–81^ deuterated γ-hexachlorocyclohexane (d_6_-γ-HCH),^22,79^ ^13^C_8_- perfluorooctanoic acid (PFOA),^82^ perfluoro-*n*-(1,2,3,4-^13^C_4_) octanoic acid (MPFOA),^83^ D_15_-triphenyl phosphate (TPhP),^84^ chlorpyrifos-methyl-*d*_6_ (CPFM-*d*_6_),^85^ azinphos ethyl-*d*_10_ (AZE-*d*_10_),^85^ ^13^C_6_—hexachlorobenzene (HCB),^81^ and ^13^C_12_-4,4′-dichloro-diphenyl-trichloroethane (DDT).^81^

**Table 1:** Studies Characterizing Sampling Rates (*R*_s_) and Partition Coefficients (*K*_PUF‑A_, *K*_SIP‑A_) for PUF–PAS, SIP-PAS, and PAS-DD

parameter^a^		location/site	setting^b^	reference	year	CUPs^c^	FRs	OCPs	PAH/PACs	PCBs	PCDD/Fs	PFAS	others
	PUF–PAS												
*R*_s_	K	Office building (Toronto, Canada)	I	Shoeib and Harner^4^	2002					×			PCNs
*R*_s_		Six locations along north–south transect (Chile)	O	Pozo et al.^7^	2004		×	×		×			
*R*_s_		Laboratories and offices (Ottawa, Canada)	I	Wilford et al.^36^	2004		×						
*R*_s_	K	IADN sites (Great Lakes Basin, Canada)	O	Gouin et al.^92^	2005		×	×		×			
*R*_s_		Southwest of Regina (Saskatchewan, Canada)	O	Waite et al.^93^	2005	×		×					
*R*_s_		Vacant offices (Birmingham, UK)	I	Hazrati and Harrad^94^	2007		×			×			
	K	sorption study for up to 100 organic vapors across 15–95 °C, varying humidities, and multiple PUF types	C	Kamprad and Goss^95^	2007								
*R*_s_		Residential areas (Luzon Island, Philippines)	O	Santiago and Cayetano^96^	2007				×				
*R*_s_		Universidad Nacional station (Costa Rica)	O	Gouin et al.^97^	2008	×							
*R*_s_		MSWI, power plant, background (Spain)	O	Mari et al.^73^	2008					×	×		PCNs
*R*_s_		Hazelrigg field station (UK)	O	Chemefa et al.^49^	2009		×			×			
*R*_s_		Hazelrigg field station (UK)	O	Chemefa et al.^69^	2009		×						
*R*_s_		Norunda forest site (Uppsala, Sweden)	O	Moeckel et al.^78^	2009					×			
*R*_s_		University of Iowa (USA)	I, O	Persoon and Hornbuckle^50^	2009					×			
*R*_s_		GAPS Network (Global)	O	Pozo et al.^98^	2009		×	×		×			
*R*_s_		Alloy factory (Sweden)	I	Bohlin et al.^99^	2010				×				
*R*_s_		National University of Singapore (Singapore)	O	He and Balasubramanian^81^	2010			×	×				
*R*_s_		Urban background (Birmingham UK)	O	Desborough and Harrad^100^	2011					×			
*R*_s_		Wind tunnel and computational method	C, M	May et al.^59^	2011					×			
*R*_s_		University of Toronto (Canada)	O	Melymuk et al.^20^	2011		×		×	×			PCMs
*R*_s_	K	Urban and rural residential (Philippines)	O	Santiago and Cayetano^101^	2011			×					
*R*_s_		Dongjiang River Basin (China)	O	Wang et al.^80^	2012								PCNs
*R*_s_		Landfill site (Bursa, Turkey)	O	Esen^102^	2013			×		×			
*R*_s_		Athabasca oil sands (Alberta, Canada)	O	Harner et al.^57^	2013				×				
*R*_s_		Urban sites (Chicago, USA)	O, M	Petrich et al.^51^	2013					×			
*R*_s_		Agricultural Community (Yakima Valley, USA)	C, O	Armstrong et al.^85^	2014	×							
*R*_s_		Masaryk University (Brno, Czech Republic)	O	Bohlin et al.^103^	2014		×	×	×	×	×		
*R*_s_		Masaryk University (Brno, Czech Republic)	I	Bohlin et al.^58^	2014		×	×	×	×	×		
*R*_s_		Urban sites (Gyeonggi-do, South Korea)	O	Heo and Lee^104^	2014					×	×		
*R*_s_		Guangzhou Institute of Geochemistry (China)	O	Li et al.^105^	2014		×						
*R*_s_		Sygera Mountain (China)	O	Ren et al.^106^	2014			×		×			
*R*_s_		Intensive agriculture (Argentina)	O	Astoviza et al.^64^	2016	×							
*R*_s_		Semirural area (Bursa, South Korea)	O	Evci et al.^107^	2016				×				
	K	Chamber and computational method	C, M	Parnis et al.^87^	2016				×				
*R*_s_		Background, agricultural, semiurban, urban and industrial (Bursa, Turkey)	O	Birgül et al.^108^	2017					×			
	K	Chamber study	C	Francisco et al.^60^	2017			×	×				
*R*_s_		Source sectors (São Paulo, Brazil)	O	Francisco et al.^71^	2017					×	×		
	K	Comparison of model and experiment values	M, O	Okeme et al.^109^	2017		×		×	×			
*R*_s_		Uludag University (Bursa, Turkey)	O	Sakin et al.^70^	2017					×			
*R*_s_		Central Tibetan Plateau (China)	O	Wang et al.^67^	2017			×					
*R*_s_		Residential homes (Iowa, USA)	I	Herkert et al.^110^	2018					×			
*R*_s_		GAPS network (Global)	O	Herkert et al.^22^	2018					×			
*R*_s_		Guangzhou Institute of Geochemistry (China)	O	Jiang et al.^74^	2018								monosaccharides
*R*_s_		Suburban background (Brno, Czech Republic)	I, O	Karásková et al.^72^	2018							×	
*R*_s_		Industrial & residential (Pearl River Delta, China)	O	Li et al.^75^	2018								trace metals
*R*_s_		Computer laboratory (University of Toronto, Canada)	I	Okeme et al.^111^	2018		×	×					phthalates
*R*_s_		Western Tibetan Plateau (China)	O	Zhang et al.^112^	2018			×		×			
*R*_s_		Pearl River Delta (China)	O	Zhang et al.^68^	2018				×				
*R*_s_		Agricultural regions (Antalya, Türkiye)	O	Can-Güven et al.^113^	2019			×		×			
	K	Laboratory study	C	Saini et al.^66^	2019		×	×					
*R*_s_	K	Chamber study	C	Tromp et al.^61^	2019		×	×	×	×			phthalates, musk
*R*_s_		Subtropical/tropical sites (Nairobi, Kenya & Accra, Ghana)	O	Bohlin-Nizzetto et al.^21^	2020		×	×	×	×	×		
*R*_s_		University campuses (Beijing, China)	I	Meng et al.^76^	2020								phthalates
*R*_s_		MSWI plant (China)	O	Li et al.^114^	2021		×			×	×		
	K	Urban (Toronto, Canada)	O	Niu et al.^115^	2021		×						CPs
*R*_s_		EMEP and MONET sites (Europe & Arctic)	O	Kalina et al.^91^	2022		×	×	×	×			
*R*_s_		Central Catalan Pyrenees (Andorra)	O	Prats et al.^116^	2022		×		×	×			
*R*_s_		Urban sites (Toronto, Canada)	O	Zhang et al.^117^	2022								EC
*R*_s_		Australia, China, Vietnam	O	He et al.^118^	2023		×						CPs
*R*_s_		Urban (Tarragona, Spain)	O	García-Garcinuño et al.^119^	2024		×		×				HPVCs
	SIP -PAS (including SIP-PAS and SIP-PAS + PUF–PAS)												
*R*_s_	K	Office building (Toronto, Canada)	I	Shoeib et al.^24^	2008							×	
*R*_s_		Urban site (Toronto, Canada)	O	Genualdi et al.^120^	2010							×	
*R*_s_		Residential & nonresidential building (Germany)	I	Langer et al.^121^	2010							×	
*R*_s_		Home (Vancouver, Canada)	I	Shoeib et al.^122^	2011							×	
*R*_s_		GAPS site	O	Koblizkova et al.^123^	2012		×	×					HCB, PeCB
*R*_s_		GAPS site	O	Koblizkova et al.^65^	2012	×							
*R*_s_		Hazelrigg field station (Lancaster University, UK)	O	Schuster et al.^124^	2012		×	×		×			
*R*_s_	K	Urban (Toronto, Canada)	O	Ahrens et al.^82^	2013							×	
*R*_s_		Semiurban meteorological station (Toronto, Canada)	O	Ahrens et al.^125^	2014								cVMSs, lVMSs
*R*_s_		Living room (South Korea)	I	Kim and Park^126^	2014							×	vHOPs
*R*_s_		University of Toronto (Canada)	I	Saini et al.^127^	2015		×						phthalates
*R*_s_		Roof top (Beijing, China)	O	Liu et al.^84^	2016		×						
*R*_s_	K	Urban (Toronto, Canada)	O	Abdollahi et al.^88^	2017		×						
	PAS-DD												
		Urban (Toronto, Canada)	O	Eng et al.^27^	2013				×				
*R*_s_		Source sectors (Toronto, Canada)	O	Gaga et al.^34^	2019								trace metals
	modeling/chamber/computational studies												
	K	Sorption properties of polyurethane foams	C	Kamprad and Goss^95^	2007								up to 100 VOCs
*R*_s_		CFD simulations with wind tunnel-experiments	C, M	May et al.^59^	2011					×			
*R*_s_		Meteorology-derived *V*_eff_ model	M	Herkert et al.^128^	2016					×			
	K	Chamber experiments with COSMO-RS modeling	C, M	Parnis et al.^87^	2016				×				
	K	COSMO-RS modeling	M	Parnis et al.^86^	2016				×				
		Chamber study (sorption/uptake)	C	Francisco et al.^60^	2017			×	×				
	K	Comparison of estimation methods (model evaluation)	M	Okeme et al.^109^	2017		×	×	×	×			
*R*_s_		CFD for sampler/flow fields	M	Herkert and Hornbuckle^38^	2018					×			
	K	Direct measurement with generator column approach	C	Saini et al.^66^	2019		×	×					
*R*_s_	K	Chamber study	C	Tromp et al.^61^	2019		×	×	×	×			phthalates, musk
	K	Models: linear (pp-LFERs and QSPRs-MLR) and nonlinear methods (QSPRs-ANN)	M	Gu et al.^62^	2021	Benzenes, PAHs, PCBs, alcohols, ketones, esters, ethers, nFRs, pesticides, musk							
	K	Models: linear (QSPRs-MLR) and nonlinear methods (QSPRs-ANN and QSPRs-SVM)	M	Zhu et al.^63^	2021	PAHs, benzenes, esters, musk, PCBs, pesticides							
*R*_s_	K	Graphical modeling tools	M	Li et al.^89^	2022	SVOCs							
	K	COSMOtherm	M	South et al.^129^	2022	MCCPs, LCCPs							

a Parameter: *R*_s_ = sampling rate and *K* = partition coefficients between the sampling medium and air (*K*_PUF‑A_, *K*_SIP‑A_).

b Setting: I = indoor, O = outdoor, M = model, and C = chamber study.

c Acronyms for all chemicals and other terms are defined in Table 6.

*R*_s_ derived from AAS and DC-based approaches are presented in eqs 4 and 5, respectively. *R*_s_ in the linear uptake regime from AAS is calculated as

4 $$R_{s}=\frac{M_{PUF}}{C_{A}\times t}$$

For the DC method, *R*_s_ is obtained using^56^

5 $$\begin{array}{l} R_{s}=k_{A}\times A_{PUF}=\frac{-\ln\left(\frac{C_{DC}}{C_{DC,0}}\right)\times D_{film}\times K_{PUF-A}}{t}\times A_{PUF}\\ K_{PUF-A}=K_{PSM-A}\times \rho_{PUF} \end{array}$$

where *C*_DC,0_ and *C*_DC_ represent the initial and final masses of the DC (ng/sample), respectively, *D*_film_ is the effective film thickness of the PUF disk (*m*), *K*_PSM‑A_ ^56^ is the passive sampling medium-air partition coefficient (m^3^/g), and ρ_PUF_ is the PUF density (g/m^3^).

Over the past two decades, most experimental studies have focused on PACs, PBDEs, and PCBs. Partition coefficients have been determined through theoretical estimations,^5^ computational modeling approaches such as Conductor-like Screening Model for Real Solvents (COSMO-RS),^86,87^ or quantitative structure–property relationship (QSPR),^63^ poly parameter linear free energy relationship (pp-LFER), and direct experimental measurements.^66,88^ Recent modeling studies^62,63^ have substantially expanded the range of compounds with characterized partitioning behavior. With the suite of emerging chemicals continuing to expand, it is increasingly important to extend the mechanistic characterization, i.e., partitioning coefficient, of passive samplers to include these compounds, ensuring accurate uptake estimates and comparability across studies.

Three tools are publicly available for characterizing the chemical-specific effective air volume and sampling rates:

1. GAPS Network Template:^56^ this approach integrates empirical K_PUF‑A_ and K_SIP‑A_ data for calculating effective air volumes and air concentrations.
2. Iowa Superfund Research Program (ISRP) PUF–PAS Sampling Rate Model (pufpasvolume.org): Developed by Herkert et al.,^22^ which utilizes National Aeronautics and Space Administration Modern-Era Retrospective analysis for Research and Applications meteorological data sets to account for temperature, humidity, and especially wind speed effects on linear-phase sampling rates and effective sampling volume.
3. Graphical tools^89^ based on the PAS-SIM model:^90^ These tools integrate key physicochemical and environmental variables, including temperature, wind speed, molecular diffusivity, phase-partitioning parameters, and deployment duration, to delineate SVOC uptake regimes (linear, curvilinear, or equilibrium). These model-generated plots yield quantitative predictions of sampling rates, the temporal extent of the linear uptake phase, equilibration time scales, and the fractional loss of DCs. By explicitly illustrating the sensitivity of uptake behavior to uncertainties in input parameters, these tools provide a mechanistic basis for optimizing PUF–PAS deployment strategies and for improving the interpretation of time-integrated SVOC measurements.

Bohlin-Nizzetto et al.^21^ and Kalina et al.^91^ evaluated *R*_s_ estimates from the GAPS Network template, the ISRP PUF–PAS Sampling Rate Model, and AAS across MONET sites, finding general agreement with AAS except for high *K*_OA_ compounds, where deviations were primarily due to sampler-specific model parametrization rather than temperature effects. Both models captured temperature-dependent behavior, including increased *R*_s_ values for intermediate-volatility SVOCs and reduced effective sampling volumes for low-volatility compounds. The GAPS template predicted higher *R*_s_ for high *K*_OA_ chemicals, reflecting its inclusion of particle-phase uptake, whereas the ISRP model is calibrated only for gas-phase PCBs and α-HCH. Both models tend to overestimate concentrations at sites with persistently high wind speeds (>4 m/s). Overall, these models facilitate global standardization of passive sampling rates and improve the accuracy of chemical concentration estimates under diverse meteorological conditions.

### 3.5 Performance Evaluations and Intercomparison Studies

Comprehensive evaluations of the PUF–PAS, SIP–PAS, and PAS–DD samplers have been conducted under diverse environmental settings, including indoor,^99^ outdoor,^144^ and modeled conditions^149^ (Table 2). These studies have systematically investigated key parameters influencing sampler performance, such as sampler configuration (e.g., single vs dual PUF disks),^147^ comparative assessments among different sampler types (passive vs active air sampler),^69,133^ housing design,^38,132–134,139,148^ foam density,^49,136^ chemical degradation,^141,142,145^ and calibration procedures.^20,52^ Duplicate samplers were also deployed to evaluate sampling reproducibility and variability.^93^ Environmental factors, such as temperature, wind speed, and precipitation, have also been demonstrated to significantly influence sampling rates and mass transfer processes.^20,21,38,89,113,128,135,139,149^

**Table 2:** Studies Evaluating Sampler Design, Performance, and QA/QC Practices for PUF–PAS, SIP-PAS, and PAS-DD

parameter^a^		sampler design and/or performance considerations^b^	setting^b^	reference	year	CUPs^c^	FRs	OCPs	PACs	PCBs	PCDD/Fs	PFAS	others^c^
PUF–PAS and/or SIP-PAS													
*R*_s_		duplicate passive samplers deployed to evaluate sampling reproducibility and variability	O	Waite et al.^93^	2005	×							
		comparison of AAS and PUF–PAS (Valasske Mezirici, Czech Republic)	O	Klánová et al.^130^	2006			×	×	×			
*R*_s_		effect of ambient wind conditions and velocity gradients on uptake rate (Computational fluid dynamic model)	M	Thomas et al.^131^	2006					×			
*R*_s_		variability in uptake rates contributed by wind effect and different housing design (Wind tunnel/model)	C, M	Tuduri et al.^132^	2006					×			
*R*_s_	K	effect of wind speed, temperature, shelter/degree of exposure and sampler design on uptake rate (Lancaster, UK)	O	Chaemfa et al.^133^	2008			×		×			
		comparison of AAS, XAD-PAS and PUF–PAS (Costa Rica)	O	Gouin et al.^97^	2008			×					
*R*_s_		sampling rate comparison between fully- and part-sheltered passive air sampler design (Bedfordshire, UK)	I	Harrad and Abdallah^134^	2008		×						
*R*_s_		effect of temperature and wind speed on sampling rate (Košetice, Czech Republic)	O	Klánová et al.^135^	2008			×	×	×			
		comparison of AAS, SIP-PAS and PUF–PAS (carpeted library, University of Toronto, Canada)	I	Shoeib et al.^24^	2008							×	
*R*_s_		Effect of wind speed, temperature, rainfall and sampler design on sampling rates (Lancaster University, UK)	O	Chaemfa et al.^69^	2009		×						
*R*_s_		Effect of foam densities on uptake rate (Lancaster, UK)	O	Chaemfa et al.^49^	2009		×			×			
		Effect of wind speed and foam density on aerosol entrapment	O, M	Chaemfa et al.^136^	2009								aerosol
*R*_s_		effect of wind speed on sampling rates (Uppsala, Sweden)	O	Moeckel et al.^78^	2009					×			
*R*_s_		performance evaluation of PAS for quantification, focusing on precision, uptake kinetics, and shelter design effects in occupational and residential air	I	Bohlin^137^	2010				×				
		comparison of four active and passive sampling techniques (Ontario, Canada)	O	Hayward et al.^138^	2010			×					
*R*_s_	K	effect of sampling housing vs ambient temperature on sampling rates (subtropical and temperate locations, Australia)	O	Kennedy et al.^139^	2010					×			
*R*_s_		sampling rate variability contributed by linear uptake phase, particle uptake, wind speed and direction, temperature effects, calibration methods, validation method of sampling rate, use of homologue specific sample rates (Toronto, Canada)	O	Melymuk et al.^20^	2011		×		×	×			PCMs
*R*_s_		Comparison of active and SIP-PAS measurements in the UK and Norway	O	Schuster et al.^124^	2012		×			×			
*R*_s_		comparison of active, PUF–PAS and SIP-PAS measurements in semiurban meteorological station in Toronto. Different chamber configurations were also evaluated	O	Ahrens et al.^82^	2013							×	
		effect of ambient temperature on PAS housing; the stainless-steel housing shows the lowest internal temperature	O	Vardar et al.^140^	2013								
*R*_s_	K	comparison of active, PUF–PAS and SIP-PAS measurements	O	Ahrens et al.^125^	2014								VMSs
*R*_s_		effect of temperature, relative humidity and wind speed on sampling rates (Washington, USA)	C, I, O	Armstrong et al.^85^	2014	×							
*R*_s_		critical evaluation of the performance and limitations of PUF–PAS in outdoor air monitoring for SVOCs	O	Bohlin et al.^103^	2014		×	×	×	×	×		
*R*_s_		evaluation of sampling performance: (1) detection and minimum exposure times; (2) precision; (3) fingerprinting; (4) Rs; (5) sampling of particle associated compounds	I	Bohlin et al.^58^	2014		×	×	×	×	×		
*R*_s_		comparison of PUF–PAS and SIP-PAS for volatile HOCs	O	Kim and Park^126^	2014								HOCs
		stability of PACs upon O_3_ exposure	O	Jariyasopit et al.^141^	2015				×				
		evaluation of the particle infiltration efficiency of three passive samplers and the PS-1 active air sampler	O	Markovic et al.^6^	2015								particle
		assessment of PAH loss by the effect of temperature	O	Domínguez-Morueco et al.^142^	2016				×				
*R*_s_		effect of wind speed, temperature, and equilibrium partition on PUF–PAS sampling volumes (USA, UK, Canada)	O, M	Herkert et al.^128^	2016					×			
*R*_s_		assessment of the sorption comparability of PUF and SIP disks	O	Liu et al.^84^	2016		×						
*R*_s_		comparison of PUF, GFF/PUF and AAS (Three offices at Tsinghua University, Beijing, China)	I	Newton et al.^143^	2016		×						
*R*_s_		evaluation of the uncertainty in PUF–PAS derived air concentrations using long-term air monitoring data	O	Holt et al.^144^	2017			×	×	×			
		evaluation of within-sampler degradation of SVOCs during active and passive air sampling	O	Melymuk et al.^145^	2017		×	×	×	×			
*R*_s_		effect of windspeed on double vs half-dome PUF–PAS	I, M	Herkert and Hornbuckle^38^	2018					×			
*R*_s_		degradation of monosaccharide in PUF–PAS	O	Jiang et al.^74^	2018								monosaccharides
*R*_s_		evaluation of the performance of PUF–PAS and XAD2-PAS by comparing AAS	I, O	Karásková et al.^72^	2018							×	
*R*_s_		comparability of five years of AAS and PUF–PAS data across six European sites	O	Li et al.^75^	2018								metals
*R*_s_		evaluation of three PASs by comparing AAS	I	Okeme et al.^111^	2018		×						phthalates
*R*_s_		comparability of 5 years data from AAS and PUF–PAS at the six sites in Europe	O	Kalina et al.^146^	2019		×	×		×			
*R*_s_		PUF–PAS uptake performance in subtropical/tropical and temperate climate zones	O	Bohlin-Nizzetto et al.^21^	2020		×	×	×	×	×		
		global intercomparison of PUF–PAS to evaluate sources of variability in SVOC measurements	O	Melymuk et al.^23^	2021		×	×	×	×			
		data comparison for AAS and PUF–PAS at the six sites in Europe	O	Kalina et al.^91^	2022		×	×	×	×			
		new method for measuring airborne elemental carbon using PUF–PAS	O	Zhang et al.^117^	2022								EC
		assessment of single versus dual PUF in passive air samplers	O	Caliskan et al.^147^	2024				×				
*R*_s_		sampling rate comparison across no, single, and double-bowl housings	I	Vojta et al.^148^	2024		×	×	×	×			
		review of calibration approaches for different passive air samplers	RV	Tuduri et al.^52^	2012	-							
PAS-DD													
		dry deposition comparison between the PAS-DD and PUF-disk sampler	O	Eng et al.^27^	2013				×				
		effect of wind speeds and various angles of attack on particle size deposition on the PAS-DD sampler	M	Sajjadi et al.^149^	2016								aerosol

a Parameter: *R*_s_ = sampling rate and *K* = partition coefficients between the sampling medium and air (*K*_PUF‑A_, *K*_SIP‑A_).

b Setting: C = chamber (or wind tunnel), I = indoor, O = outdoor, M = model, and RV = review.

c Acronyms for all chemicals and other terms are defined in Table 6.

Computational fluid dynamics (CFD) simulations have been employed to investigate the aerodynamic behavior and sampling efficiency of passive air samplers, such as the PUF–PAS and PAS–DD.^59,149^ These simulations provide a mechanistic understanding of how airflow patterns, sampler geometry, and environmental conditions influence contaminant uptake and particle deposition. For PUF–PAS, CFD analyses have been used to simulate contaminant transport and uptake under varying wind regimes. Results demonstrated the presence of strong internal velocity gradients within the sampler housing, which create spatially nonuniform mass transfer conditions across the PUF disk. When the sampling rate was estimated using the average air velocity at the PUF surface, modeled values were in close agreement with experimental measurements.^131^ For the PAS–DD, Sajjadi et al.^149^ performed CFD simulations using ANSYS FLUENT to examine the deposition behavior of particles (0.5–10 μm) under different sampler geometries, wind speeds, and angles of attack. Their findings indicated that deposition velocity increased systematically with both particle size and wind speed and was highly sensitive to sampler geometry and flow orientation. The simulations also revealed a minor influence of the PUF surface roughness on deposition behavior. Overall, the modeled deposition velocities showed a strong consistency with experimental observations.

Chaemfa et al.^133^ examined the effects of wind speed, degree of shelter, and sampler height, finding comparable uptake rates among the GAPS network, Lancaster University, and MONET configurations when deployed under unobstructed airflow (<5 m height) and observing no significant differences in reported chemical concentrations. Markovic et al.^6^ compared particle infiltration efficiency among different PUF–PAS designs using a wide-range particle spectrometer. The PS-1 HiVol sampler, PUF–PAS (GAPS), and PUF–PAS (Lancaster University) captured ambient particles effectively, with particle infiltration efficiency of 89.6 ± 13.4%, 91.5 ± 13.7%, and 103 ± 15.5%, respectively, whereas the MONET configuration exhibited a substantially lower particle infiltration efficiency of 54 ± 8.0%, indicating the influence of housing geometry on particle-phase sampling. Advances in analytical methodology, such as novel approaches for measuring elemental carbon (EC),^117^ have further informed sampler capabilities.

Building on these evaluations of sampler performance and particle infiltration, the PUF–PAS (particularly the double-dome GAPS design) and the PAS-DD can be distinguished primarily by their housing geometries, which control particle transmission and, consequently, the interpretation of the measured data. The PUF–PAS employs a semienclosed chamber that functions as a size-selective inlet, maintaining high infiltration efficiency for fine particles (on the order of ∼90%) while largely excluding the coarse fraction (>4 μm).^6^ As a result, measurements from PUF–PAS represent ambient air concentrations of gases and suspended fine particles. In contrast, the open, parallel-plate design of PAS-DD allows collection of the full particle size spectrum, including coarse particles that dominate gravitational settling, making it more suitable for quantifying dry deposition fluxes rather than air concentrations. This design leads to substantially greater collected particle mass (approximately 3.5–5 times higher for particle-bound chemicals) and correspondingly higher calculated deposition velocities (up to ∼0.8 cm/s).^27^ A key limitation common to both approaches is particle retention: evidence indicates that using PUF disks alone is preferable to adding GFF because the porous foam matrix can entrap particles tens of micrometers deep, reducing particle blow-off and resuspension artifacts that may otherwise affect measurements, particularly in more open sampler configurations.^27,31^

The PUF–PAS and SIP-PAS were evaluated against active air samplers. In southern Ontario, four sampling systems—two active (high- and low-volume pumps) and two passive (PUF and XAD-resin)—were deployed from March 2006 to September 2007 to measure nine pesticide concentrations with different sampling frequencies (biweekly to annually) and durations (24 h to 1 year). Agreement among the methods improved with longer averaging periods. Annual averages for other pesticides were within a factor of 2.5 and not statistically different.^138^ Schuster et al.^124^ conducted two one-year field studies using SIP-PAS for PCB and PBDEs air sampling. SIP disks, as a higher-capacity alternative to PUF disks, were first validated for the linear uptake of volatile perfluorinated compounds (PFCs). In the first study, SIP disks were deployed at a rural UK site for 35–350 days alongside a high-volume air sampler. Linear uptake was observed for all PCBs and PBDEs, while HCB reached equilibrium after six months. In a second study, SIP disks were deployed at 10 sites across the UK and Norway for one year, yielding concentration estimates and spatial distributions consistent with long-term monitoring data.

More recently, Melymuk et al.^23^ led a global intercomparison of PUF–PAS to investigate sources of variability arising from sampler design and analytical methodology. This intercomparison was critical given that PUF–PAS represents the most widely used passive air sampler globally, yet no standardized protocols exist for housing construction, analytical methods, or quality assurance/quality control (QA/QC) procedures. The intercomparison is also important to evaluate if the measurement results are comparable among international monitoring programs globally. Twelve distinct PUF–PAS housing designs deployed across participating research groups demonstrated that geometric differences, such as the spacing between upper and lower bowls, could account for up to 50% variation in measured concentrations. Moreover, 15 laboratories analyzed identical PUF disks using in-house analytical protocols (e.g., analytical methods and sample handling practices) to quantify the number of OCPs, PAHs, and PBDEs, revealing variability exceeding an order of magnitude. These findings indicate that differences in laboratory procedures and sample handling introduce substantially more uncertainty than sampler geometry, emphasizing the importance of standardized methodologies in assessing spatial or temporal trends. These results indicate that interlaboratory differences can substantially compromise the comparability of passive air sampling data across monitoring programs. The use of a central laboratory is recommended to minimize uncertainties.

In summary, the PUF–PAS operates as a diffusion-controlled sampler, where the uptake flux is dictated by air-side boundary-layer transport and PUF–air partitioning. The *R*_s_ value, dependent on environmental dynamics and sampler geometry, defines the effective air volume for concentration estimation. For most semivolatile and involatile chemicals (including those associated with ambient aerosols), the sampling rate is proportional to the surface area of the sampler and approximately 4 m^3^/day (for GAPS-type PUF disks) and remains in the linear-phase region for deployment periods of months or more. So effective sample air volumes are easily estimated as 4 m^3^/day × days of deployment, whereas for more volatile chemicals (i.e., log *K*_OA_ < 8) estimation of effective air volumes requires knowledge of the physicochemical properties of the chemicals in question, validated calibration, and rigorous QA/QC. As passive air sampling expands to include contaminants of emerging concern, continued refinement of *K*_PUF‑A_ and *K*_SIP‑A_, interlaboratory harmonization, and integration of meteorological modeling will be essential to enhance the robustness and comparability of global PUF–PAS monitoring data.

## 4 Application of PUF–PAS, SIP-PAS, and PAS-DD

### 4.1 Overview

PUF–PAS have become well-established as cost-effective and versatile tools for monitoring atmospheric pollutants across a wide range of environments. Since their initial application^4^ in 2000 for measuring air concentrations of POPs and emerging chemicals, their use has expanded to novel areas such as bioaerosol monitoring of airborne bacteria and fungi^150^ and the collection of environmental DNA (eDNA) to track bat activity in 2025.^151^ This section reviews three widely used sampler designs—PUF–PAS, SIP-PAS, and PAS-DD—with an emphasis on their applications in outdoor and indoor air monitoring, emission source characterization, and health studies.

### 4.2 Target Analytes

The PUF–PAS was originally developed for the measurement of POPs, with its first applications focused on PCBs and polychlorinated naphthalenes (PCNs).^4^ Since then, its analytical scope has expanded substantially, from legacy POPs^47,152^ to a wide spectrum of emerging contaminants,^153^ making it one of the most versatile tools for long-term and spatially extensive atmospheric monitoring. Over the past two decades, the PUF-based PAS has been employed to measure a broad range of compound classes, including:

(1) The initial 12 POPs: PCBs,^4^ OCPs,^47^ and PCDD/Fs;^73^
(2) New POPs: PBDEs,^152^ Dechlorane Plus (DP),^154^ dicofol,^155^ dioxin-like PCBs (dl-PCBs),^156^ hexabromocyclododecane (HBCDD),^134,157^ lindane,^158^ short-chain chlorinated paraffins (SCCPs) and medium-chain chlorinated paraffins (MCCPs),^159^ perfluorooctane sulfonate (PFOS) and PFOA,^160^ PCNs,^161^ and endosulfan;^64,162^

(3) Emerging chemicals: CUPs,^97^ novel Flame retardants (nFRs),^163^ OPEs,^85^ phthalates,^127^ plasticizers,^164^ PACs,^9,165^ polybromobenzenes (PBBz),^166^ PFAS^167^ including perfluoroalkyl acids (PFAAs),^168^ and FTOHs,^169^ synthetic musk compounds (SMCs),^170^ volatile methyl siloxanes (VMSs),^171^ and UV-filters;^172^
(4) Other compounds: particulate matter-associated compounds,^34,173^ natural brominated anisoles,^174^ monosaccharides,^74^ and brake and tire wear-associated chemicals.^175^

Each compound class supports specific monitoring objectives—ranging from assessing human exposure,^176^ long-range atmospheric transport,^177^ deposition,^31^ and transformation processes^178^ to identifying emission sources and chemical fate.

### 4.3 Ambient Air Monitoring and Research

#### 4.3.1 Network

PUF–PAS, SIP-PAS, and PAS-DD have been extensively applied in studies investigating ambient concentrations,^156^ spatial distributions,^179^ and atmospheric transport of POPs^180^ and emerging chemicals.^181^ The application of these samplers has evolved markedly since 2000, when PUF disks were first deployed in the Toronto gradient study^4^ to delineate the spatial extent of POP concentrations in urban air. By 2024, the use of PUF–PAS and SIP-PAS had expanded to national, international, and global monitoring programs, encompassing studies across multiple spatial scales from local or source-oriented investigations to urban, regional, national, and global assessments.

Several regional monitoring programs have integrated PUF–PAS into their air quality surveillance frameworks, including the University of Iowa’s Superfund Research Program on sources of airborne PCB congeners,^182^ the Great Lakes Basin (GLB) network,^92^ the Northern Contaminants Program (NCP),^183^ and the Spanish Monitoring Program (SMP) operated by two institutes (Centre for Energy, Environmental and Technological Research for inner sites and Spanish National Research Council for outer locations).^17,184,185^ At global and international scales, multiple long-term monitoring initiatives have been established to quantify atmospheric levels of POPs and emerging chemicals. These programs aim to elucidate spatial and temporal concentration trends, thereby advancing our understanding of the fate, long-range transport, and deposition of these compounds in the environment.

Key international initiatives and their operational timeframes include

(1) The Global Atmospheric Passive Sampling (GAPS) Network (2002–present)^14,55,154,186–188^

Initially operational at selected global sites as early as 2002 as a pilot study for establishing proof of concept,^55^ the formal and expanded GAPS network was later established in 2004 by Environment and Climate Change Canada (ECCC). GAPS represents the first globally coordinated initiative to monitor POPs and other SVOCs using GAPS-type PUF–PAS samplers. The network operates at more than 50 sites worldwide, spanning diverse environments—from densely populated urban centers to remote polar regions. Data generated from GAPS have provided critical insights into global transport pathways, interhemispheric exchange, and long-term temporal trends of both legacy and emerging POPs. Furthermore, the GAPS network contributes to the Global Monitoring Plan (GMP) under the UNEP, supporting the Stockholm Convention on POPs and its Effectiveness Evaluation (Article 16) by supplying harmonized, globally representative data on atmospheric POP concentrations.

(2) The GAPS–Megacities Network (2018–present)^175,189^

Launched in 2018 as an extension of the GAPS program, this initiative focuses on major urban centers globally.^189^ It examines spatial gradients of POPs and emerging chemicals within 23 megacities, linking emissions, urban morphology, and atmospheric dispersion processes.

(3) MOnitoring NETwork (MONET) (2003–present)^15,190,191^

Initiated in 2003 in the Czech Republic^192^ through a successful collaborative pilot study (employing PUF–PAS and hivol air sampler) with the University of Lancaster and ECCC. MONET, operated by Research Centre for Toxic Compounds in the Environment (RECETOX), Masaryk University, employs PUF–PAS samplers at multiple sites in 44 countries on three continents—Central and Eastern Europe (CEE), Africa, and Asia.^190,193^ It supports the implementation of the Stockholm Convention by providing comparable long-term data on atmospheric POP levels and assisting in identifying regional emission hotspots.

(4) UNEP- Global Environment Facility (GEF) Projects^18^

Since 2005, UNEP has developed a National Implementation Plan and implemented a series of GEF-funded GMP projects to strengthen POPs monitoring capacity in developing countries. From 2008 to 2011, UNEP coordinated the first GMP phase (GMP1), during which PUF–PAS samples were collected from 32 countries across Africa, the Group of Latin America and Caribbean Countries (GRULAC) region, and the Pacific Islands, establishing the foundation for the second phase (GMP2, 2016–2020).^12,194,195^ These coordinated efforts have generated essential air monitoring data for evaluating the effectiveness of the Stockholm Convention. PUF–PAS, typically deployed for three-month exposure periods, was used in all participating countries, while active air samplers were tested in selected locations. Data from these projects are archived in the Stockholm Convention GMP database, supporting global assessments of POPs trends.

#### 4.3.2 Trend

Following the establishment of coordinated monitoring networks using PUF–PAS, few studies have generated long-term temporal data of POPs in ambient air. Key studies include:

(i) Global (GAPS, 2005–2014)^14^

The GAPS network, established in 2004 at 55 sites worldwide, provides 5–10 years of consistent data from 40 sites, forming the first comprehensive global assessment of POP concentrations and trends. Results show that PCBs, endosulfan, and HCHs have declined at most locations, reflecting reduced emissions, stricter regulations, and depletion of old stockpiles. In contrast, other OCPs such as chlordanes, heptachlor, and dieldrin exhibit stable or slowly declining trends, suggesting a shift from primary emissions to secondary revolatilization.

(ii) Africa (MONET, 2008–2019)^193^

The MONET network assessed long-term (2008–2019) POP trends across nine African countries (Congo, Ghana, Ethiopia, Kenya, Mali, Mauritius, Morocco, Nigeria, and Sudan). Atmospheric concentrations of 20 POPs—including aldrin, chlordane, DDT, HCHs, PCBs, PBDEs, PCDD/Fs, PFOA, and PFOS—were monitored. Most legacy POPs (e.g., PCBs, DDT, HCHs, and endosulfan) showed significant declines over the decade, though rates varied among sites. Conversely, PCDD/Fs and PBDEs remained stable or increased at certain locations, likely due to ongoing emissions from open waste and electronic waste (e-waste) burning. Back-trajectory modeling indicates that elevated levels are mainly associated with local sources, whereas low concentrations at Mt. Kenya reflect continental background influenced by long-range transport.

(iii) Europe (MONET, 2003–2019)^15^

MONET also analyzed 15 years (2003–2019) of POP data across 32 sites in 27 European countries, covering 20 compounds. Temporal trends indicate that concentrations of most POPs have declined significantly over the past 15 years, with median annual decreases ranging from −8.0 to −11.5% (halving times of 6–8 years) for ∑_6_PCB, ∑_17_PCDD/F, HCB, pentachlorobenzene (PeCB), and ∑_9_PBDE. Furthermore, no statistically significant differences were observed in either the trends or the concentrations of specific POPs at sites in Western Europe and Others Group (WEOG) compared to sites in the Central and East Europe group (CEE), which suggests relatively uniform compound-specific distribution and removal at the continental scale.

(iv) Developing Countries (UNEP-GEF Projects, 2010–2011 and 2017–2019)^18^

Under the UNEP-GEF projects, two monitoring phases (GMP1: 2010–2011; GMP2: 2017–2019) were conducted for POP measurements. Across both campaigns, 423 PUF–PASs for OCPs and PCBs and 242 for dioxin-like POPs were analyzed. Comparative analyses (same countries and compounds) included 194 PUF–PASs for OCPs, 297 for PCBs, 158 for PCDD/Fs, and 153 for dl-PCBs. Indicator PCBs and dioxin-like POPs were consistently detected across all sites and sampling periods, showing median declines of about 30%. In contrast, HCB increased by roughly 50%, while DDT remained the most abundant compound despite an overall >60% decrease, largely driven by reductions in the Pacific Islands region.

Two coordinated PUF–PAS campaigns^179,196^ —covering 86 European background sites in 2006 and 101 sites across 33 countries in 2016—characterized the spatial and temporal dynamics of POPs across Europe. HCB exhibited both the highest atmospheric concentrations and the only statistically significant increase over the 10 year interval and was the sole POP showing a positive correlation with latitude, indicating a stronger influence of secondary emissions and cold-temperature partitioning processes. In contrast, most other targeted POPs displayed maximum concentrations in southern Europe and consistent declines between 2006 and 2016. The magnitude of temporal change varied regionally: γ-HCH showed the steepest decline in southern Europe, whereas α-HCH decreased most markedly in Central–Eastern Europe. Elevated levels of the OCP transformation products and diagnostic isomer ratios provided further evidence that contemporary atmospheric burdens are dominated by remobilization of legacy residues rather than ongoing primary emissions.

In summary, these monitoring efforts show sustained long-term declines for most legacy POPs worldwide, while emerging contaminants and regions influenced by open burning exhibit stable or increasing concentrations. Data from these networks have also revealed well-defined temporal trends, regional concentration gradients, and signatures of long-range atmospheric transport,^98^ providing critical empirical constraints for improving global models of chemical fate, deposition fluxes, and ecosystem exposure.

Since their development in 2000, the PUF–PAS, SIP–PAS, and PAS–DD have become widely adopted tools for ambient air research and monitoring, with more than >300 published studies to date. Figure 4 summarizes ambient air studies using PUF–PAS, SIP-PAS, or PAS-DD across UN regions and at a global scale published between 2004 and 2024. Publication activity has expanded substantially, increasing from 39 studies in 2005–2009 to more than 69 in 2010–2014, and exceeding 200 studies between 2015–2024—approximately double the output from the preceding decade. Global and multiregional studies have grown particularly rapidly, with nearly twice as many publications in 2020–2024 compared to 2015–2019. Research output from the Asia–Pacific region shows a marked upward trajectory, reflecting intensified monitoring efforts. Major long-term networks, including the GAPS global program and MONET regional initiative, have now achieved two decades of measurement.^14,15^

**Figure 4:**
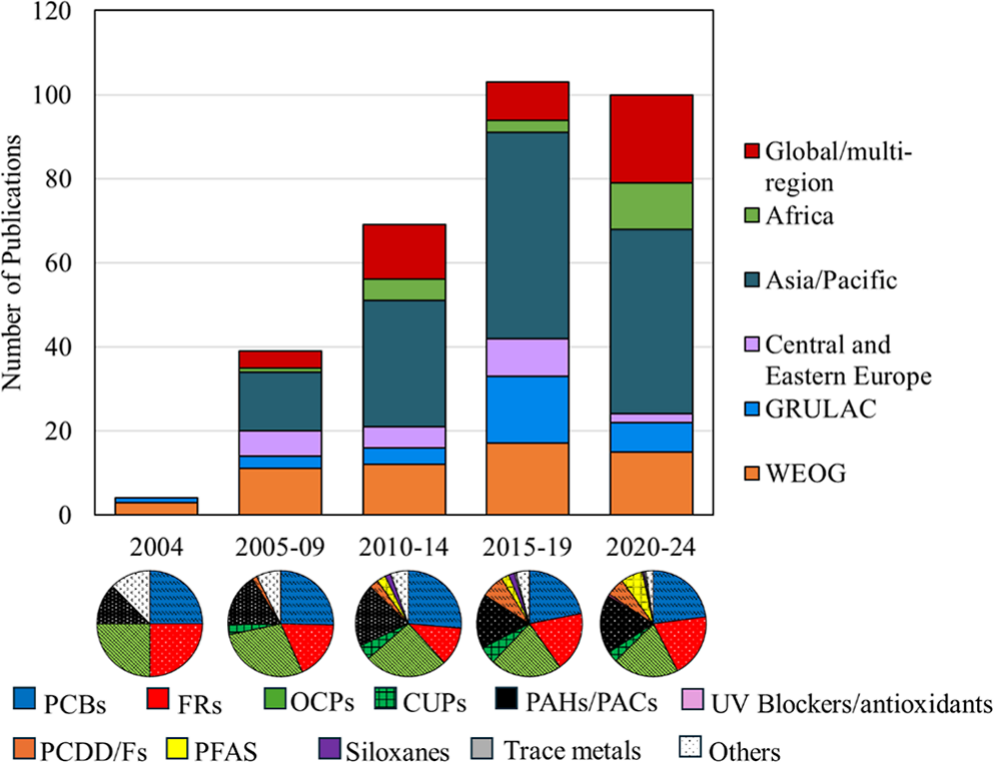
Number of ambient air research publications employing PUF–PAS, SIP-PAS, or PAS-DD samplers from 2004 to 2024 for ambient air. Publications are categorized based on the UN region of study (top panel, bar chart) and distribution of targeted POPs (bottom panel, pie charts). GRULAC and WEOG stand for Group of Latin America and the Caribbean and Western European and Other, respectively. The full list of publications is presented in Table S1.

### 4.4 Indoor Air and Occupational Exposure

Although the PUF–PAS was originally developed for monitoring POPs and other SVOCs in outdoor environments, its subsequent adaptation for indoor applications has significantly advanced our understanding of chemical behavior and human exposure within built environments. Indoor air is characterized by lower ventilation rates, limited air exchange with the outdoors, and elevated concentrations of certain contaminants due to continuous emissions from materials, furnishings, and consumer products. Under these conditions, passive samplers provide unique advantages over active systems. Their long deployment periods enable time-integrated measurements that more accurately reflect human exposure over realistic time scales and capture temporal variability not resolved by short-term active sampling.

The first adaptation of the PUF–PAS for indoor monitoring was introduced by Wilford et al.,^36^ who developed a modified open-face housing to enhance air circulation around the PUF disk under low-ventilation conditions. This design improved mass transfer and reduced boundary-layer resistance, enabling the more accurate quantification of SVOCs in stagnant indoor air. Building on this work, Barber et al.^159^ deployed the PUF–PAS to measure SCCPs and MCCPs in both indoor and outdoor environments in the United Kingdom, facilitating direct spatial and temporal comparisons between microenvironments. Similarly, Shoeib et al.^197^ used the SIP–PAS to monitor PFAS and found significantly higher indoor concentrations than outdoors, highlighting the role of indoor sources as dominant contributors to human exposure.

The expansion of passive sampling into indoor environments has been driven by the recognition that people spend more than 90% of their time indoors—at home, in schools, workplaces, and public facilities—where exposures are shaped primarily by building materials, furnishings, and product use rather than ambient air quality.^10,36,58,198^ Many legacy and emerging contaminants, including PBDEs,^199^ PCBs,^200^ OPEs,^199^ and PFAS,^168^ were historically used in consumer goods such as electronics, textiles, and furniture, and continue to volatilize or re-emit long after regulatory phase-outs. Passive samplers are ideally suited for such settings because they are silent, inexpensive, power-free, and unobtrusive, making them especially valuable for long-term monitoring, large-scale exposure assessments, and studies in sensitive or occupied environments.

In recent years, PUF–PAS and SIP–PAS have been applied in a wide range of indoor and occupational microenvironments—such as beauty salons,^201^ public bars,^202^ schools,^200,203^ hotels,^204^ public buildings,^205^ mining communities,^206^ e-waste storage facilities,^207^ chlorinated paraffin (CPs) production plants,^208^ and end-of-life vehicle processing workshops^209^ (Table 3). These investigations have quantified concentrations of legacy POPs, such as PCBs,^201,210^ OCPs,^10^ PAHs,^211^ and PBDEs,^212^ demonstrating their persistence in building materials and consumer goods decades after regulatory phase-outs.

**Table 3:** Applications of PUF–PAS and SIP-PAS in Indoor Air Monitoring and Occupational Exposure Assessment

application^a^	compounds	reference
sources and exposure in indoor and outdoor air in Ottawa, Canada	PBDEs	Wilford et al.^36^
indoor survey at Lancaster University, UK	SCCAs, MCCAs	Barber et al.^159^
occurrence, partitioning, and human exposure in indoor and outdoor air	PFAS	Shoeib et al.^221^
human exposure in Kuwait indoor air	PBDEs	Gevao et al.^222^
human exposure in indoor air in Birmingham, UK	PBDEs, PCBs	Harrad et al.^223^
factors influencing indoor air concentration variability	PCBs, PBDEs	Hazrati and Harrad^224^
review of passive sampling approaches for POPs in occupational and indoor air	PCBs, PCDD/Fs, OCPs, BFRs, PBDEs, PAHs	Bohlin et al.^225^
human exposure in Kuwait indoor air	PAHs	Gevao et al.^226^
chiral PCB signatures in outdoor/indoor air and soil in UK conurbations	PCBs	Jamshidi et al.^227^
relationships between indoor and outdoor levels and associated human exposure	PAHs, PCBs	Menichini et al.^198^
human exposure in indoor air in Birmingham, UK	HBCDD, TBBP-A	Abdallah et al.^157^
POP levels in indoor and outdoor air	PAHs, PCBs, OCPs, PBDEs	Bohlin et al.^10^
human exposure in occupational, residential, and urban environments in Greece	PBDEs	Mandalakis et al.^205^
indoor level characterization	PFCs	Shoeib et al.^24^
household exposures in a Wisconsin cohort	PBDEs	Imm et al.^228^
human exposure in a Japanese hotel indoor environment	BFRs	Takigami et al.^204^
investigation of exposure pathways and body burden	PBDEs	Toms et al.^229^
calibration of modified PUF–PAS (with GFF) for in-vehicle monitoring	BFRs	Abdallah and Harrad^40^
human exposure in private vehicles	PBDEs	Hazrati et al.^212^
indoor exposure in residential and nonresidential environments	PFCs	Langer et al.^121^
BFR concentrations in air/dust from e-waste storage sites in Thailand	BFRs	Muenhor et al.^230^
indoor sources and exposure assessment	PFCs	Shoeib et al.^122^
characterization of indoor sources, emissions, and fate	PBDEs, PCBs	Zhang et al.^231^
indoor/outdoor PFAS levels and exposure, UK	PFAS	Goosey and Harrad^153^
indoor and outdoor PFAS in South Korea	PFAS	Kim et al.^167^
indoor dust and air exposure in Vietnamese e-waste recycling sites	PCBs, PBDEs	Tue et al.^207^
inhalation and dietary PCB exposure in urban/rural cohorts	PCBs	Ampleman et al.^232^
human exposure in California indoor air	PBDEs	Bennett et al.^233^
indoor exposure in schools, homes, universities, and hospitals in Algeria	PAHs	Boudehane et al.^234^
human exposure in Guangzhou, China	BFRs	Ding et al.^235^
film–air partitioning on window glass surfaces in Beijing	SCCPs	Gao et al.^236^
concentrations, profiles, and human exposure	PBDEs	Han et al.^237^
level in a textile plant and worker exposure	PFAS	Heydebreck et al.^216^
seasonal indoor–outdoor relationships of flame retardants and PCBs	PCBs, FRs	Melymuk et al.^238^
distribution of SVOCs in residential indoor matrices	SVOCs	Melymuk et al.^239^
levels, appliance effects, and human exposure	PBDEs	Sun et al.^240^
comparison of human exposure	PBDEs, nFRs	Tao et al.^213^
comparative study of indoor contamination from three countries	BFRs	Venier et al.^214^
occupational exposure	CUPs, oxygen analogs	Gibbs et al.^241^
levels, distribution, and exposure risk of FRs in Pakistani indoor/outdoor air	nBFRs, PBDEs, DPs	Khan et al.^242^
exposure in U.S. urban and rural schools	PCBs, OH-PCBs	Marek et al.^210^
nondietary exposure to HFRs of ring-billed gulls	HFRs	Sorais et al.^42^
use of deuterium-labeled SVOCs to study transfer to air/dust	Phthalates, adipates	Sukiene et al.^243^
regional differences and partitioning in indoor air in Canada, the Czech Republic and the United States	OPEs	Vykoukalová et al.^244^
assessment of human exposure to potential contaminants	Pesticide, phytochemicals	Wang et al.^203^
children’s bedrooms	PFAS	Winkens et al.^168^
sources and occurrence in Nepalese indoor air	PBDEs, nBFRs	Yadav et al.^245^
occurrence and fate of OPEs in Nepal indoor environments	OPEs	Yadav et al.^246^
indoor contamination comparison: Canada vs Czech Republic	PCBs, OCPs	Audy et al.^247^
indoor/outdoor PAH sources and exposure in Turkey	PAHs	Esen and Kayikci^248^
external exposure in Beijing	SCCPs, MCCPs	Gao et al.^249^
human exposure in Pakistan indoor air	PAHs	Hamid et al.^250^
human exposure in Australian indoor environments	OPEs, BFRs	He et al.^251^
tetrachlorinated PCB emissions from resin-coated cabinetry	PCBs	Herkert et al.^110^
effect of indoor airflow on PUF–PAS sampling accuracy	PCBs	Herkert and Hornbuckle^38^
sources in university laboratories in Hangzhou	PBDEs	Jin et al.^252^
PFAS characterization in indoor/outdoor air	PFAS	Karásková et al.^72^
flame retardants and plasticizers in Canadian homes measured by PDMS, XAD-coated PDMS and PUF samplers	FRs, plasticizers	Okeme et al.^111^
human exposure in occupational and home indoor settings	FTOHs, BFRs, OPEs, cVMSs	Sha et al.^169^
exposure in nonindustrial indoor air	PAHs	Villanueva et al.^253^
indoor air and dust exposure in Nepalese cities	NPACs, OPACs	Yadav et al.^254^
human exposure assessment in Beijing indoor air/dust	OPEs	Cao et al.^255^
indoor levels and human exposure	PCBs, PAHs, OCPs	Demirtepe et al.^176^
human exposure indoor air (different housing)	Consumer product chemicals	Dodson et al.^256^
indoor air and dust exposure in Ireland	PFAS	Harrad et al.^257^
inhalation risk from deodorant balls in Seoul public toilets	Naphthalene	Jung et al.^258^
indoor exposure in Australian environments	PAHs, PCBs, OCPs, CUPs	Wang et al.^37^
human exposure in Irish indoor air	BFRs	Wemken et al.^259^
exposure in Australian fire stations	PAHs, FRs	Banks et al.^260^
emerging & legacy POPs in European indoor air	OCPs, PCBs, PBDEs, DP	De la Torre et al.^220^
outdoor pesticide exposure at schools	CUPs	Gamboa et al.^261^
indoor/outdoor PAH exposure in mining communities	PAHs	Hendryx et al.^206^
indoor exposure in Beijing university campuses	Phthalates	Meng et al.^76^
human exposure in Bursa indoor air	PCBs	Sari et al.^262^
assessment of nondietary exposure among populations residing near neglected e-waste sites in Pakistan	PCNs	Waheed et al.^263^
indoor exposure sources in Bihar buildings	nBFRs, PBDEs, DP, OPEs	Yadav et al.^199^
exposure pathways in Canadian houses	HFRs	Yang et al.^215^
partitioning and exposure assessment	OPEs, PAHs	Yang et al.^264^
indoor exposure in Nigerian public bars	PCBs	Adesina et al.^202^
indoor exposure to environmental tobacco smoke in public bars in Nigeria	PAHs	Adesina et al.^265^
room-to-room variability of airborne PCBs in schools	PCBs	Bannavti et al.^200^
assessment of gull exposure at a landfill in the Montréal area (QC, Canada)	HFRs, OPEs	Kerric et al.^44^
indoor exposure at local public eateries in Western Nigeria	PAHs	Adesina et al.^211^
seasonal variation and exposure in classrooms/dormitories	phthalates	Duan et al.^266^
relationships between hair concentrations in humans and sheep and corresponding indoor and outdoor air levels	PAHs, PBDEs	Gevao et al.^267^
indoor/outdoor exposure comparison at coastal vs inland China	PAHs	Huang et al.^268^
indoor PAH emissions from domestic fuels in Nigeria	PAHs	Kehinde et al.^269^
indoor exposure in kitchens/living rooms in Beijing	OPEs	Lv et al.^270^
investigation of the impact of charcoal stoves on indoor PAH levels in Nigeria	PAHs	Okedere et al.^271^
exposure of Swedish seafarers onboard the ship	PAHs	Strandberg et al.^272^
congener-specific PCB emissions from floors/walls	PCBs	Bannavti et al.^217^
indoor/outdoor PAHs in Vietnamese vehicle workshops	PAHs	Hoang et al.^209^
risk assessment of exposure in rural greenhouses	CUPs	Hu et al.^273^
indoor PCB sources from glass-block windows	PCBs	Hua et al.^274^
indoor sources and exposure in rural South China	DDTs	Lv et al.^275^
indoor/outdoor exposure in Birmingham, UK	OPEs	Ortiz and Harrad^276^
occupational exposure at a CP production facility	SCCPs, MCCPs	Yu et al.^208^
indoor PCB exposure in beauty shops in Nigeria	PCBs	Atanda et al.^201^
risk assessment of human exposure in rural China	CUPs	Hu et al.^277^
indoor PAC levels in creosote-treated public buildings	PACs	Loive et al.^278^
multipathway exposure assessment in rural China	OPEs	Lv et al.^279^
indoor/outdoor concentrations in UK environments	BFRs	Ma et al.^280^
exposure in primary school children in Jinan, China	Phthalates	Yang et al.^281^

a Acronyms for all chemicals and other terms are defined in Table 6.

More recent research has expanded the analytical scope to include emerging chemicals, such as phthalates,^76^ OPEs,^169^ brominated flame retardants (BFRs),^213,214^ and plasticizers,^111^ which are emitted from electronic equipment,^215^ textiles,^216^ floorings,^217^ and personal care products.^218^ The introduction of SIP-PAS has further enhanced the capacity of passive sampling to capture more volatile and more polar and less hydrophobic compounds, including PFAS,^167^ FTOHs,^169^ and cyclic volatile methylsiloxanes (cVMSs),^169^ thus broadening the chemical applicability of passive samplers in indoor air research. PUF PAS-based studies also link airborne concentrations to human exposure, as shown for pesticides where air levels correlate with urinary metabolites in pregnant women.^219^

De la Torre et al.^220^ conducted an international study comparing residential indoor air in Belgium, Italy, Portugal, and Spain to monitor a broad range of legacy and emerging POPs. The project employed a unified passive sampling methodology to produce strictly comparable data across countries, enabling the identification of distinct national pollution patterns—for example, substantially lower ΣPCB and ΣDDX levels in Portugal and higher HCB levels in Spain—linked to differences in historical industrial activities and regulatory bans. This work fills a critical geographic data gap, representing the first documentation of these contaminants in homes in these countries.

PUF–PAS and SIP–PAS have proven highly effective for assessing indoor air quality by providing time-integrated measurements of the gas phase and, to a lesser extent, particle-associated SVOCs. Their extended sampling durations yield representative, long-term averages that more accurately reflect real-world human exposure compared with short-term active sampling. The widespread use of these samplers in indoor and occupational environments has generated critical insights into the persistence, emission dynamics, and exposure pathways of organic contaminants within modern built environments.

### 4.5 Emissions to Air from Source Sectors

PUF–PAS and SIP-PAS are highly effective tools for assessing both point and fugitive sources of POPs and emerging contaminants. These samplers provide time-integrated, spatially resolved measurements that can be used to (i) identify and map emission sources, (ii) quantify concentration gradients, and (iii) estimate atmospheric emissions.^164,282^ Their practicality and low maintenance requirements make them particularly suited for source characterization studies at local, regional, and even global scales.

The release of POPs and other emerging chemicals into the atmosphere arises from a variety of anthropogenic sources, including waste management^283^ and recycling facilities,^284^ industrial^161^ and agricultural activities,^285^ and secondary sources such as soils^177^ and surface waters.^286^ Among these, the waste sector—comprising wastewater treatment plants (WWTPs),^287^ landfills,^288,289^ e-waste recycling facilities,^290^ and tailings ponds,^33^ as well as industrial^291^ and combustion sources,^292^ remain major contributors to airborne semivolatile and particle-associated pollutants.^171,172,282^ Applications of PUF–PAS, SIP-PAS and PAS-DD for assessing such sources are summarized in Table 4.

**Table 4:** Application of PUF Based PAS for Assessing POPs and Emerging Chemicals of Concern in Air from Various Source Sectors

source type^a^	location	chemicals	references
Waste management and recycling sectors			
WWTP/municipal sewage treatment plant	Canada	SMCs	Wong et al.^170^
		PFAS, VMSs, organic UV filters	Shoeib et al.^172^
		PFCs	Vierke et al.^287^
	China	PFAS	Qiao et al.^310^
		PCBs, PBDEs	Wang et al.^283^
WWTP and landfills	Canada	PFCs	Ahrens et al.^307^
		VMSs	Cheng et al.^171^
e-waste	China	DP	Sun et al.^340^
		PAHs	Wei et al.^341^
		PBDEs	Xu et al.^342^
	Ghana	CPs	Arko et al.^299^
		PCBs	Hogarh et al.^290^
	India	PCDD/Fs, PCBs, PAEs, DEHA, PAHs	Chakraborty et al.^164^
	Pakistan	Heavy metals	Kazim et al.^32^
	Thailand	BFRs	Muenhor et al.^230^
landfill/waste dumping site	Canada	HFRs, OPEs	Kerric et al.^44^
	Chile	PAHs	Saa et al.^308^
	China	PFAS	Tian et al.^343^
	Ireland	PFAS, BFRs	Harrad et al.^288^
	Nigeria	PCBs	Omoruyi et al.^293^
	Pakistan	PCBs, PBDEs, DP	Hafeez et al.^344^
	Serbia	PAHs, PCBs, OCPs	Petrovic et al.^289^
	Turkey	PCBs, OCPs	Esen^102^
MSWI/medical waste incinerator	China	PBDD/Fs, PCDD/Fs	Li et al.^345^
		PCDD/Fs	Deng et al.^346^
		PCDD/Fs, PCBs	Gao et al.^347^
		PCDD/Fs, PCBs, PBDEs	Li et al.^114^
	Nigeria	Alkyl-naphthalenes	Adedayo Adesina et al.^294^
		PAHs	Adesina et al.^295^
	Spain	Metals and PCDD/Fs	Vilavert et al.,^296^ Vilavert et al.^297^
		PCDD/Fs	Domingo et al.^305^
		PCDD/Fs, PCBs, PCNs	Mari et al.^73^
			Vilavert et al.,^348^ Vilavert et al.^349^
	Poland	PCDD/Fs	Węgiel et al.^350^
landfill fire	Chile	PBDEs	Pozo et al.^304^
solid waste open burning	Nigeria	PAHs	Adesina et al.^292^
		PCBs	Adesina^306^
waste and plastic recycling	China	PBDEs, PAHs, PCBs	Qin et al.^302^
	Norway	PCBs	Arp et al.^301^
	Vietnam	942 organic micropollutants	Anh et al.^284^
end of life vehicle processing, shredding and metal recycling, ship breaking areas	Australia	PBDEs	Hearn et al.^351^
	Bangladesh	PAHs, SCCPs, DDTs, HCB, PCBs	Nøst et al.^352^
	Pakistan	PAHs, SCCPs, DDTs, HCB, PCBs, PBDEs	Shakoor Khan et al.^303^
	Vietnam	PAHs, Me-PAHs	Hoang et al.^209^
		PCBs, PBDEs, nBFRs, PAHs, Me-PAHs	Anh et al.^353^
former pesticide and herbicide sites	Pakistan	OCPs	Alamdar et al.^354^
	Spain	OCPs	Navarro et al.^158^
	Vietnam	PCDD/Fs, dl-PCB	Sau et al.,^330^ Sau,^329^ Sau^355^
Agricultural emission sources			
agricultural source	Argentina	endosulfan	Astoviza et al.^64^
	Austria	CUPs	Zaller et al.^285^
	Canada	CUPs	Messing et al.^356^
	Chile	CUPs	Cortes et al.^314^
		PAHs	Pozo et al.^357^
	China	DDT and its metabolites	Niu et al.^358^
		PCBs, PCNs, PCDD/Fs	Yang et al.^359^
	France	CUPs	Martin et al.,^315^ Angelier et al.^316^
	India	Dicofol	Eng et al.^155^
		OCPs, PCBs	Pozo et al.^312^
	South Africa	OCPs, CUPs	Veludo et al.^313^
	Turkey	OCPs, PCBs	Can-Güven et al.,^113^ Can-Güven et al.^311^
Industrial emission sources			
chemical production plants	China	CPs	Li et al.,^317^ Yu et al.^208^
		DP	Zhang et al.^360^
		PFAS	Chen et al.^361^
coal power plant	India	PAHs	Gune et al.^320^
fluoropolymer manufacturing park	China	PFAS	Liu et al.^362^
metal production industry (iron, steel, copper, aluminum)	China	PCDD/Fs, PCBs, PBDEs	Li et al.^318^
		PCDD/Fs, dl-PCB, PCNs	Hu et al.^323^
	South Korea	PCBs, PBDEs	Choi et al.^363^
abandoned Zn–Pb–Cu mine, gold mine	Canada	fugitive tailings dust transport and deposition	Cleaver et al.^33^
		mass, trace elements	Berryman et al.^31^
oil/gas production; petrochemical area	Brazil	PACs	Euzebio et al.^319^
	China	PACs	Liu et al.,^29^ Zhang et al.^364^
	Spain	HPVCs, PACs	García-Garcinuño et al.^119^
		PAHs	Domínguez-Morueco et al.^365^
oil sands and mining	Canada	PACs	Harner et al.,^57^ Schuster et al.,^282^ Jariyasopit et al.,^28^ Chibwe et al.,^324^ Schuster et al.,^321^ Jariyasopit et al.,^325^ Vasiljevic et al.,^328^ Moradi et al.^327^
		heterocyclic aromatics	Manzano et al.^326^
industrial complex	South Korea	PCBs, PCNs, PBDEs	Baek et al.^161^
		PACs	Nguyen et al.,^366^ Vuong et al.^367^
	China	PCDD/Fs	Ren et al.^368^
		PCDD/Fs, PCBs, PCNs	Hu et al.^322^
		PACs	Wang et al.^369^
	Argentina	PAHs	Wannaz et al.^370^
	Turkey	PAHs, PCBs	Aydin et al.,^371^ Odabasi et al.,^372^ Cetin et al.^373^
		PAHs	Sanli et al.^374^
		PBDEs	Cetin et al.^375^
		OPEs, plasticizers	Kurt-Karakus et al.^376^
	Colombia	PCDD/Fs, dl-PCB	Cortés et al.^291^
source sectors	Canada	OPEs, PBDEs, nFRs	Saini et al.^377^
Secondary emission sources (water and soils)			
water/seawater/river	Aegean Sea	PACs, PCBs, OCPs, PBDEs	Lammel et al.^286^
	China	OPEs	Wang et al.^334^
		PACs	Wu et al.^378^
		PCBs, PBDEs	Wang et al.^283^
	Himalayan glaciers	PACs, PCBs	Sharma et al.^332^
	India	OCPs	Khuman and Chakraborty^379^
		PAEs	Chandra and Chakraborty^380^
	Turkey	PCBs	Sari et al.^331^
	US	PCBs	Martinez et al.,^335^ Martinez et al.^182^
soil	China	OCPs	Qu et al.,^180^ Wang et al.^67^
		OCPs, PACs	Wang et al.^381^
		OPEs	Wang et al.^382^
		PACs	Wu et al.,^383^ Wang et al.,^384^ Wang et al.,^382^ Wu et al.,^385^ Hu et al.,^386^ Qi et al.,^178^ Zhang et al.,^387^ Wang et al.^388^
		PCNs	Die et al.^389^
	Central and Southern Europe	OCPs, PCBs	RůŽičková et al.^390^
	Pakistan	CPs	Tahir et al.^309^
		OCPs	Syed et al.,^391^ Alamdar et al.,^354^ Bajwa et al.^392^
		OCPs, PCBs	Sohail et al.^177^
		PCBs	Syed et al.,^393^ Eker Sanli and Tasdemir^394^
	Peru, Turkey	PAHs, PCBs, OCPs	Esen et al.^333^
	Turkey	OCPs, PCBs	Can-Güven et al.^311^
		PAHs, PCBs	Kaya et al.,^395^ Cetin et al.,^396^ Dumanoglu et al.^397^
		PCBs	Eker Sanli et al.^398^
	Global	PCBs	Li et al.^399^
	UK	PCBs	Desborough and Harrad^100^
Others			
biomass/wood burning; forest fire	Chile	PAHs	Pozo et al.^400^
	China	Monosaccharides	Jiang et al.^74^
	Indo-China	PCBs, OCPs	Jiang et al.^401^
		Monosaccharides	Jiang et al.^402^
charcoal stove	Nigeria	PAHs	Okedere et al.^271^
diesel-fueled generators	Nigeria	PAHs	Oyelami et al.^337^
lake	Norway	PBDEs	Mariussen et al.^403^
old house (joint sealants)	Switzerland	PCBs	Diefenbacher et al.^404^
plastic greenhouse and conventional cultivation methods	China	OCPs	Zhang et al.^405^
traffic/exhaust gas	Norway	PACs	Klingberg et al.^336^
	Sweden	metals	Pleijel et al.^339^
	Turkey	PAHs	Cihangir et al.^406^

a Acronyms for all chemicals and other terms are defined in Table 6.

#### 4.5.1 Waste Management and Recycling Sectors

PUF–PAS has been widely applied to characterize emissions from waste management, recycling, and disposal facilities, which are major sources of both legacy and emerging organic pollutants. Studies around landfills,^293^ incinerators,^294^ e-waste dismantling sites,^164^ and WWTPs^170^ have reported elevated concentrations of PCBs,^102^ PBDEs,^283^ PAHs,^295^ PCDD/Fs,^296,297^ phthalates,^298^ OPEs,^44^ and CPs,^299^ reflecting ongoing volatilization from contaminated materials and thermal processes.

Shoeib et al.^172^ used SIP-PAS around WWTPs and found that emissions of PFAS, UV filters, and cyclic siloxanes were significantly higher in summer than in winter, and concentrations were up to 35 times higher at WWTPs compared to background sites.

Harrad et al.^288^ analyzed air and soil samples collected between November 2018 and January 2019 from locations upwind and downwind of 10 landfills across the Republic of Ireland. Concentrations of PFAS, PBDEs, HBCDD, and DBDPE did not differ significantly between upwind and downwind sites (*p* > 0.05), suggesting that these landfills had no detectable effect on local air quality for the measured contaminants.

E-waste is one of the fastest-growing solid waste streams worldwide. In 2022, an estimated 62 million tonnes of e-waste were generated globally, yet only 22.3% was formally collected and recycled (https://www.who.int/news-room/fact-sheets/detail/electronic-waste-(e-waste)). A substantial portion of this waste is exported from developed countries to developing nations, raising concerns about environmental and human health impacts in regions lacking a proper recycling infrastructure. In addition to its growing volume, e-waste represents an important source of atmospheric emissions and is increasing at a rate 3 times faster than the world’s population.^300^ In India, Chakraborty et al.^164^ conducted intensive PUF–PAS sampling in four Indian megacities along five transects: e-waste, information technology, industrial, residential, and dumpsites. Concentrations of ∑_17_PCDD/Fs, ∑_25_PCBs, ∑_7_ plasticizers, and ∑_15_PAHs ranged from 3.1–26 pg/m^3^, 0.5–52 ng/m^3^, 7.5–520 ng/m^3^, and 6–33 ng/m^3^, respectively. E-waste recycling contributed 45% of total PCBs and was identified as a major factor with dl-PCBs, particularly PCB-126, indicating combustion as a primary source. PCDD/Fs, PCBs, and plasticizers were highest at e-waste sites, while PAHs peaked in industrial areas, followed by e-waste sites. Marker plasticizers dibutyl phthalate (DBP) and bis-2-ethylhexyl adipate (DEHA) were significantly elevated in Bangalore compared to other cities.

At e-waste recycling sites, PUF–PAS has detected high levels of HFRs and PCBs,^230,290,301,302^ confirming that dismantling,^303^ open burning,^304^ and informal recycling contribute substantially to local and regional air contamination. Around waste incinerators, PAHs,^295^ PCDD/Fs^305^ and PCBs^306^ are often detected in the near field, highlighting the role of incomplete combustion and ash handling as diffuse emission pathways. Likewise, landfill cover soils and leachate ponds act as continuous secondary sources through volatilization and fugitive gas exchange,^307,308^ particularly under warm and dry conditions.

In addition to detecting pollutant mixtures, PUF–PAS has been instrumental in mapping spatial gradients and identifying emission “hotspots” around waste-handling facilities.^309^ By integrating concentration data with the prevailing meteorological conditions, researchers have used PUF–PAS to estimate emission fluxes and source contributions to surrounding communities. In urban-industrial regions, PUF-based data have revealed that waste management facilities often rank among the dominant contributors to local POP burdens.^164^ Moreover, the method’s simplicity enables deployment at multiple heights or distances, helping evaluate the effectiveness of emission control technologies or site remediation efforts. The use of PUF–PAS in these sectors continues to expand toward monitoring emerging contaminants such as nFRs, plasticizers, and PFAS,^172,310^ supporting risk assessment and policy development.

#### 4.5.2 Agricultural Emission Sources

In agricultural regions, passive samplers have been used to assess emissions associated with pesticide application. Studies of open-field burning and crop residue combustion have detected elevated concentrations of metals and pesticide residues, including OCPs, and CUPs.^64,85,311–313^

In the agricultural sector, PUF–PAS has become a standard tool for quantifying airborne pesticides and their transformation products, bridging the gap between emission events and exposure assessment. Studies in India,^312^ Turkey,^113,311^ Chile,^314^ South Africa,^313^ and Europe^315,316^ have shown that concentrations of OCPs and CUPs in air peak during spraying seasons and are influenced by temperature, land use, and application intensity.^313^ In vineyards^315^ and greenhouse areas,^311^ PUF–PAS detected extremely high levels of fungicides such as tebuconazole, chlorpyrifos, and metalaxyl, with spatial variations closely aligned with field activities. Agricultural monitoring using PUF–PAS has also provided valuable data for health risk assessment, revealing potential inhalation risks, particularly for children and workers in pesticide-intensive regions. In addition, the long-term data sets generated by these samplers enable the evaluation of temporal trends, including the decline of legacy compounds and the emergence of new pesticide formulations.

Zaller et al.^285^ demonstrated that airborne pesticide residues from agricultural activities present considerable risks to both the environment and human health. Passive air sampling across 15 regions in eastern Austria detected 67 active ingredients—including herbicides, fungicides, and insecticides—with higher concentrations in areas with extensive arable land. Frequently detected compounds included prosulfocarb, folpet, glyphosate, and chlorpyrifosethyl, and their distribution was influenced by seasonal changes, temperature, radiation, and wind patterns. Contamination was also observed in protected areas and urban centers.

#### 4.5.3 Industrial Emission Sources

In industrial environments, PUF-based PAS has been applied for monitoring diffuse and stack emissions from chemical production plants,^208,317^ metal smelters,^318^ oil and gas operations,^319^ and coal combustion facilities.^320^ These studies have measured atmospheric concentrations of PCBs, PAHs, CPs, and dioxins, showing that emissions are influenced by the process type, production volume, and combustion efficiency. PUF–PAS deployments around oil sands operations, petrochemical complexes, and mining regions have revealed elevated levels of alkylated PAHs,^321^ petroleum-derived hydrocarbons, and metal-associated organics, supporting its value in quantifying near-field impacts and long-range transport potential. The passive nature of PUF samplers allows for deployment in remote or inaccessible industrial zones where continuous or high-volume sampling may not be feasible. Furthermore, integrating PUF–PAS data with source-receptor and trajectory models enhances the understanding of regional pollutant dispersion and deposition patterns.

In Henan Province, China, 60 PUF–PAS were deployed across five cities with varying industrial profiles to assess the impact of CPs-related industries, including CP manufacturing, metalworking, and polyvinyl chloride (PVC) production for identifying sector-specific emission patterns.^317^ Widespread contamination by SCCPs and MCCPs was observed, with Luoyang showing the highest MCCP levels, reflecting its strong metalworking sector. Statistical and clustering analyses indicated that the industrial structure strongly influences CP profiles, with metalworking contributing more to MCCP emissions than PVC or CP manufacturing.

PUF–PAS has been widely applied for assessing industrial emissions of unintentionally produced POPs (UP-POPs), particularly in regions with intensive metallurgical and thermal processing activities. Hu et al.^322^ deployed passive samplers at an industrial park located at the northeastern edge of the Tibet–Qinghai Plateau to measure ambient concentrations of PCDD/Fs, PCNs, and dl-PCBs. Concentrations of PCDD/Fs (1.18–2.18 pg/m^3^), PCNs (21.9–75.1 pg/m^3^), and dl-PCBs (0.49–0.90 pg/m^3^) were substantially higher than those measured at a remote background site and comparable to levels reported in other industrialized regions. Principal component analysis identified emissions from local thermal processes, including a secondary aluminum smelter, a cement kiln, and a lead–zinc smelter, as major contributors. The combustion-related PCN congener profiles further demonstrated the influence of industrial thermal activities on local air composition. In a related study, Hu et al.^323^ employed PUF–PAS around five secondary nonferrous metal processing facilities across China to evaluate the dispersion and environmental impact of UP-POPs. Downwind concentrations of PCDD/Fs, dl-PCBs, and PCNs (4.70–178, 8.23–7520, and 152–4190 pg/m^3^, respectively) were notably higher than those observed upwind, indicating direct contributions from plant emissions. Strong correlations between ambient air and stack gas concentrations, along with similar homologue and congener patterns, confirmed that these metallurgical operations were major local emission sources.

A series of studies^28,282,321,324–328^ have applied PUF-based PAS to investigate industrial emissions from multiple sources and atmospheric processes of PACs and their derivatives in the Athabasca Oil Sands Region, Alberta, Canada. PUF–PAS and PAS-DD deployed along a 90 km transect from Fort McMurray to the northern mining area (Oct–Nov 2015) revealed that ΣPAC and nitrated polycyclic aromatic compounds (NPACs) concentrations were highest near mining and upgrading sites, while oxygenated polycyclic aromatic compounds were elevated in Fort McMurray. Source material analysis showed that oil sands ore contained up to 40× higher ΣPACs than petroleum coke (petcoke), implicating ore dust as a major contributor, with scanning electron microscopy coupled with energy dispersive X-ray spectroscopy (SEM-EDS) confirming the presence of petcoke particles in passive samples. Long-term monitoring from 2010 to 2016 at 15–17 sites indicated total PACs, total alkylated polycyclic aromatic compounds (Alk-PACs), and total dibenzothiophene and its alkylated derivatives (DBTs) concentrations of 0.3–43, 0.15–460, and 0.04–130 ng/m^3^, respectively, with levels decreasing exponentially with distance from open-pit mines. Temporal trends showed minor increases over time, while seasonal patterns were pronounced for more volatile PACs, peaking in the winter. Forest fires in 2011 and 2015 produced short-term spikes in PAHs but had little effect on Alk-PACs or DBTs, confirming their association with petrogenic sources. Additional analyses of NPACs in snow, sediments, and air identified over 200 compounds, with profiles closely matching that of petcoke, confirming it as a dominant NPAC source and transport vector. Some NPACs were detected more than 100 km from mining activities, demonstrating regional atmospheric dispersion. PUF–PAS mapping around a major tailings pond showed ΣPAHs, ΣAlk-PAHs, and ΣDBTs ranging from 13–70, 220–970, and 30–210 ng/m^3^, respectively, with higher concentrations in downwind samplers, highlighting the pond as a significant local PAC source.

Passive samplers have also been instrumental in monitoring remediation and contaminated legacy sites, where volatilization or disturbance can reintroduce pollutants into the atmosphere. At dioxin-contaminated soils^329,330^ and historical lindane production areas, passive measurements have provided valuable evidence of ongoing emissions, even decades after site closure or remediation efforts.

#### 4.5.4 Secondary Emission Sources: Soils and Surface Waters

In addition to primary emissions, PUF–PAS has been instrumental in elucidating air–surface exchange processes, which act as secondary sources of atmospheric pollutants. Studies have demonstrated that contaminated and agricultural soils re-emit legacy OCPs, PCBs, and PAHs to the atmosphere through temperature-dependent volatilization, with fluxes peaking during warm months. Seasonal patterns indicate a dynamic air–soil exchange governed by ambient temperature, soil organic content, and compound volatility. In Lahore, Pakistan, Tahir et al.^309^ reported spatial variation and dynamic air–soil exchange of SCCPs and MCCPs, illustrating the importance of secondary emissions in sustaining regional atmospheric contamination. Similarly, PUF–PAS studies have examined air–plant and air–soil interactions in large reservoirs and agricultural regions, revealing bidirectional exchange of PAHs and other SVOCs in response to temperature and land-use conditions.^309^

Similarly, PUF–PAS has provided evidence that water bodies and sediments are significant secondary sources of POPs.^331^ Deployments near rivers, lakes, and coastal waters have measured high volatilization fluxes of PCBs,^332^ PAHs,^333^ and OPEs,^334^ especially during warm seasons and in areas affected by industrial or urban runoff. Case studies at historically contaminated sites such as harbors^182,335^ and reservoirs show that long-deposited pollutants continue to exchange with the atmosphere decades after direct inputs ceased. The passive samplers also capture re-emissions driven by glacial meltwater and sediment resuspension, revealing the effects of climate change on pollutant remobilization. For example, melting Himalayan glaciers contaminated by legacy atmospheric deposits have been identified as significant sources of PCBs and high-molecular-weight PAHs to the Ganges floodplain during dry periods.^332^ These findings confirm that soils and surface waters serve as persistent, temperature-sensitive secondary sources that sustain global atmospheric cycling of legacy and emerging contaminants. PUF–PAS thus provides an invaluable means to quantify revolatilization processes, assess recontamination potentials, and inform long-term environmental management strategies.

#### 4.5.5 Other Emission Sources

Beyond large-scale sources, PUF-based PAS has been applied to characterize emissions from small, localized sources such as traffic/exhaust gas,^336^ charcoal stove,^271^ and diesel-fueled generators.^337^ Passive samplers deployed along major traffic corridors capture pollutants from both vehicle exhaust and nonexhaust sources like brake and tire wear.^175,338^ These studies have documented elevated levels of metals^339^ and PAHs^336^ from PUF–PAS in roadside environments and provided insight into pollutant uptake by vegetation. For instance, accumulation of metals has been observed in the leaves of *Quercus palustris* and the needles of *Picea abies* and *Pinus sylvestris*, demonstrating the strong influence of traffic emissions on plant.^339^ Moreover, studies using *Cucurbita pepo* have shown that exposure to exhaust gases not only results in pollutant accumulation but also causes visible physiological damage to the plants, emphasizing the detrimental effects of vehicular emissions on vegetation and potential implications for human health through the food chain.^336^

### 4.6 Health Studies: Indicators of Toxicity Using PUF–PAS

Several studies have employed PUF–PAS extracts to investigate inhalation-related health risks and ecological toxicity by linking ambient chemical exposures to toxicological endpoints. Table 5 summarizes published research that has applied PUF–PAS to toxicological evaluation. In this approach, the PUF–PAS serves as a surrogate lung by filtering and accumulating gases and particles in air, approximately 4 m^3^/day, which is similar to the daily inhalation volume for an infant.^407^ This makes PUF–PAS a scientifically relevant proxy for assessing real-world exposures that are relevant to both humans and wildlife.

**Table 5:** Applications of PUF–PAS and SIP-PAS in Toxicological Evaluation, Quantitative Cancer Risk Assessment, and Related Health Studies

application/study^a^	assay (or sample) type	reference
alkaline phosphatase response to PAH toxicity	SOS chromotest	Čupr et al.^411^
estrogenic and AhR-mediated activity from PAHs	E-SCREEN and CAFLUX assay	Kennedy et al.^412^
genotoxic potency and AhR activity of PAHs	umuC genotoxicity assay, CAFLUX bioassay	Kennedy et al.^413^
AhR activity, (*anti*-)estrogenicity and (*anti*-)androgenicity of PAHs	H4IIE-*luc* bioassay	Érseková et al.^415^
AhR activity of airborne PCBs and PCDD/Fs	CAFLUX bioassay	Chakraborty et al.^425^
direct/indirect mutagenicity and cytotoxicity of PACs	Salmonella mutation assay and lactate dehydrogenase assay	Jariyasopit et al.^414^
gene expression effects and cytotoxicity of OFRs	Avian *in vitro* cytotoxicity screening	Ha et al.^426^
acute pulmonary effects to urban-air-derived PACs	*In vitro* cytotoxicity, pro-inflammatory, oxidative stress	Halappanavar et al.^410^
carcinogenic potential and AhR activity of airborne PBDD/Fs and PCDD/Fs	Yeast two-hybrid assay	Li et al.^345^
PAH bioaccessibility in lung epithelial cells	PAH exposure to lung epithelial cell lines in culture medium	Shahpoury et al.^427^
Other health/exposure studies		
integrated assessment of PBDE exposure using matched samples of human milk, indoor air, and dust		Toms et al.^229^
PAH air levels, inhalation exposure, and incremental lifetime cancer risk		Xia et al.^417^
cancer risk from inhalation of OCPs and PCBs		Zhang et al.^418^
dietary intake, distribution, and screening-level PCN risk assessment in cereals, soils, and air		Mahmood et al.^420^
dietary intake, distribution, and screening-level PCB risk assessment in cereals, soils, and air		Mahmood et al.^419^
PCB congener-specific inhalation and dietary exposure in adolescents and mothers		Ampleman et al.^232^
health risk assessment of outdoor PAH exposure, including BaP-equivalent levels and attributable lung cancer risk		Hong et al.^421^
DNA repair and apoptosis-related gene expression in birds exposed to high airborne PACs		Wallace et al.^416^
risk assessment based on PCB exposure from ambient air estimates associated health risks, identifying the thyroid as a primary target organ for PCB toxicity in the community		Heiger-Bernays et al.^424^
health risk assessment of PCNs using indoor air, dust, and serum		Waheed et al.^263^
comparison of pesticide levels in air with urinary metabolites among pregnant women		Giffin et al.^219^
human exposure to atmospheric PAHs in an agricultural region of central Chile and associated inhalation cancer risks		Pozo et al.^357^
toxicity-equivalent concentrations and incremental lifetime cancer risk (PAHs)		Ukpebor et al.^422^
occurrence and environmental behavior of DP in soil, air, and human serum from electronic waste dismantling areas to understand its distribution and potential health implications		Dalanggud et al.^423^
OPE exposure assessment via dietary and nondietary pathways		Lv et al.^279^
health risk assessment including toxicity-equivalent concentrations and incremental lifetime cancer risk (PAHs)		Ma et al.^428^

a Acronyms for all chemicals and other terms are defined in Table 6.

**Table 6:** Abbreviations and Their Full Names Used in This Review

abbreviation	full name
AAS	active air sampling
AhR	aryl hydrocarbon receptor
Alk-PACs	alkylated polycyclic aromatic compounds
AZE	azinphos ethyl
BaP	benzo[*a*]pyrene
BFRs	brominated flame retardants
CAFLUX	chemically activated fluorescent gene expression
CEE	Central and Eastern Europe
CFD	computational fluid dynamics
COSMO-RS	conductor-like screening model for real solvents
CPFM	chlorpyrifos-methyl
CPs	chlorinated paraffins
CUPs	current-use pesticides
cVMSs	cyclic volatile methylsiloxanes
DBP	dibutyl phthalate
DBTs	dibenzothiophenes
DCs	depuration compounds
DDT	dichloro-diphenyl-trichloroethane
DEHA	bis-2-ethylhexyl adipate
dl-PCBs	dioxin-like polychlorinated biphenyls
DP	dechlorane plus
EC	elemental carbon
ECCC	Environment and Climate Change Canada
eDNA	environmental DNA
EMEP	European Monitoring and Evaluation Programme
e-waste	electronic waste
FRs	flame retardants
FTOHs	fluorotelomer alcohols
GAPS	Global Atmospheric Passive Sampling
GEF	Global Environment Facility
GFF	Glass Fiber Filter
GLB	Great Lakes Basin
GMP	Global Monitoring Plan
GRULAC	Group of Latin America and Caribbean Countries
HBCDD	hexabromocyclododecane
HCB	hexachlorobenzene
HCH	hexachlorocyclohexane
HFRs	halogenated flame retardants
HOCs	halogenated organic contaminants
HPVCs	high production volume chemicals
IADN	Integrated Atmospheric Deposition Network
ISRP	Iowa Superfund Research Program
*K*_OA_	octanol–air partition coefficient
*K*_PUF‑A_	PUF–air partition coefficient
*K*_SIP‑A_	SIP–air partition coefficient
LCCPs	long-chain chlorinated paraffins
lVMSs	linear volatile methylsiloxanes
MCCAs	medium-chain chlorinated *n*-alkanes
MCCPs	medium-chain chlorinated paraffins
Me-PAHs	methylated polycyclic aromatic hydrocarbons
MONET	MOnitoring NETwork
MSWI	municipal solid waste incinerator
nBFRs	novel brominated flame retardants
NCP	Northern Contaminants Program
nFRs	novel flame retardants
NPACs	nitrated polycyclic aromatic compounds
OCPs	organochlorine pesticides
OFRs	organic flame retardant
OH-PCBs	hydroxylated polychlorinated biphenyls
OPACs	oxygenated polycyclic aromatic compounds
OPEs	organophosphate esters
PACs	polycyclic aromatic compounds
PAEs	phthalic acid esters
PAHs	polycyclic aromatic hydrocarbons
PAS	passive air sampler
PAS-DD	passive dry deposition sampler
PAWS	personal air wearable sampler
PBBz	polybromobenzenes
PBDD/Fs	polybrominated dibenzo-*p*-dioxins and furans
PBDEs	polybrominated diphenyl ethers
PCBs	polychlorinated biphenyls
PCDD/Fs	polychlorinated dibenzo-p-dioxins and polychlorinated dibenzofurans
PCMs	polycyclic musks
PCNs	polychlorinated naphthalenes
PeCA	pentachloroanisole
PeCB	pentachlorobenzene
PFAAs	perfluoroalkyl acids
PFAS	per- and polyfluoroalkyl substances
PFCs	poly- and perfluorinated compounds
PFOA	perfluorooctanoic acid
PFOS	perfluorooctanesulfonate
POPs	persistent organic pollutants
pp-LFER	poly parameter linear free rnergy relationship
PUF	polyurethane foam
PUF–PAS	polyurethane foam -passive air sampler
PVC	polyvinyl chloride
QA/QC	quality assurance/quality control
QSPR	quantitative structure–property relationship
QSPRs-ANN	quantitative structure–property relationships-artificial neural network
QSPRs-MLR	quantitative structure–property relationships-multiple linear regression
QSPRs-SVM	quantitative structure–property relationships -support vector machine
RECETOX	Research Centre for Toxic Compounds in the Environment
*R*_s_	sampling rate
SCCAs	short-chain chlorinated *n*-alkanes
SCCPs	short-chain chlorinated paraffins
SEM-EDS	scanning electron microscopy coupled with energy dispersive X-ray spectroscopy
SIP	sorbent-impregnated PUF
SIP-PAS	sorbent-impregnated polyurethane foam -passive air sampler
SMCs	synthetic musk compounds
SMP	Spanish monitoring program
SVOCs	semi-volatile organic compounds
TBBP-A	tetrabromobisphenol-A
UNEP	United Nations Environment Programme
UP-POPs	unintentionally produced POPs
vHOPs	volatile hydrophobic organic pollutants
VMSs	volatile methyl siloxanes
WEOG	Western Europe and Others Group
WWTPs	Wastewater Treatment Plants
XAD	styrene–divinylbenzene copolymer

PUF–PAS are well suited for this application since they provide time-weighted average concentrations of air contaminants representing both gas-phase chemicals and particle-associated chemicals present in ambient air. Furthermore, varying source sectors (both indoors and outdoors) can be easily targeted using PAS due to its ease of deployment. The findings from these studies help identify areas and source sectors with elevated pollutant levels, as they are commonly linked to increased health risk factors,^408–410^ and the findings also contribute valuable information to public health and wildlife protection for mitigating exposure to airborne contaminants.

Toxicological assessment using PUF–PAS extracts has been extensively demonstrated. The pioneering study by Čupr et al.^411^ applied the SOS Chromotest using *Escherichia coli* sulA::lacZ to evaluate genotoxicity of ambient air extracts, revealing dose-dependent responses that correlated with PAH concentrations in 2006. In 2009, Kennedy et al.^412^ applied E-SCREEN and chemically activated fluorescent gene expression (CAFLUX) bioassays to quantify estrogenicity and aryl hydrocarbon receptor (AhR)-mediated activity from PAHs, respectively. Results showed that estrogenic potency measurements were highest in indoor offices, followed by indoor suburban homes, whereas outdoor air of suburban homes was not estrogenic. In contrast, the greatest AhR activity was for outdoor air in suburban homes. Subsequent work by Kennedy et al.^413^ further applied the umuC assay and CAFLUX assay to examine genotoxicity and AhR activity from PAHs, respectively, demonstrating elevated genotoxicity and AhR activity during winter at urban sites. More recently, Jariyasopit et al.^414^ employed PUF–PAS extracts from the Athabasca Oil Sands Region to evaluate the application of *in vitro* assays for mutagenicity and cytotoxicity, while Halappanavar et al.^410^ employed *in vitro* cytotoxicity, pro-inflammatory, and oxidative stress assays to assess acute pulmonary effects, identifying the spring season in Toronto as exhibiting the greatest biological potency.

Coupling PAS with bioanalytical assays enables evaluation of genotoxicity, cytotoxicity, and receptor-mediated responses such as AhR, estrogenic, and *anti*-androgenic activity, revealing that both quantified and nontarget compounds contribute to observed biological effects.^412,413,415,416^ Overall, these studies highlight PAS as a versatile tool for monitoring pollutant levels, evaluating potential biological impacts, and forming risk assessments in both human and ecological contexts.

A small but growing body of research has leveraged PUF–PAS–derived concentrations to perform quantitative human health risk assessments and to evaluate linkages between ambient POP levels and internal human exposure. Several studies^357,417–422^ have incorporated modeled air concentrations into cancer and noncancer risk frameworks, while others have integrated PAS measurements with human biomonitoring data—including breast milk,^229^ urine,^219^ and serum^423^—to assess exposure pathways and dose–response relationships.

Heiger-Bernays et al.^424^ conducted a risk assessment using PCB concentrations estimated from ambient PAS measurements and demonstrated that inhalation exposure contributed meaningfully to total PCB body burden, with the thyroid emerging as a key target organ of toxicological concern. Giffin et al.^219^ examined pesticide exposure in pregnant women in Costa Rica and reported significant associations between air concentrations measured via PUF–PAS and urinary metabolite levels, indicating the relevance of inhalation as an exposure route during pregnancy. Toms et al.^229^ performed an integrated PBDE exposure assessment by combining matched measurements of human milk, indoor air, and dust, providing evidence that multimedia integration—supported partly by PAS measurements—can more accurately characterize cumulative exposure in vulnerable populations.

Collectively, these studies illustrate the potential of PAS data to support human health evaluations, bridge exposure measurements across environmental and biological matrices, and strengthen the mechanistic understanding of inhalation-driven SVOC uptake.

## 5 Future Directions

The PUF and SIP disk samplers have made a tremendous impact in the field of atmospheric research and monitoring of contaminants in air for more than 25 years. The sampler’s compact design, low cost, and ability to operate without the use of electricity make it suitable to be shipped for use in remote or international locations. Compilations of monitoring data generated by PUF and SIP disk samplers (e.g., Schuster et al.,^14^ White et al.^193^ and UNEP^25^) show that despite the incredible amount of new information on chemicals in the air which has been made possible by these samplers, there are still many data gaps to address related to growing demands for monitoring and research of contaminants and emerging chemicals in the air. Some researchers warn that the planetary boundary (safe operating space) has been exceeded for novel entities, which includes new chemicals.^429^ Routine intercalibration exercises are also needed to inform on data comparability among groups and for the growing number of target chemicals.^23^

In addition to targeted measurements on chemicals and chemical classes, which have been done so far, there is increasing demand for a more complete scan and holistic assessment of the full range of chemicals in air (chemical mixtures), including transformation products of parent contaminants.^19^ This research need can be facilitated by PUF and SIP disk samplers because they capture the full range of gas- and particle-associated contaminants from ambient air. There are also many recognized incentives to link air measurements of contaminants with other cross-cutting issues such as health, climate change, and biodiversity (loss) (e.g., by tracking environmental DNA (e-DNA) in air), which is also facilitated by the simplicity of PUF and SIP disk samplers.^26,430,431^ Enhanced collaboration across science disciplines and policy is encouraged to realize and evolve the full scope of PUF and SIP disk samplers for the next generation of research challenges.

## Supplementary Material



## Notes

Compilation of ambient air research conducted from 2004 to 2024 using PUF-based passive air samplers (PDF)
