# Supplementary material for: Twenty-Five Years of Research and Monitoring Using PUF Disk Passive Air Samplers
Source: Environ Sci Technol. 2026 Apr 16;60(16):11833–70. doi: 10.1021/acs.est.5c17602 (PMC13130972; doi:10.1021/acs.est.5c17602)
Supplement: Supplementary file 1 [file es5c17602_si_001.pdf]

Twenty-five Years of Research and Monitoring using PUF Disk Passive Air Samplers

Anita Eng†, Yu-Mei Hsu†, Tom Harner*, Jacob Mastin, and Samantha Wheadon

Air Quality Processes Research Section, Environment and Climate Change Canada, Toronto, Ontario, M3H 5T4, Canada

† **Co-First Authors**

*** Corresponding Author**

Tom Harner - Air Quality Processes Research Section, Environment and Climate Change Canada, 4905 Dufferin St., Toronto, Ontario, M3H 5T4, Canada

Email: Tom.Harner@ec.gc.ca

Supporting Information

Table S1. Ambient air research studies and monitoring programs employing PUF-PAS, SIP-PAS or PAS-DD from 2004 to 2024[1]

UN Region†	PCBs	FRs	OCPs	CUPs	PAH/PACs	UV Blockers/ Antioxidants	PCDD/Fs	PFAS	Siloxanes	Trace metals	Others	Reference
**2004**												
WEOG	×		×									Harner et al. (2004)
WEOG					×						PCNs	Jaward et al. (2004b)
WEOG	×	×	×									Jaward et al. (2004a)
GRULAC	×	×	×									Pozo et al. (2004)
**2005**												
WEOG											SCCAs, MCCAs	Barber et al. (2005)
WEOG	×	×	×									Gouin et al. (2005)
WEOG	×		×									Jaward et al. (2005a)
Asia/Pacific	×	×	×									Jaward et al. (2005b)
WEOG	×		×		×							Motelay-Massei et al. (2005)
**2006**												
Asia/Pacific		×			×							Gevao et al. (2006)
Global/multi-region			×									Harner et al. (2006a)
WEOG		×										Harner et al. (2006b)
WEOG											PCNs	Harner et al. (2006c)
WEOG		×										Harrad and Hunter (2006)
CEE	×		×		×							Klánová et al. (2006)
Global/multi-region	×	×	×									Pozo et al. (2006)
**2007**												

[1] Abbreviations and their full names are listed in Table 5.

† WEOG: Western European and Others Group; GRULAC: Group of Latin America and Caribbean Countries; CEE: Central and Eastern Europe.

UN Region†	PCBs	FRs	OCPs	CUPs	PAH/PACs	UV Blockers/ Antioxidants	PCDD/Fs	PFAS	Siloxanes	Trace metals	Others	Reference
WEOG	×	×	×		×							Gioia et al. (2007)
CEE	×											Klánová et al. (2007)
Global/multi-region											PCNs	Lee et al. (2007)
Asia/Pacific					×							Liu et al. (2007)
WEOG			×									Moreau-Guigon et al. (2007)
CEE	×	×	×		×						PCNs	Roots and Sweetman (2007)
Asia/Pacific					×							Santiago and Cayetano (2007)
Asia/Pacific					×							Tao et al. (2007)
Asia/Pacific			×									Wang et al. (2007)
WEOG				×								Yao et al. (2007)
CEE					×							Zencak et al. (2007)
**2008**												
GRULAC	×		×									Alegria et al. (2008)
GRULAC												Estellano et al. (2008)
WEOG				×								Gouin et al. (2008)
Asia/Pacific												Liu et al. (2008)
CEE	×		×		×							Milukaitė et al. (2008)
Asia/Pacific		×										Ren et al. (2008)
Asia/Pacific	×										EOCl/Br/I	Xu et al. (2008)
Asia/Pacific	×	×	×									Zhang et al. (2008a)
Asia/Pacific	×											Zhang et al. (2008b)
**2009**												
Asia/Pacific												Choi et al. (2009)
Africa	×		×		×		×					Klánová et al. (2009)
Asia/Pacific	×	×	×									Li et al. (2009)
Asia/Pacific			×									Liu et al. (2009)

UN Region†	PCBs	FRs	OCPs	CUPs	PAH/PACs	UV Blockers/ Antioxidants	PCDD/Fs	PFAS	Siloxanes	Trace metals	Others	Reference
Global/multi-region	×	×	×									Pozo et al. (2009)
GRULAC			×									Wong et al. (2009)
**2010**												
Global/multi-region								×				Chaemfa et al. (2010)
Asia/Pacific			×									Chakraborty et al. (2010)
Global/multi-region			×									Covaci et al. (2010)
Global/multi-region	×							×				Genualdi et al. (2010)
WEOG					×							Gouin et al. (2010)
WEOG					×							Kennedy et al. (2010)
Global/multi-region	×											Li et al. (2010)
WEOG	×											Persoon et al. (2010)
CEE	×		×									Roots et al. (2010)
Asia/Pacific					×							Wang et al. (2010)
**2011**												
Asia/Pacific			×									Devi et al. (2011)
Global/multi-region									×			Genualdi et al. (2011)
Africa	×											Gioia et al. (2011)
Global/multi-region	×		×		×							Halse et al. (2011)
Global/multi-region	×		×		×							Holoubek et al. (2011)
Asia/Pacific								×				Li et al. (2011)
Asia/Pacific		×										Ma et al. (2011)
Asia/Pacific	×		×									Pozo et al. (2011)
Asia/Pacific			×									Santiago and Cayetano (2011)
CEE	×		×		×							Stafilov et al. (2011)
**2012**												
Africa			×									Adu-Kumi et al. (2012)

UN Region[†]	PCBs	FRs	OCPs	CUPs	PAH/PACs	UV Blockers/ Antioxidants	PCDD/Fs	PFAS	Siloxanes	Trace metals	Others	Reference
CEE	×		×									Aliyeva et al. (2012)
Asia/Pacific					×							Choi et al. (2012)
WEOG	×	×	×		×							Estellano et al. (2012)
WEOG	×		×		×							Halse et al. (2012)
Asia/Pacific			×		×							He and Balasubramanian (2012)
WEOG		×										Hearn et al. (2012)
Africa											PCNs	Hogarh et al. (2012b)
Asia/Pacific	×										PCNs	Hogarh et al. (2012a)
Asia/Pacific	×				×							Kaya et al. (2012)
Global/multi-region		×	×									Koblizkova et al. (2012a)
Global/multi-region				×								Koblizkova et al. (2012b)
Asia/Pacific		×										Li et al. (2012a)
Global/multi-region	×	×										Li et al. (2012b)
GRULAC	×	×										Meire et al. (2012a)
GRULAC			×	×								Meire et al. (2012b)
WEOG	×	×			×							Melymuk et al. (2012)
Africa	×		×	×			×					Moussaoui et al. (2012)
GRULAC	×	×	×		×							Pozo et al. (2012)
CEE	×		×		×							Pribylova et al. (2012)
Asia/Pacific											PCNs	Wang et al. (2012)
**2013**												
Global/multi-region	×		×				×					Bogdal et al. (2013)
Asia/Pacific					×							Cheng et al. (2013)
Global/multi-region	×		×									Fiedler et al. (2013)
Asia/Pacific	×										PCNs	Hogarh et al. (2013)

UN Region†	PCBs	FRs	OCPs	CUPs	PAH/PACs	UV Blockers/ Antioxidants	PCDD/Fs	PFAS	Siloxanes	Trace metals	Others	Reference
Global/multi-region	×		×									Leslie et al. (2013)
Asia/Pacific		×										Lin et al. (2013a)
Asia/Pacific											PCNs	Lin et al. (2013b)
WEOG				×								Mai et al. (2013)
Asia/Pacific	×											Syed et al. (2013a)
Asia/Pacific			×									Syed et al. (2013b)
WEOG					×							Vardar et al. (2013)
Asia/Pacific		×										Wang et al. (2013)
Asia/Pacific					×							Xia et al. (2013)
Asia/Pacific	×		×									Zhang et al. (2013)
Asia/Pacific		×										Zhao et al. (2013)
**2014**												
WEOG									×			Ahrens et al. (2014)
Asia/Pacific		×										Chaemfa et al. (2014)
Asia/Pacific					×							Devi et al. (2014)
WEOG	×				×							Estellano et al. (2014)
Africa			×									Hogarh et al. (2014)
Asia/Pacific		×										Li et al. (2014)
Asia/Pacific	×											Liu et al. (2014)
WEOG			×	×								Messing et al. (2014)
Asia/Pacific			×									Ren et al. (2014)
CEE					×							Sáňka et al. (2014)
GRULAC	×		×									Tombesi et al. (2014)
Asia/Pacific											PCNs	Xu et al. (2014)
Asia/Pacific	×						×					Yoonki et al. (2014)
Asia/Pacific					×							Zhou et al. (2014)

**UN Region†**	**PCBs**	**FRs**	**OCPs**	**CUPs**	**PAH/PACs**	**UV Blockers/ Antioxidants**	**PCDD/Fs**	**PFAS**	**Siloxanes**	**Trace metals**	**Others**	**Reference**
**2015**												
Global/multi-region			×								Natural bromoanisoles	Bidleman et al. (2015)
WEOG				×								Estellano et al. (2015)
Global/multi-region	×	×	×		×							Lammel et al. (2015)
Asia/Pacific		×										Li et al. (2015)
Asia/Pacific					×							Lin et al. (2015)
CEE	×		×									Mamontova et al. (2015)
WEOG		×	×		×							Peverly et al. (2015)
WEOG					×							Pozo et al. (2015)
WEOG			×									Qu et al. (2015)
CEE	×	×					×				PCNs	Roots et al. (2015)
Asia/Pacific					×							Sampath et al. (2015)
GRULAC							×					Schuster et al. (2015)
Asia/Pacific			×									Srimurali et al. (2015)
Asia/Pacific							×					Tian et al. (2015)
Asia/Pacific	×	×										Zhou et al. (2015)
**2016**												
GRULAC	×		×		×							Álvarez et al. (2016)
GRULAC			×									Astoviza et al. (2016)
GRULAC							×					Cappelletti et al. (2016)
GRULAC	×						×					Cortés et al. (2016)
CEE	×	×	×				×					De la Torre et al. (2016)
WEOG		×										Drage et al. (2016)
Asia/Pacific				×								Eng et al. (2016)
Asia/Pacific					×							Evci et al. (2016)

UN Region†	PCBs	FRs	OCPs	CUPs	PAH/PACs	UV Blockers/ Antioxidants	PCDD/Fs	PFAS	Siloxanes	Trace metals	Others	Reference
Global/multi-region	×											Holt et al. (2016)
Asia/Pacific					×							Hong et al. (2016)
Asia/Pacific					×							Kamal et al. (2016)
Global/multi-region		×										Lee et al. (2016)
Asia/Pacific					×							Mao et al. (2016)
CEE	×	×	×				×					Muñoz-Arnanz et al. (2016)
WEOG				×								Pozo et al. (2016a)
WEOG	×	×	×									Pozo et al. (2016b)
GRULAC		×										Rauert et al. (2016)
GRULAC	×		×				×					Tominaga et al. (2016)
Asia/Pacific	×		×									Wang et al. (2016)
Asia/Pacific		×										Zhang et al. (2016a)
Asia/Pacific					×							Zhang et al. (2016b)
**2017**												
WEOG			×	×							Bromoanisoles	Bidleman et al. (2017)
Asia/Pacific	×											Birgül et al. (2017)
WEOG				×	×							Carratalá et al. (2017)
Asia/Pacific	×				×							Cetin et al. (2017)
Asia/Pacific		×										Chakraborty et al. (2017)
Asia/Pacific											NTS	Chung et al. (2017)
Asia/Pacific											PCNs	Die et al. (2017)
Asia/Pacific	×				×							Dumanoglu et al. (2017)
Asia/Pacific					×							Esen et al. (2017)
WEOG	×		×									Estellano et al. (2017)
GRULAC	×						×					Francisco et al. (2017)
Asia/Pacific	×											Gevao et al. (2017)

UN Region†	PCBs	FRs	OCPs	CUPs	PAH/PACs	UV Blockers/ Antioxidants	PCDD/Fs	PFAS	Siloxanes	Trace metals	Others	Reference
Asia/Pacific			×									Huang et al. (2017)
CEE	×		×		×							Kalina et al. (2017)
Asia/Pacific					×							Niu et al. (2017)
Global/multi-region	×		×									Pozo et al. (2017a)
GRULAC	×	×	×									Pozo et al. (2017b)
Asia/Pacific	×	×	×									Pozo et al. (2017c)
Asia/Pacific											Phthalate	Sampath et al. (2017)
Asia/Pacific	×						×					Shahin et al. (2017)
Asia/Pacific			×									Wang et al. (2017)
WEOG			×	×								Wu et al. (2017)
Asia/Pacific	×		×									Yadav et al. (2017)
Asia/Pacific		×										Zhu et al. (2017)
**2018**												
Asia/Pacific	×		×									Ali et al. (2018)
CEE		×										Aliyeva et al. (2018)
GRULAC			×									Arias-Loaiza et al. (2018)
WEOG		×										de la Torre et al. (2018)
GRULAC			×	×								Guida et al. (2018)
Asia/Pacific					×							Hamid et al. (2018)
Asia/Pacific	×	×					×					Hao et al. (2018)
Africa	×											Hogarh et al. (2018)
Africa			×									Isogai et al. (2018)
CEE	×		×		×							Kalina et al. (2018)
Africa		×										Katima et al. (2018)
Asia/Pacific		×										Kurt-Karakus et al. (2018a)
Asia/Pacific	×	×	×									Kurt-Karakus et al. (2018b)

UN Region†	PCBs	FRs	OCPs	CUPs	PAH/PACs	UV Blockers/ Antioxidants	PCDD/Fs	PFAS	Siloxanes	Trace metals	Others	Reference
Asia/Pacific					×							Li et al. (2018)
WEOG	×						×					Muñoz-Arnanz et al. (2018)
CEE	×		×		×							Nikolova et al. (2018)
CEE		×										Okonski et al. (2018)
Asia/Pacific	×		×									Pokhrel et al. (2018a)
Asia/Pacific					×							Pokhrel et al. (2018b)
GRULAC	×		×								PeCA	Rauert et al. (2018a)
GRULAC		×						×	×			Rauert et al. (2018b)
Global/multi-region		×										Rauert et al. (2018c)
Global/multi-region								×	×			Rauert et al. (2018d)
WEOG		×										Roscales et al. (2018)
WEOG	×											Tomsho et al. (2018)
Asia/Pacific								×	×			Wang et al. (2018a)
Asia/Pacific		×										Wang et al. (2018b)
Asia/Pacific					×							Yadav et al. (2018)
Asia/Pacific	×		×									Zhang et al. (2018a)
Asia/Pacific					×							Zhang et al. (2018b)
Asia/Pacific		×										Zhang et al. (2018c)
**2019**												
GRULAC		×										Barrett et al. (2019)
GRULAC			×	×								Climent et al. (2019)
WEOG										×		Gaga et al. (2019)
Asia/Pacific			×									Huang et al. (2019)
Global/multi-region	×	×	×									Kalina et al. (2019)
Asia/Pacific								×				Liu et al. (2019)
GRULAC	×	×	×		×							Pegoraro and Wannaz (2019)

UN Region[†]	PCBs	FRs	OCPs	CUPs	PAH/PACs	UV Blockers/ Antioxidants	PCDD/Fs	PFAS	Siloxanes	Trace metals	Others	Reference
WEOG	×		×		×							Qu et al. (2019)
Asia/Pacific					×							Tong et al. (2019)
Global/multi-region		×	×	×								Wang et al. (2019)
Asia/Pacific					×							Wu et al. (2019)
Asia/Pacific	×											Xu et al. (2019)
Asia/Pacific	×											Zhao et al. (2019)
**2020**												
Asia/Pacific												Cindoruk et al. (2020)
Asia/Pacific					×							Dotel et al. (2020)
GRULAC	×	×	×									Fremlin et al. (2020)
Africa				×								Fuhrimann et al. (2020)
Asia/Pacific		×										Liang et al. (2020)
Asia/Pacific		×										Niu et al. (2020)
Africa	×		×									Pisa et al. (2020)
GRULAC	×	×	×									Ruggeri et al. (2020)
Global/multi-region		×										Saini et al. (2020)
Global/multi-region	×		×		×							Sari et al. (2020)
Asia/Pacific					×							Thang et al. (2020)
Asia/Pacific					×							Wang et al. (2020a)
Asia/Pacific		×										Wang et al. (2020b)
Asia/Pacific					×							Wu et al. (2020)
Asia/Pacific	×											Zhao et al. (2020)
**2021**												
Africa					×							Alani et al. (2021)
Asia/Pacific					×							Çalişkan Eleren and Tasdemir (2021)

UN Region†	PCBs	FRs	OCPs	CUPs	PAH/PACs	UV Blockers/ Antioxidants	PCDD/Fs	PFAS	Siloxanes	Trace metals	Others	Reference
Global/multi-region								×				Camoiras González et al. (2021)
WEOG	×		×									Lunder Halvorsen et al. (2021)
Asia/Pacific					×							Hu et al. (2021)
WEOG	×		×	×								Kruse-Plaß et al. (2021)
Asia/Pacific					×							Liu et al. (2021a)
Global/multi-region		×										Liu et al. (2021b)
Africa		×										Osoro et al. (2021)
WEOG	×	×	×		×							Prats et al. (2021)
Asia/Pacific	×											Prithiviraj et al. (2021)
WEOG	×	×	×		×							Qu et al. (2021)
Asia/Pacific	×	×	×									Riaz et al. (2021)
Asia/Pacific	×											Sari et al. (2021)
Global/multi-region	×		×									Schuster et al. (2021a)
Global/multi-region		×										Schuster et al. (2021b)
Asia/Pacific			×									Sun et al. (2021)
CEE	×	×	×		×							Vakarelska et al. (2021)
WEOG											×	Vasiljevic et al. (2021)
Africa	×	×	×				×					White et al. (2021a)
WEOG	×		×		×							White et al. (2021b)
**2022**												
Global/multi-region	×						×					Abad et al. (2022)
Africa	×	×										Akinrinade et al. (2022)
GRULAC					×							Arias et al. (2022)
Asia/Pacific					×							Aslam et al. (2022)
GRULAC	×	×	×				×	×				Avila et al. (2022)

**UN Region[†]**	**PCBs**	**FRs**	**OCPs**	**CUPs**	**PAH/PACs**	**UV Blockers/ Antioxidants**	**PCDD/Fs**	**PFAS**	**Siloxanes**	**Trace metals**	**Others**	**Reference**
Global/multi-region								×				Fiedler et al. (2022)
Asia/Pacific							×					Gevao et al. (2022)
WEOG	×											(Jahnke et al., 2022)
Global/multi-region						×					Tire wear chemicals	Johannessen et al. (2022)
weog					×							Klingberg et al. (2022)
GRULAC			×									Llanos et al. (2022)
CEE	×		×									Mamontova and Mamontov (2022)
GRULAC	×	×	×				×					Martínez Valenzuela et al. (2022)
Asia/Pacific					×							Nargis et al. (2022)
Africa		×										Nipen et al. (2022)
WEOG	×	×	×	×	×							Platt et al. (2022)
GRULAC	×	×	×		×							Pozo et al. (2022)
WEOG	×	×	×		×						PAEs	Qu et al. (2022)
Asia/Pacific												Sanli and Tasdemir (2022)
Asia/Pacific	×		×		×							Sari and Esen (2022)
Asia/Pacific		×										Sun et al. (2022)
Africa			×	×								Veludo et al. (2022)
Asia/Pacific								×				Wang et al. (2022a)
Asia/Pacific								×				Wang et al. (2022b)
**2023**												
Asia/Pacific			×									Alshemmari et al. (2023)
Asia/Pacific											PAEs	Chandra and Chakraborty (2023)

UN Region†	PCBs	FRs	OCPs	CUPs	PAH/PACs	UV Blockers/ Antioxidants	PCDD/Fs	PFAS	Siloxanes	Trace metals	Others	Reference
Global/multi-region	×	×	×									de Boer et al. (2023)
WEOG					×							Delaunay et al. (2023)
Asia/Pacific	×											Eker Sanli and Tasdemir (2023)
Asia/Pacific	×		×		×							Esen et al. (2023)
Global/multi-region	×		×				×					Fiedler et al. (2023)
WEOG	×		×									Halvorsen et al. (2023)
Global/multi-region		×										He et al. (2023)
Global/multi-region	×		×									Luarte et al. (2023)
Global/multi-region										×		Mastin et al. (2023)
Africa	×	×	×				×	×				Ouertani et al. (2023)
Asia/Pacific					×							Qi et al. (2023)
Global/multi-region								×	×			Saini et al. (2023)
Asia/Pacific					×							Sanli et al. (2023)
Asia/Pacific	×											Sari et al. (2023)
Asia/Pacific	×						×					Sau (2023)
Global/multi-region	×		×									Schuster et al. (2023)
Asia/Pacific								×				Shen et al. (2023)
Global/multi-region		×										Skogeng et al. (2023)
Asia/Pacific	×		×									Sohail et al. (2023)
Asia/Pacific	×	×	×					×				Surenjav and Fiedler (2023)
Africa					×							Ukpebor et al. (2023)
Asia/Pacific	×	×	×		×							Vlahos et al. (2023)
Asia/Pacific					×							Wang et al. (2023)
Global/multi-region	×	×	×				×	×				White et al. (2023)
Global/multi-region												Wu et al. (2023)

UN Region†	PCBs	FRs	OCPs	CUPs	PAH/PACs	UV Blockers/ Antioxidants	PCDD/Fs	PFAS	Siloxanes	Trace metals	Others	Reference
WEOG	×			×								Zaller et al. (2023)
Asia/Pacific				×								Zhao et al. (2023)
**2024**												
Africa		×										Arko et al. (2024)
Asia/Pacific	×											Eker Sanli et al. (2024)
Global/multi-region		×	×				×	×				Fiedler et al. (2024)
WEOG		×			×							Rosa et al. (2024)
Asia/Pacific		×										Tahir et al. (2024)
Global/multi-region					×							Valdivia et al. (2024)
Asia/Pacific					×							Van Vu et al. (2024)
Asia/Pacific					×							Wang et al. (2024a)
Asia/Pacific		×										Wang et al. (2024b)
WEOG								×				Xia et al. (2024)
Asia/Pacific		×										Zhang et al. (2024)

References

Abad, E., Abalos, M., Fiedler, H., 2022. Air monitoring with passive samplers for dioxin-like persistent organic pollutants in developing countries (2017–2019). Chemosphere 287, 131931.

Adu-Kumi, S., Kareš, R., Literák, J., Borůvková, J., Yeboah, P.O., Carboo, D., Akoto, O., Darko, G., Osae, S., Klánová, J., 2012. Levels and seasonal variations of organochlorine pesticides in urban and rural background air of southern Ghana. Environmental Science and Pollution Research 19, 1963-1970.

Ahrens, L., Harner, T., Shoeib, M., 2014. Temporal variations of cyclic and linear volatile methylsiloxanes in the atmosphere using passive samplers and high-volume air samplers. Environmental Science & Technology 48, 9374-9381.

Akinrinade, O.E., Stubbings, W.A., Abdallah, M.A.-E., Ayejuyo, O., Alani, R., Harrad, S., 2022. Atmospheric concentrations of polychlorinated biphenyls, brominated flame retardants, and novel flame retardants in lagos, Nigeria indicate substantial local sources. Environmental Research 204, 112091.

Alani, R., Zhao, S., Liu, X., Akinrinade, O., Agunbiade, F., Ayejuyo, O., Zhang, G., 2021. Concentrations, profiles and exposure risks of polycyclic aromatic hydrocarbons (PAHs) in passive air samples from lagos, Nigeria. Atmospheric Pollution Research 12, 101162.

Alegria, H.A., Wong, F., Jantunen, L.M., Bidleman, T.F., Figueroa, M.S., Bouchot, G.G., Moreno, V.C., Waliszewski, S.M., Infanzon, R., 2008. Organochlorine pesticides and PCBs in air of southern mexico (2002–2004). Atmospheric Environment 42, 8810-8818.

Ali, U., Sweetman, A.J., Jones, K.C., Malik, R.N., 2018. Higher atmospheric levels and contribution of black carbon in soil-air partitioning of organochlorines in lesser himalaya. Chemosphere 191, 787-798.

Aliyeva, G., Kurkova, R., Hovorkova, I., Klánová, J., Halsall, C., 2012. Organochlorine pesticides and polychlorinated biphenyls in air and soil across azerbaijan. Environmental Science and Pollution Research 19, 1953-1962.

Aliyeva, G., Sinnott-Clark, C.A., Audy, O., Škrdlíková, L., Kukučka, P., Klánová, J., Halsall, C., 2018. A contemporary assessment of polybrominated diphenyl ethers (PBDE) in the ambient air and soil of azerbaijan. Environmental science and pollution research international 25, 31863-31873.

Alshemmari, H., Al-Kasbi, M.M., Kavil, Y.N., Orif, M.I., Al-Hulwani, E.K., Al-Darii, R.J., Al-Shukaili, S.M., Al-Balushi, F.A.A., Chakraborty, P., 2023. New and legacy pesticidal persistent organic pollutants in the agricultural region of the sultanate of oman. Journal of Hazardous Materials 459, 132205.

Álvarez, Á.L., Pozo, K., Paéz, M.I., Estellano, V.H., Llanos, Y., Focardi, S., 2016. Semivolatile organic compounds (SVOCs) in the atmosphere of Santiago de cali, valle del cauca, colombia along north-south transect using polyurethane foam disk as passive air samplers. Atmospheric Pollution Research 7, 945-953.

Arias-Loaiza, G.E., Beristain-Montiel, E., Gómez-Arroyo, S., Amador-Muñoz, O., 2018. A thermo-cavitation method to determine organochlorine pesticides in gas and particle phases collected in polyurethane foam used in passive air samplers. Water, Air, & Soil Pollution 229, 282.

Arias, A.H., Pozo, K.A., Álvarez, M.B., Pribylová, P., Tombesi, N.B., 2022. Atmospheric PAHs in rural, urban, industrial and beach locations in buenos aires province, argentina:

Sources and health risk assessment. Environmental Geochemistry and Health 44, 2419-2433.
Arko, W.E., Zhao, S., Ma, J., Tian, L., Asante, K.A., Amoah, D.K., Qi, S., Zhang, G., 2024. Impact of anthropogenic activities on atmospheric chlorinated paraffins in Ghana using polyurethane foam disk - passive air sampler. Science of The Total Environment 954, 176252.
Aslam, R., Sharif, F., Baqar, M., Shahzad, L., 2022. Source identification and risk assessment of polycyclic aromatic hydrocarbons (PAHs) in air and dust samples of Lahore city. Scientific Reports 12, 2459.
Astoviza, M.J., Cappelletti, N., Bilos, C., Migoya, M.C., Colombo, J.C., 2016. Massive airborne endosulfan inputs related to intensive agriculture in Argentina's Pampa. Chemosphere 144, 1459-1466.
Avila, B.S., Mendoza, D.P., Ramírez, A., Peñuela, G.A., 2022. Occurrence and distribution of persistent organic pollutants (POPs) in the atmosphere of the andean city of medellin, colombia. Chemosphere 307, 135648.
Barber, J.L., Sweetman, A.J., Thomas, G.O., Braekevelt, E., Stern, G.A., Jones, K.C., 2005. Spatial and temporal variability in air concentrations of short-chain (C10− C13) and medium-chain (C14− C17) chlorinated n-alkanes measured in the UK atmosphere. Environmental Science & Technology 39, 4407-4415.
Barrett, K.S.C., Jaward, F.M., Stuart, A.L., 2019. Forest filter effect for polybrominated diphenyl ethers in a tropical watershed. Journal of environmental management 248, 109279.
Bidleman, T., Agosta, K., Andersson, A., Brorström-Lundén, E., Haglund, P., Hansson, K., Laudon, H., Newton, S., Nygren, O., Ripszam, M., Tysklind, M., Wiberg, K., 2015. Atmospheric pathways of chlorinated pesticides and natural bromoanisoles in the northern Baltic sea and its catchment. Ambio 44 Suppl 3, 472-483.
Bidleman, T.F., Laudon, H., Nygren, O., Svanberg, S., Tysklind, M., 2017. Chlorinated pesticides and natural brominated anisoles in air at three northern Baltic stations. Environmental Pollution 225, 381-389.
Birgül, A., Kurt-Karakus, P.B., Alegria, H., Gungormus, E., Celik, H., Cicek, T., Güven, E.C., 2017. Polyurethane foam (PUF) disk passive samplers derived polychlorinated biphenyls (PCBs) concentrations in the ambient air of Bursa-Turkey: Spatial and temporal variations and health risk assessment. Chemosphere 168, 1345-1355.
Bogdal, C., Scheringer, M., Abad, E., Abalos, M., Van Bavel, B., Hagberg, J., Fiedler, H., 2013. Worldwide distribution of persistent organic pollutants in air, including results of air monitoring by passive air sampling in five continents. TrAC Trends in Analytical Chemistry 46, 150-161.
Çalişkan Eleren, S., Tasdemir, Y., 2021. Levels, distributions, and seasonal variations of polycyclic aromatic hydrocarbons (PAHs) in ambient air and pine components. Environmental Monitoring and Assessment 193, 253.
Camoiras González, P., Sadia, M., Baabish, A., Sobhanei, S., Fiedler, H., 2021. Air monitoring with passive samplers for perfluoroalkane substances in developing countries (2017-2019). Chemosphere 282, 131069.
Cappelletti, N., Astoviza, M., Migoya, M.C., Colombo, J.C., 2016. Airborne PCDD/f profiles in rural and urban areas of buenos aires province, argentina. Science of The Total Environment 573, 1406-1412.

Carratalá, A., Moreno-González, R., León, V.M., 2017. Occurrence and seasonal distribution of polycyclic aromatic hydrocarbons and legacy and current-use pesticides in air from a Mediterranean coastal lagoon (mar menor, se Spain). Chemosphere 167, 382-395.
Cetin, B., Ozturk, F., Keles, M., Yurdakul, S., 2017. PAHs and PCBs in an eastern Mediterranean megacity, Istanbul: Their spatial and temporal distributions, air-soil exchange and toxicological effects. Environmental Pollution 220, 1322-1332.
Chaemfa, C., Barber, J.L., Huber, S., Breivik, K., Jones, K.C., 2010. Screening for PFOS and PFOA in European air using passive samplers. Journal of Environmental Monitoring 12, 1100-1109.
Chaemfa, C., Xu, Y., Li, J., Chakraborty, P., Hussain Syed, J., Naseem Malik, R., Wang, Y., Tian, C., Zhang, G., Jones, K.C., 2014. Screening of atmospheric short- and medium-chain chlorinated paraffins in India and Pakistan using polyurethane foam based passive air sampler. Environmental Science & Technology 48, 4799-4808.
Chakraborty, P., Zhang, G., Cheng, H., Balasubramanian, P., Li, J., Jones, K.C., 2017. Passive air sampling of polybrominated diphenyl ethers in new delhi, kolkata, mumbai and chennai: Levels, homologous profiling and source apportionment. Environmental Pollution 231, 1181-1187.
Chakraborty, P., Zhang, G., Li, J., Xu, Y., Liu, X., Tanabe, S., Jones, K.C., 2010. Selected organochlorine pesticides in the atmosphere of major Indian cities: Levels, regional versus local variations, and sources. Environmental Science & Technology 44, 8038-8043.
Chandra, S., Chakraborty, P., 2023. Air-water exchange and risk assessment of phthalic acid esters during the early phase of covid-19 pandemic in tropical riverine catchments of India. Chemosphere 341, 140013.
Cheng, H., Deng, Z., Chakraborty, P., Liu, D., Zhang, R., Xu, Y., Luo, C., Zhang, G., Li, J., 2013. A comparison study of atmospheric polycyclic aromatic hydrocarbons in three Indian cities using PUF disk passive air samplers. Atmospheric Environment 73, 16-21.
Choi, S.-D., Baek, S.-Y., Chang, Y.-S., 2009. Passive air sampling of persistent organic pollutants in Korea. Toxicology and Environmental Health Sciences 1, 75-82.
Choi, S.-D., Kwon, H.-O., Lee, Y.-S., Park, E.-J., Oh, J.-Y., 2012. Improving the spatial resolution of atmospheric polycyclic aromatic hydrocarbons using passive air samplers in a multi-industrial city. Journal of Hazardous Materials 241, 252-258.
Chung, I.-Y., Park, Y.-M., Lee, H.-J., Kim, H., Kim, D.-H., Kim, I.-G., Kim, S.-M., Do, Y.-S., Seok, K.-S., Kwon, J.-H., 2017. Nontarget screening using passive air and water sampling with a level II fugacity model to identify unregulated environmental contaminants. Journal of Environmental Sciences 62, 84-91.
Cindoruk, S.S., Sakin, A.E., Tasdemir, Y., 2020. Levels of persistent organic pollutants in pine tree components and ambient air. Environmental Pollution 256, 113418.
Climent, M.J., Coscollà, C., López, A., Barra, R., Urrutia, R., 2019. Legacy and current-use pesticides (CUPs) in the atmosphere of a rural area in central Chile, using passive air samplers. Science of The Total Environment 662, 646-654.
Cortés, J., Cobo, M., González, C.M., Gómez, C.D., Abalos, M., Aristizábal, B.H., 2016. Environmental variation of PCDD/fs and dl-PCBs in two tropical andean colombian cities using passive samplers. Science of The Total Environment 568, 614-623.

Covaci, A., Gheorghe, A., Meijer, S., Jaward, F., Jantunen, L., Neels, H., Jones, K.C., 2010. Investigation of source apportioning for α-HCH using enantioselective analysis. Environment International 36, 316-322.
de Boer, J., van Dijk, R., Abalos, M., Abad, E., 2023. Persistent organic pollutants in air from Asia, Africa, Latin America, and the Pacific. Chemosphere 324, 138271.
de la Torre, A., Barbas, B., Sanz, P., Navarro, I., Artíñano, B., Martínez, M.A., 2018. Traditional and novel halogenated flame retardants in urban ambient air: Gas-particle partitioning, size distribution and health implications. Science of The Total Environment 630, 154-163.
De la Torre, A., Sanz, P., Navarro, I., Martínez, M.Á., 2016. Time trends of persistent organic pollutants in Spanish air. Environmental Pollution 217, 26-32.
Delaunay, B., Sauret, N., Ledauphin, J., 2023. Novel eco-friendly methodology to determine polycyclic aromatic hydrocarbons in polyurethane foam for air monitoring: Application to spatial and temporal distribution survey. Chemosphere 311, 137059.
Devi, N.L., Qi, S., Chakraborty, P., Zhang, G., Yadav, I.C., 2011. Passive air sampling of organochlorine pesticides in a northeastern state of India, manipur. Journal of Environmental Sciences 23, 808-815.
Devi, N.L., Shihua, Q., Yadav, I.C., 2014. Atmospheric polycyclic aromatic hydrocarbons (PAH) in manipur of the northeast India: Monitoring on urban, rural, and mountain sites. Polycyclic Aromatic Compounds 34, 12-34.
Die, Q., Nie, Z., Yue, B., Zhu, X., Gao, X., Wang, J., Yang, Y., Fang, Y., Huang, Q., 2017. Assessment of the temporal and spatial distribution of atmospheric PCNs and their air–soil exchange using passive air samplers in Shanghai, East China. Environmental Science and Pollution Research 24, 14365-14375.
Dotel, J., Gong, P., Wang, X., Pokhrel, B., Wang, C., Nawab, J., 2020. Determination of dry deposition velocity of polycyclic aromatic hydrocarbons under the sub-tropical climate and its implication for regional cycling. Environmental Pollution 261, 114143.
Drage, D.S., Newton, S., de Wit, C.A., Harrad, S., 2016. Concentrations of legacy and emerging flame retardants in air and soil on a transect in the UK west midlands. Chemosphere 148, 195-203.
Dumanoglu, Y., Gaga, E.O., Gungormus, E., Sofuoglu, S.C., Odabasi, M., 2017. Spatial and seasonal variations, sources, air-soil exchange, and carcinogenic risk assessment for PAHs and PCBs in air and soil of Kutahya, Turkey, the province of thermal power plants. Science of The Total Environment 580, 920-935.
Eker Sanli, G., Erkul, S.N., Tasdemir, Y., 2024. Spatio-temporal variations, fugacity fractions and air-soil exchanges of PCBs in industrial, urban and semi-rural sites. Polycyclic Aromatic Compounds 44, 1019-1036.
Eker Sanli, G., Tasdemir, Y., 2023. Temporal variations of PCBs and their estimated air-soil exchange fluxes measured in seven sites in Bursa-Turkey. Soil and Sediment Contamination: An International Journal 32, 843-861.
Eng, A., Su, K., Harner, T., Pozo, K., Sinha, R.K., Sengupta, B., Loewen, M., 2016. Assessing dicofol concentrations in air: Retrospective analysis of global atmospheric passive sampling network samples from agricultural sites in India. Environmental Science & Technology Letters 3, 150-155.

Esen, F., Cordova Del Aguila, D.A., Sari, M.F., 2023. Air and soil concentrations of persistent organic pollutants in Bursa (Türkiye) and Yurimaguas (Peru): Air-soil exchange and gas-phase flux. Air Quality, Atmosphere & Health 16, 2239-2255.
Esen, F., Evci, Y.M., Tasdemir, Y., 2017. Evaluation and application of a passive air sampler for polycylic aromatic hydrocarbons (PAHs). Journal of Environmental Science and Health, Part A 52, 1022-1029.
Estellano, V.H., Pozo, K., Efstathiou, C., Pozo, K., Corsolini, S., Focardi, S., 2015. Assessing levels and seasonal variations of current-use pesticides (CUPs) in the tuscan atmosphere, italy, using polyurethane foam disks (PUF) passive air samplers. Environmental Pollution 205, 52-59.
Estellano, V.H., Pozo, K., Harner, T., Corsolini, S., Focardi, S., 2012. Using PUF disk passive samplers to simultaneously measure air concentrations of persistent organic pollutants (POPs) across the Tuscany region, italy. Atmospheric Pollution Research 3, 88-94.
Estellano, V.H., Pozo, K., Harner, T., Franken, M., Zaballa, M., 2008. Altitudinal and seasonal variations of persistent organic pollutants in the bolivian andes mountains. Environmental Science & Technology 42, 2528-2534.
Estellano, V.H., Pozo, K., Přibylová, P., Klánová, J., Audy, O., Focardi, S., 2017. Assessment of seasonal variations in persistent organic pollutants across the region of Tuscany using passive air samplers. Environmental Pollution 222, 609-616.
Estellano, V.H., Pozo, K., Silibello, C., Mulder, M.D., Efstathiou, C., Tomasino, M.P., Funaro, F., Donadio, I., Focardi, S., 2014. Characterization of urban pollution in two cities of the puglia region in southern italy using field measurements and air quality (aq) model approach. Atmospheric Pollution Research 5, 34-41.
Evci, Y.M., Esen, F., Taşdemir, Y., 2016. Monitoring of long-term outdoor concentrations of PAHs with passive air samplers and comparison with meteorological data. Archives of Environmental Contamination and Toxicology 71, 246-256.
Fiedler, H., Abad, E., de Boer, J., 2023. Preliminary trends over ten years of persistent organic pollutants in air-comparison of two sets of data in the same countries. Chemosphere 324, 138299.
Fiedler, H., Abad, E., van Bavel, B., de Boer, J., Bogdal, C., Malisch, R., 2013. The need for capacity building and first results for the Stockholm Convention global monitoring plan. TrAC Trends in Analytical Chemistry 46, 72-84.
Fiedler, H., Baabish, A., Sadia, M., 2022. Multivariate analysis of abiotic and biota samples for three perfluoroalkane acids. Frontiers in Analytical Science Volume 2 - 2022.
Fiedler, H., de Boer, J., Abad, E., 2024. Persistent organic pollutants in air across the globe using a comparative passive air sampling method. TrAC Trends in Analytical Chemistry 171, 117494.
Francisco, A.P., Nardocci, A.C., Tominaga, M.Y., da Silva, C.R., de Assunção, J.V., 2017. Spatial and seasonal trends of polychlorinated dioxins, furans and dioxin-like polychlorinated biphenyls in air using passive and active samplers and inhalation risk assessment. Atmospheric Pollution Research 8, 979-987.
Fremlin, K.M., Elliott, J.E., Green, D.J., Drouillard, K.G., Harner, T., Eng, A., Gobas, F., 2020. Trophic magnification of legacy persistent organic pollutants in an urban terrestrial food web. Science of The Total Environment 714, 136746.

Fuhrimann, S., Klánová, J., Přibylová, P., Kohoutek, J., Dalvie, M.A., Röösli, M., Degrendele, C., 2020. Qualitative assessment of 27 current-use pesticides in air at 20 sampling sites across Africa. Chemosphere 258, 127333.

Gaga, E.O., Harner, T., Dabek-Zlotorzynska, E., Celo, V., Evans, G., Jeong, C.-H., Halappanavar, S., Jariyasopit, N., Su, Y., 2019. Polyurethane foam (PUF) disk samplers for measuring trace metals in ambient air. Environmental Science & Technology Letters 6, 545-550.

Genualdi, S., Harner, T., Cheng, Y., MacLeod, M., Hansen, K.M., van Egmond, R., Shoeib, M., Lee, S.C., 2011. Global distribution of linear and cyclic volatile methyl siloxanes in air. Environmental Science & Technology 45, 3349-3354.

Genualdi, S., Lee, S.C., Shoeib, M., Gawor, A., Ahrens, L., Harner, T., 2010. Global pilot study of legacy and emerging persistent organic pollutants using sorbent-impregnated polyurethane foam disk passive air samplers. Environmental Science & Technology 44, 5534-5539.

Gevao, B., Al-Omair, A., Sweetman, A., Al-Ali, L., Al-Bahloul, M., Helaleh, M., Zafar, J., 2006. Passive sampler-derived air concentrations for polybrominated diphenyl ethers and polycyclic aromatic hydrocarbons in Kuwait. Environmental Toxicology and Chemistry 25, 1496-1502.

Gevao, B., Martinez-Guijarro, K., Kurt-Karakus, P.B., Sukhn, C., Weber, R., Krishnan, D., Rajagopalan, S., Birgul, A., Alshemmari, H., Hajeyah, M., Bahloul, M., Orif, M.I., 2022. Spatial variability in the ambient concentrations of polychlorinated dibenzo-p-dioxins and polychlorinated dibenzofurans across the middle east. Atmospheric Pollution Research 13, 101613.

Gevao, B., Porcelli, M., Rajagopalan, S., Krishnan, D., Martinez-Guijarro, K., Alshemmari, H., Bahloul, M., Zafar, J., 2017. Seasonal variations in the atmospheric concentrations of polychlorinated biphenyls in Kuwait. Chemosphere 189, 652-660.

Gioia, R., Eckhardt, S., Breivik, K., Jaward, F.M., Prieto, A., Nizzetto, L., Jones, K.C., 2011. Evidence for major emissions of PCBs in the West African region. Environmental Science & Technology 45, 1349-1355.

Gioia, R., Sweetman, A.J., Jones, K.C., 2007. Coupling passive air sampling with emission estimates and chemical fate modeling for persistent organic pollutants (POPs): A feasibility study for northern Europe. Environmental Science & Technology 41, 2165-2171.

Gouin, T., Harner, T., Blanchard, P., Mackay, D., 2005. Passive and active air samplers as complementary methods for investigating persistent organic pollutants in the Great Lakes basin. Environmental Science & Technology 39, 9115-9122.

Gouin, T., Shoeib, M., Harner, T., 2008. Atmospheric concentrations of current-use pesticides across south-central ontario using monthly-resolved passive air samplers. Atmospheric Environment 42, 8096-8104.

Gouin, T., Wilkinson, D., Hummel, S., Meyer, B., Culley, A., 2010. Polycyclic aromatic hydrocarbons in air and snow from fairbanks, Alaska. Atmospheric Pollution Research 1, 9-15.

Guida, Y.d.S., Meire, R.O., Torres, J.P.M., Malm, O., 2018. Air contamination by legacy and current-use pesticides in brazilian mountains: An overview of national regulations by monitoring pollutant presence in pristine areas. Environmental Pollution 242, 19-30.

Halse, A.K., Schlabach, M., Eckhardt, S., Sweetman, A., Jones, K.C., Breivik, K., 2011. Spatial variability of POPs in European background air. Atmospheric Chemistry and Physics 11, 1549-1564.

Halse, A.K., Schlabach, M., Sweetman, A., Jones, K.C., Breivik, K., 2012. Using passive air samplers to assess local sources versus long range atmospheric transport of POPs. Journal of Environmental Monitoring 14, 2580-2590.

Halvorsen, H.L., Bohlin-Nizzetto, P., Eckhardt, S., Gusev, A., Moeckel, C., Shatalov, V., Skogeng, L.P., Breivik, K., 2023. Spatial variability and temporal changes of POPs in European background air. Atmospheric Environment 299, 119658.

Hamid, N., Syed, J.H., Junaid, M., Mahmood, A., Li, J., Zhang, G., Malik, R.N., 2018. Elucidating the urban levels, sources and health risks of polycyclic aromatic hydrocarbons (PAHs) in Pakistan: Implications for changing energy demand. Science of The Total Environment 619-620, 165-175.

Hao, Y., Li, Y., Wang, T., Hu, Y., Sun, H., Matsiko, J., Zheng, S., Wang, P., Zhang, Q., 2018. Distribution, seasonal variation and inhalation risks of polychlorinated dibenzo-p-dioxins and dibenzofurans, polychlorinated biphenyls and polybrominated diphenyl ethers in the atmosphere of Beijing, China. Environmental Geochemistry and Health 40, 1907-1918.

Harner, T., Pozo, K., Gouin, T., Macdonald, A.-M., Hung, H., Cainey, J., Peters, A., 2006a. Global pilot study for persistent organic pollutants (POPs) using PUF disk passive air samplers. Environmental Pollution 144, 445-452.

Harner, T., Shoeib, M., Diamond, M., Ikonomou, M., Stern, G., 2006b. Passive sampler derived air concentrations of PBDEs along an urban–rural transect: Spatial and temporal trends. Chemosphere 64, 262-267.

Harner, T., Shoeib, M., Diamond, M., Stern, G., Rosenberg, B., 2004. Using passive air samplers to assess urban− rural trends for persistent organic pollutants. 1. Polychlorinated biphenyls and organochlorine pesticides. Environmental Science & Technology 38, 4474-4483.

Harner, T., Shoeib, M., Gouin, T., Blanchard, P., 2006c. Polychlorinated naphthalenes in Great Lakes air: Assessing spatial trends and combustion inputs using PUF disk passive air samplers. Environmental Science & Technology 40, 5333-5339.

Harrad, S., Hunter, S., 2006. Concentrations of polybrominated diphenyl ethers in air and soil on a rural− urban transect across a major UK conurbation. Environmental Science & Technology 40, 4548-4553.

He, C., Thai, P.K., Bertrand, L., Jayarathne, A., van Mourik, L., Phuc, D.H., Banks, A., Mueller, J.F., Wang, X.F., 2023. Calibration and application of PUF disk passive air samplers to assess chlorinated paraffins in ambient air in Australia, China, and Vietnam. Environmental Science & Technology 57, 21061-21070.

He, J., Balasubramanian, R., 2012. Passive sampling of gaseous persistent organic pollutants in the atmosphere. Energy Procedia 16, 494-500.

Hearn, L.K., Kennedy, K., Hawker, D.W., Toms, L.-M.L., Alberts, V., Mueller, J.F., 2012. Spatial mapping of city-wide PBDE levels using an exponential decay model. Journal of Environmental Monitoring 14, 643-650.

Hogarh, J.N., Seike, N., Kobara, Y., Carboo, D., Fobil, J.N., Masunaga, S., 2018. Source characterization and risk of exposure to atmospheric polychlorinated biphenyls (PCBs) in Ghana. Environmental Science and Pollution Research 25, 16316-16324.

Hogarh, J.N., Seike, N., Kobara, Y., Habib, A., Nam, J.-J., Lee, J.-S., Li, Q., Liu, X., Li, J., Zhang, G., Masunaga, S., 2012a. Passive air monitoring of PCBs and PCNs across East Asia: A comprehensive congener evaluation for source characterization. Chemosphere 86, 718-726.

Hogarh, J.N., Seike, N., Kobara, Y., Masunaga, S., 2012b. Atmospheric polychlorinated naphthalenes in Ghana. Environmental Science & Technology 46, 2600-2606.

Hogarh, J.N., Seike, N., Kobara, Y., Masunaga, S., 2013. Seasonal variation of atmospheric polychlorinated biphenyls and polychlorinated naphthalenes in Japan. Atmospheric Environment 80, 275-280.

Hogarh, J.N., Seike, N., Kobara, Y., Ofosu-Budu, G.K., Carboo, D., Masunaga, S., 2014. Atmospheric burden of organochlorine pesticides in Ghana. Chemosphere 102, 1-5.

Holoubek, I., Klanova, J., Cupr, P., Kukucka, P., Borůvková, J., Kohoutek, J., Prokeš, R., Kareš, R., 2011. POPs in ambient air from MONET network - global and regional trends. WIT Transactions on Ecology and the Environment 147, 173-184.

Holt, E., Kočan, A., Klánová, J., Assefa, A., Wiberg, K., 2016. Spatiotemporal patterns and potential sources of polychlorinated biphenyl (PCB) contamination in scots pine (pinus sylvestris) needles from Europe. Environmental Science and Pollution Research 23, 19602-19612.

Hong, W.-J., Jia, H., Ma, W.-L., Sinha, R.K., Moon, H.-B., Nakata, H., Minh, N.H., Chi, K.H., Li, W.-L., Kannan, K., Sverko, E., Li, Y.-F., 2016. Distribution, fate, inhalation exposure and lung cancer risk of atmospheric polycyclic aromatic hydrocarbons in some Asian countries. Environmental Science & Technology 50, 7163-7174.

Hu, T., Mao, Y., Ke, Y., Liu, W., Cheng, C., Shi, M., Zhang, Z., Zhang, J., Qi, S., Xing, X., 2021. Spatial and seasonal variations of PAHs in soil, air, and atmospheric bulk deposition along the plain to mountain transect in Hubei province, central China: Air-soil exchange and long-range atmospheric transport. Environmental Pollution 291, 118139.

Huang, H., Ding, Y., Chen, W., Zhang, Y., Chen, W., Chen, Y., Mao, Y., Qi, S., 2019. Two-way long-range atmospheric transport of organochlorine pesticides (OCPs) between the yellow river source and the sichuan basin, western China. Science of The Total Environment 651, 3230-3240.

Huang, Y., Zhang, R., Li, K., Cheng, Z., Zhong, G., Zhang, G., Li, J., 2017. Experimental study on the role of sedimentation and degradation processes on atmospheric deposition of persistent organic pollutants in a subtropical water column. Environmental Science & Technology 51, 4424-4433.

Isogai, N., Hogarh, J.N., Seike, N., Kobara, Y., Oyediran, F., Wirmvem, M.J., Ayonghe, S.N., Fobil, J., Masunaga, S., 2018. Atmospheric monitoring of organochlorine pesticides across some West African countries. Environmental science and pollution research international 25, 31828-31835.

Jahnke, J.C., Martinez, A., Hornbuckle, K.C., 2022. Distinguishing aroclor and non-aroclor sources to chicago air. Science of The Total Environment 823, 153263.

Jaward, F.M., Di Guardo, A., Nizzetto, L., Cassani, C., Raffaele, F., Ferretti, R., Jones, K.C., 2005a. PCBs and selected organochlorine compounds in italian mountain air: The influence of altitude and forest ecosystem type. Environmental Science & Technology 39, 3455-3463.

Jaward, F.M., Farrar, N.J., Harner, T., Sweetman, A.J., Jones, K.C., 2004a. Passive air sampling of PCBs, PBDEs, and organochlorine pesticides across Europe. Environmental Science & Technology 38, 34-41.

Jaward, F.M., Farrar, N.J., Harner, T., Sweetman, A.J., Jones, K.C., 2004b. Passive air sampling of polycyclic aromatic hydrocarbons and polychlorinated naphthalenes across Europe. Environmental Toxicology and Chemistry: An International Journal 23, 1355-1364.

Jaward, F.M., Zhang, G., Nam, J.J., Sweetman, A.J., Obbard, J.P., Kobara, Y., Jones, K.C., 2005b. Passive air sampling of polychlorinated biphenyls, organochlorine compounds, and polybrominated diphenyl ethers across Asia. Environmental Science & Technology 39, 8638-8645.

Johannessen, C., Saini, A., Zhang, X., Harner, T., 2022. Air monitoring of tire-derived chemicals in global megacities using passive samplers. Environmental Pollution 314, 120206.

Kalina, J., Scheringer, M., Borůvková, J., Kukučka, P., Přibylová, P., Bohlin-Nizzetto, P., Klánová, J., 2017. Passive air samplers as a tool for assessing long-term trends in atmospheric concentrations of semivolatile organic compounds. Environmental Science & Technology 51, 7047-7054.

Kalina, J., Scheringer, M., Borůvková, J., Kukučka, P., Přibylová, P., Sáňka, O., Melymuk, L., Váňa, M., Klánová, J., 2018. Characterizing spatial diversity of passive sampling sites for measuring levels and trends of semivolatile organic chemicals. Environmental Science & Technology 52, 10599-10608.

Kalina, J., White, K.B., Scheringer, M., Přibylová, P., Kukučka, P., Audy, O., Klánová, J., 2019. Comparability of long-term temporal trends of POPs from co-located active and passive air monitoring networks in Europe. Environmental Science: Processes & Impacts 21, 1132-1142.

Kamal, A., Syed, J.H., Li, J., Zhang, G., Mahmood, A., Malik, R.N., 2016. Profile of atmospheric PAHs in rawalpindi, Lahore and gujranwala districts of Punjab province (Pakistan). Aerosol and Air Quality Research 16, 1010-1021.

Katima, Z.J., Olukunle, O.I., Kalantzi, O.-l., Daso, A.P., Okonkwo, J.O., 2018. The occurrence of brominated flame retardants in the atmosphere of gauteng province, South Africa using polyurethane foam passive air samplers and assessment of human exposure. Environmental Pollution 242, 1894-1903.

Kaya, E., Dumanoglu, Y., Kara, M., Altiok, H., Bayram, A., Elbir, T., Odabasi, M., 2012. Spatial and temporal variation and air–soil exchange of atmospheric PAHs and PCBs in an industrial region. Atmospheric Pollution Research 3, 435-449.

Kennedy, K., Macova, M., Bartkow, M.E., Hawker, D.W., Zhao, B., Denison, M.S., Mueller, J.F., 2010. Effect based monitoring of seasonal ambient air exposures in Australia sampled by PUF passive air samplers. Atmospheric Pollution Research 1, 50-58.

Klánová, J., Čupr, P., Holoubek, I., Borůvková, J., Přibylová, P., Kareš, R., Tomšej, T., Ocelka, T., 2009. Monitoring of persistent organic pollutants in Africa. Part 1: Passive air sampling across the continent in 2008. Journal of Environmental Monitoring 11, 1952-1963.

Klánová, J., Kohoutek, J., Čupr, P., Holoubek, I., 2007. Are the residents of former Yugoslavia still exposed to elevated PCB levels due to the Balkan wars? Part 2: Passive air sampling network. Environment International 33, 727-735.

Klánová, J., Kohoutek, J., Hamplová, L., Urbanová, P., Holoubek, I., 2006. Passive air sampler as a tool for long-term air pollution monitoring: Part 1. Performance assessment for seasonal and spatial variations. Environmental Pollution 144, 393-405.

Klingberg, J., Strandberg, B., Sjöman, H., Taube, M., Wallin, G., Pleijel, H., 2022. Polycyclic aromatic hydrocarbon (PAH) accumulation in quercus palustris and pinus nigra in the urban landscape of gothenburg, Sweden. Science of The Total Environment 805, 150163.

Koblizkova, M., Genualdi, S., Lee, S.C., Harner, T., 2012a. Application of sorbent impregnated polyurethane foam (SIP) disk passive air samplers for investigating organochlorine pesticides and polybrominated diphenyl ethers at the global scale. Environmental Science & Technology 46, 391-396.

Koblizkova, M., Lee, S.C., Harner, T., 2012b. Sorbent impregnated polyurethane foam disk passive air samplers for investigating current-use pesticides at the global scale. Atmospheric Pollution Research 3, 456-462.

Kruse-Plaß, M., Hofmann, F., Wosniok, W., Schlechtriemen, U., Kohlschütter, N., 2021. Pesticides and pesticide-related products in ambient air in germany. Environmental Sciences Europe 33, 114.

Kurt-Karakus, P., Alegria, H., Birgul, A., Gungormus, E., Jantunen, L., 2018a. Organophosphate ester (OPEs) flame retardants and plasticizers in air and soil from a highly industrialized city in Turkey. Science of The Total Environment 625, 555-565.

Kurt-Karakus, P.B., Ugranli-Cicek, T., Sofuoglu, S.C., Celik, H., Gungormus, E., Gedik, K., Sofuoglu, A., Okten, H.E., Birgul, A., Alegria, H., Jones, K.C., 2018b. The first countrywide monitoring of selected POPs: Polychlorinated biphenyls (PCBs), polybrominated diphenyl ethers (PBDEs) and selected organochlorine pesticides (OCPs) in the atmosphere of Turkey. Atmospheric Environment 177, 154-165.

Lammel, G., Audy, O., Besis, A., Efstathiou, C., Eleftheriadis, K., Kohoutek, J., Kukučka, P., Mulder, M.D., Přibylová, P., Prokeš, R., Rusina, T.P., Samara, C., Sofuoglu, A., Sofuoglu, S.C., Taşdemir, Y., Vassilatou, V., Voutsa, D., Vrana, B., 2015. Air and seawater pollution and air–sea gas exchange of persistent toxic substances in the Aegean sea: Spatial trends of PAHs, PCBs, OCPs and PBDEs. Environmental Science and Pollution Research 22, 11301-11313.

Lee, S.C., Harner, T., Pozo, K., Shoeib, M., Wania, F., Muir, D.C., Barrie, L.A., Jones, K.C., 2007. Polychlorinated naphthalenes in the global atmospheric passive sampling (GAPS) study. Environmental Science & Technology 41, 2680-2687.

Lee, S.C., Sverko, E., Harner, T., Pozo, K., Barresi, E., Schachtschneider, J., Zaruk, D., DeJong, M., Narayan, J., 2016. Retrospective analysis of "new" flame retardants in the global atmosphere under the GAPS network. Environmental Pollution 217, 62-69.

Leslie, H.A., van Bavel, B., Abad, E., de Boer, J., 2013. Towards comparable POPs data worldwide with global monitoring data and analytical capacity building in Africa, central and Latin America, and the south Pacific. TrAC Trends in Analytical Chemistry 46, 85-97.

Li, B., Wu, S., Zhou, S., Wang, T., Wang, C., 2018. Spatiotemporal distribution and dynamic modeling of atmospheric gaseous polycyclic aromatic hydrocarbons in a rapidly urbanizing city: Nanjing, China. Environmental Geochemistry and Health 40, 2603-2616.

Li, J., Del Vento, S., Schuster, J., Zhang, G., Chakraborty, P., Kobara, Y., Jones, K.C., 2011. Perfluorinated compounds in the Asian atmosphere. Environmental Science & Technology 45, 7241-7248.

Li, Q., Li, J., Chaemfa, C., Zhang, G., Kobara, Y., Nam, J.-J., Jones, K.C., 2014. The impact of polybrominated diphenyl ether prohibition: A case study on the atmospheric levels in China, Japan and South Korea. Atmospheric Research 143, 57-63.

Li, Q., Li, J., Wang, Y., Xu, Y., Pan, X., Zhang, G., Luo, C., Kobara, Y., Nam, J.-J., Jones, K.C., 2012a. Atmospheric short-chain chlorinated paraffins in China, Japan, and South Korea. Environmental Science & Technology 46, 11948-11954.

Li, W.-L., Qi, H., Ma, W.-L., Liu, L.-Y., Zhang, Z., Mohammed, M.O.A., Song, W.-W., Zhang, Z., Li, Y.-F., 2015. Brominated flame retardants in Chinese air before and after the phase out of polybrominated diphenyl ethers. Atmospheric Environment 117, 156-161.

Li, Y.-F., Harner, T., Liu, L., Zhang, Z., Ren, N.-Q., Jia, H., Ma, J., Sverko, E., 2010. Polychlorinated biphenyls in global air and surface soil: Distributions, air− soil exchange, and fractionation effect. Environmental Science & Technology 44, 2784-2790.

Li, Y., Geng, D., Liu, F., Wang, T., Wang, P., Zhang, Q., Jiang, G., 2012b. Study of PCBs and PBDEs in king george island, Antarctica, using PUF passive air sampling. Atmospheric Environment 51, 140-145.

Li, Y., Zhang, Q., Ji, D., Wang, T., Wang, Y., Wang, P., Ding, L., Jiang, G., 2009. Levels and vertical distributions of PCBs, PBDEs, and OCPs in the atmospheric boundary layer: Observation from the Beijing 325-m meteorological tower. Environmental Science & Technology 43, 1030-1035.

Liang, Y., Wang, H., Yang, Q., Cao, S., Yan, C., Zhang, L., Tang, N., 2020. Spatial distribution and seasonal variations of atmospheric organophosphate esters (OPEs) in Tianjin, China based on gridded field observations. Environmental Pollution 263, 114460.

Lin, Y., Qiu, X., Ma, Y., Ma, J., Zheng, M., Shao, M., 2015. Concentrations and spatial distribution of polycyclic aromatic hydrocarbons (PAHs) and nitrated PAHs (NPAHs) in the atmosphere of North China, and the transformation from PAHs to NPAHs. Environmental Pollution 196, 164-170.

Lin, Y., Qiu, X., Zhao, Y., Ma, J., Yang, Q., Zhu, T., 2013a. Polybromobenzene pollutants in the atmosphere of North China: Levels, distribution, and sources. Environmental Science & Technology 47, 12761-12767.

Lin, Y., Zhao, Y., Qiu, X., Ma, J., Yang, Q., Shao, M., Zhu, T., 2013b. Spatial distribution of polychlorinated naphthalenes in the atmosphere across North China based on gridded field observations. Environmental Pollution 180, 27-33.

Liu, B., Xie, L., Zhang, H., Li, J., Wang, X., Dong, W., 2019. Spatial distribution of perfluorinated compounds in atmosphere of the Pearl River Delta, China. Archives of Environmental Contamination and Toxicology 77, 180-187.

Liu, H., Li, B., Qi, H., Ma, L., Xu, J., Wang, M., Ma, W., Tian, C., 2021a. Source apportionment and toxic potency of polycyclic aromatic hydrocarbons (PAHs) in the air of harbin, a cold city in northern China. Atmosphere 12, 297.

Liu, Q., Li, L., Zhang, X., Saini, A., Li, W., Hung, H., Hao, C., Li, K., Lee, P., Wentzell, J.J., 2021b. Uncovering global-scale risks from commercial chemicals in air. Nature 600, 456-461.

Liu, S., Tao, S., Liu, W., Dou, H., Liu, Y., Zhao, J., Little, M.G., Tian, Z., Wang, J., Wang, L., Gao, Y., 2008. Seasonal and spatial occurrence and distribution of atmospheric polycyclic aromatic hydrocarbons (PAHs) in rural and urban areas of the north Chinese plain. Environmental Pollution 156, 651-656.

Liu, X., Li, J., Zheng, Q., Bing, H., Zhang, R., Wang, Y., Luo, C., Liu, X., Wu, Y., Pan, S., Zhang, G., 2014. Forest filter effect versus cold trapping effect on the altitudinal distribution of PCBs: A case study of mt. Gongga, eastern Tibetan Plateau. Environmental Science & Technology 48, 14377-14385.
Liu, X., Zhang, G., Li, J., Cheng, H.-R., Qi, S.-H., Li, X.-D., Jones, K.C., 2007. Polycyclic aromatic hydrocarbons (PAHs) in the air of Chinese cities. Journal of Environmental Monitoring 9, 1092-1098.
Liu, X., Zhang, G., Li, J., Yu, L.-L., Xu, Y., Li, X.-D., Kobara, Y., Jones, K.C., 2009. Seasonal patterns and current sources of DDTs, chlordanes, hexachlorobenzene, and endosulfan in the atmosphere of 37 Chinese cities. Environmental Science & Technology 43, 1316-1321.
Llanos, Y., Cortés, S., Martínez, A., Pozo, K., Přibylová, P., Klánová, J., Jorquera, H., 2022. Local and regional sources of organochlorine pesticides in a rural zone in central Chile. Atmospheric Pollution Research 13, 101411.
Luarte, T., Gómez-Aburto, V.A., Poblete-Castro, I., Castro-Nallar, E., Huneeus, N., Molina-Montenegro, M., Egas, C., Azcune, G., Pérez-Parada, A., Lohmann, R., Bohlin-Nizzetto, P., Dachs, J., Bengtson-Nash, S., Chiang, G., Pozo, K., Galbán-Malagón, C.J., 2023. Levels of persistent organic pollutants (POPs) in the Antarctic atmosphere over time (1980 to 2021) and estimation of their atmospheric half-lives. Atmospheric Chemistry and Physics 23, 8103-8118.
Lunder Halvorsen, H., Bohlin-Nizzetto, P., Eckhardt, S., Gusev, A., Krogseth, I.S., Moeckel, C., Shatalov, V., Skogeng, L.P., Breivik, K., 2021. Main sources controlling atmospheric burdens of persistent organic pollutants on a national scale. Ecotoxicology and Environmental Safety 217, 112172.
Ma, W.-L., Liu, L.-Y., Qi, H., Sun, D.-Z., Shen, J.-M., Wang, D.-G., Li, Y.-F., 2011. Dechlorane plus in multimedia in northeastern Chinese urban region. Environment International 37, 66-70.
Mai, C., Theobald, N., Lammel, G., Hühnerfuss, H., 2013. Spatial, seasonal and vertical distributions of currently-used pesticides in the marine boundary layer of the North Sea. Atmospheric Environment 75, 92-102.
Mamontova, E.A., Mamontov, A.A., 2022. Air monitoring of polychlorinated biphenyls and organochlorine pesticides in eastern Siberia: Levels, temporal trends, and risk assessment. Atmosphere 13, 1971.
Mamontova, E.A., Tarasova, E.N., Goreglyad, A.V., Tkachenko, L.L., Mamontov, A.A., Kuzmin, M.I., 2015. Polychlorinated biphenyls and organochlorine pesticides in atmospheric air of the northern hovsgol region in 2008–2013. Doklady Earth Sciences 464, 1066-1068.
Mao, X., Yu, Z., Ding, Z., Huang, T., Ma, J., Zhang, G., Li, J., Gao, H., 2016. Sources and potential health risk of gas phase PAHs in hexi corridor, Northwest China. Environmental science and pollution research international 23, 2603-2612.
Martínez Valenzuela, C., Gavilán García, A., Conde Avila, V., Barrientos Alemán, D., Apodaca Avalos, M., Luna Valdez, J.G., Castro Carranza, G., Masías Ambríz, L.O., 2022. Applying the global monitoring plan and analysis of POPs results in atmospheric air in mexico (2017–2018). Chemosphere 303, 135154.

Mastin, J., Saini, A., Schuster, J.K., Harner, T., Dabek-Zlotorzynska, E., Celo, V., Gaga, E.O., 2023. Trace metals in global air: First results from the GAPS and GAPS megacities networks. Environmental Science & Technology 57, 14661-14673.
Meire, R.O., Lee, S.C., Targino, A.C., Torres, J.P.M., Harner, T., 2012a. Air concentrations and transport of persistent organic pollutants (POPs) in mountains of southeast and southern brazil. Atmospheric Pollution Research 3, 417-425.
Meire, R.O., Lee, S.C., Yao, Y., Targino, A.C., Torres, J.P.M., Harner, T., 2012b. Seasonal and altitudinal variations of legacy and current-use pesticides in the brazilian tropical and subtropical mountains. Atmospheric Environment 59, 108-116.
Melymuk, L., Robson, M., Helm, P.A., Diamond, M.L., 2012. PCBs, PBDEs, and PAHs in Toronto air: Spatial and seasonal trends and implications for contaminant transport. Science of The Total Environment 429, 272-280.
Messing, P.G., Farenhorst, A., Waite, D.T., Sproull, J.F., 2014. Air concentrations of currently used herbicides and legacy compounds in the Canadian prairies, subarctic, and arctic. Journal of Environmental Science and Health, Part B 49, 338-343.
Milukaitė, A., Klánová, J., Holoubek, I., Rimšelytė, I., Kvietkus, K., 2008. Persistent organic pollutants in lithuania: Assessment of air and soil contamination. Lithuanian Journal of Physics 48, 357–366-357–366.
Moreau-Guigon, E., Motelay-Massei, A., Harner, T., Pozo, K., Diamond, M., Chevreuil, M., Blanchoud, H., 2007. Vertical and temporal distribution of persistent organic pollutants in Toronto. 1. Organochlorine pesticides. Environmental Science & Technology 41, 2172-2177.
Motelay-Massei, A., Harner, T., Shoeib, M., Diamond, M., Stern, G., Rosenberg, B., 2005. Using passive air samplers to assess urban−rural trends for persistent organic pollutants and polycyclic aromatic hydrocarbons. 2. Seasonal trends for PAHs, PCBs, and organochlorine pesticides. Environmental Science & Technology 39, 5763-5773.
Moussaoui, Y., Tuduri, L., Kerchich, Y., Meklati, B.Y., Eppe, G., 2012. Atmospheric concentrations of PCDD/fs, dl-PCBs and some pesticides in northern Algeria using passive air sampling. Chemosphere 88, 270-277.
Muñoz-Arnanz, J., Roscales, J.L., Ros, M., Vicente, A., Jiménez, B., 2016. Towards the implementation of the Stockholm Convention in Spain: Five-year monitoring (2008–2013) of POPs in air based on passive sampling. Environmental Pollution 217, 107-113.
Muñoz-Arnanz, J., Roscales, J.L., Vicente, A., Ros, M., Barrios, L., Morales, L., Abad, E., Jiménez, B., 2018. Assessment of POPs in air from Spain using passive sampling from 2008 to 2015. Part II: Spatial and temporal observations of PCDD/fs and dl-PCBs. Science of The Total Environment 634, 1669-1679.
Nargis, A., Habib, A., Zhao, S., Nigar, R., Liu, X., Zhang, G., Cai, M., 2022. Monitoring of atmospheric polycyclic aromatic hydrocarbons by polyurethane foam-passive air samplers in bangladesh: Source apportionment and health risk assessment. Atmospheric Environment 289, 119346.
Nikolova, N., Lavrova, S., Petkova, P., Tsakovski, S., Pribylova, P., 2018. Passive air sampling monitoring of POPs in southeastern Europe at high mountain station beo - moussala, bulgaria. Journal of Chemical Technology and Metallurgy 53, 267-274.
Nipen, M., Vogt, R.D., Bohlin-Nizzetto, P., Borgå, K., Mwakalapa, E.B., Borgen, A.R., Jørgensen, S.J., Ntapanta, S.M., Mmochi, A.J., Schlabach, M., 2022. Spatial trends of

chlorinated paraffins and dechloranes in air and soil in a tropical urban, suburban, and rural environment. Environmental Pollution 292, 118298.
Niu, S., Chen, R., Zou, Y., Dong, L., Hai, R., Huang, Y., 2020. Spatial distribution and profile of atmospheric short-chain chlorinated paraffins in the Yangtze River Delta. Environmental Pollution 259, 113958.
Niu, S., Dong, L., Zhang, L., Zhu, C., Hai, R., Huang, Y., 2017. Temporal and spatial distribution, sources, and potential health risks of ambient polycyclic aromatic hydrocarbons in the Yangtze River Delta (yrd) of eastern China. Chemosphere 172, 72-79.
Okonski, K., Melymuk, L., Kohoutek, J., Klánová, J., 2018. Hexabromocyclododecane: Concentrations and isomer profiles from sources to environmental sinks. Environmental Science and Pollution Research 25, 36624-36635.
Osoro, E.M., Wandiga, S.O., Madadi, V.O., Abong'o, D.A., 2021. Polybrominated diphenyl ethers pollution in urban and rural settings' ambient air in kenya: An insight into concentration levels, compositional profile and seasonal variation. Africa Journal of Physical Sciences 6, 88-100.
Ouertani, N., Naouali, H., Hamouda, R., 2023. Preliminary results from the tunisian monitoring program on POPs in air under the Stockholm Convention. Chemosphere 335, 139127.
Pegoraro, C.N., Wannaz, E.D., 2019. Occurrence of persistent organic pollutants in air at different sites in the province of córdoba, argentina. Environmental Science and Pollution Research 26, 18379-18391.
Persoon, C., Peters, T.M., Kumar, N., Hornbuckle, K.C., 2010. Spatial distribution of airborne polychlorinated biphenyls in cleveland, ohio and chicago, illinois. Environmental Science & Technology 44, 2797-2802.
Peverly, A.A., Ma, Y., Venier, M., Rodenburg, Z., Spak, S.N., Hornbuckle, K.C., Hites, R.A., 2015. Variations of flame retardant, polycyclic aromatic hydrocarbon, and pesticide concentrations in chicago's atmosphere measured using passive sampling. Environmental Science & Technology 49, 5371-5379.
Pisa, L., Mhlanga, N., Mumbengengwi, P., 2020. Sources and trends of persistent organic pollutants at three passive monitoring sites in South Africa. Journal of Geoscience and Environment Protection 08, 207-218.
Platt, S.M., Hov, Ø., Berg, T., Breivik, K., Eckhardt, S., Eleftheriadis, K., Evangeliou, N., Fiebig, M., Fisher, R., Hansen, G., Hansson, H.C., Heintzenberg, J., Hermansen, O., Heslin-Rees, D., Holmén, K., Hudson, S., Kallenborn, R., Krejci, R., Krognes, T., Larssen, S., Lowry, D., Lund Myhre, C., Lunder, C., Nisbet, E., Nizzetto, P.B., Park, K.T., Pedersen, C.A., Aspmo Pfaffhuber, K., Röckmann, T., Schmidbauer, N., Solberg, S., Stohl, A., Ström, J., Svendby, T., Tunved, P., Tørnkvist, K., van der Veen, C., Vratolis, S., Yoon, Y.J., Yttri, K.E., Zieger, P., Aas, W., Tørseth, K., 2022. Atmospheric composition in the European Arctic and 30 years of the zeppelin observatory, Ny-Ålesund. Atmospheric Chemistry and Physics 22, 3321-3369.
Pokhrel, B., Gong, P., Wang, X., Khanal, S.N., Ren, J., Wang, C., Gao, S., Yao, T., 2018a. Atmospheric organochlorine pesticides and polychlorinated biphenyls in urban areas of Nepal: Spatial variation, sources, temporal trends, and long-range transport potential. Atmospheric Chemistry and Physics 18, 1325-1336.

Pokhrel, B., Gong, P., Wang, X., Wang, C., Gao, S., 2018b. Polycyclic aromatic hydrocarbons in the urban atmosphere of Nepal: Distribution, sources, seasonal trends, and cancer risk. Science of The Total Environment 618, 1583-1590.

Pozo, K., Estellano, V.H., Harner, T., Diaz-Robles, L., Cereceda-Balic, F., Etcharren, P., Pozo, K., Vidal, V., Guerrero, F., Vergara-Fernández, A., 2015. Assessing polycyclic aromatic hydrocarbons (PAHs) using passive air sampling in the atmosphere of one of the most wood-smoke-polluted cities in Chile: The case study of Temuco. Chemosphere 134, 475-481.

Pozo, K., Gómez, V., Přibylová, P., Lammel, G., Klánová, J., Rudolph, A., Ahumada, R., 2022. Multicompartmental analysis of POPs and PAHs in concepción bay, central Chile: Part I – levels and patterns after the 2010 tsunami. Marine Pollution Bulletin 174, 113144.

Pozo, K., Harner, T., Lee, S.C., Sinha, R.K., Sengupta, B., Loewen, M., Geethalakshmi, V., Kannan, K., Volpi, V., 2011. Assessing seasonal and spatial trends of persistent organic pollutants (POPs) in Indian agricultural regions using PUF disk passive air samplers. Environmental Pollution 159, 646-653.

Pozo, K., Harner, T., Lee, S.C., Wania, F., Muir, D.C., Jones, K.C., 2009. Seasonally resolved concentrations of persistent organic pollutants in the global atmosphere from the first year of the GAPS study. Environmental Science & Technology 43, 796-803.

Pozo, K., Harner, T., Rudolph, A., Oyola, G., Estellano, V.H., Ahumada-Rudolph, R., Garrido, M., Pozo, K., Mabilia, R., Focardi, S., 2012. Survey of persistent organic pollutants (POPs) and polycyclic aromatic hydrocarbons (PAHs) in the atmosphere of rural, urban and industrial areas of concepcion, Chile, using passive air samplers. Atmospheric Pollution Research 3, 426-434.

Pozo, K., Harner, T., Shoeib, M., Urrutia, R., Barra, R., Parra, O., Focardi, S., 2004. Passive-sampler derived air concentrations of persistent organic pollutants on a north− south transect in Chile. Environmental Science & Technology 38, 6529-6537.

Pozo, K., Harner, T., Wania, F., Muir, D.C., Jones, K.C., Barrie, L.A., 2006. Toward a global network for persistent organic pollutants in air: Results from the GAPS study. Environmental Science & Technology 40, 4867-4873.

Pozo, K., Llanos, Y., Estellano, V.H., Cortés, S., Jorquera, H., Gerli, L., Pozo, K., Encina, F., Palma, R., Focardi, S., 2016a. Occurrence of chlorpyrifos in the atmosphere of the araucanía region in Chile using polyurethane foam-based passive air samplers. Atmospheric Pollution Research 7, 706-710.

Pozo, K., Martellini, T., Corsolini, S., Harner, T., Estellano, V., Kukučka, P., Mulder, M.D., Lammel, G., Cincinelli, A., 2017a. Persistent organic pollutants (POPs) in the atmosphere of coastal areas of the ross sea, Antarctica: Indications for long-term downward trends. Chemosphere 178, 458-465.

Pozo, K., Oyola, G., Estellano, V.H., Harner, T., Rudolph, A., Prybilova, P., Kukucka, P., Audi, O., Klánová, J., Metzdorff, A., Focardi, S., 2017b. Persistent organic pollutants (POPs) in the atmosphere of three chilean cities using passive air samplers. Science of The Total Environment 586, 107-114.

Pozo, K., Palmeri, M., Palmeri, V., Estellano, V.H., Mulder, M.D., Efstathiou, C.I., Sará, G.L., Romeo, T., Lammel, G., Focardi, S., 2016b. Assessing persistent organic pollutants (POPs) in the sicily island atmosphere, Mediterranean, using PUF disk passive air samplers. Environmental science and pollution research international 23, 20796-20804.

Pozo, K., Sarkar, S.K., Estellano, V.H., Mitra, S., Audi, O., Kukucka, P., Přibylová, P., Klánová, J., Corsolini, S., 2017c. Passive air sampling of persistent organic pollutants (POPs) and emerging compounds in kolkata megacity and rural mangrove wetland sundarban in India: An approach to regional monitoring. Chemosphere 168, 1430-1438.
Prats, R.M., van Drooge, B.L., Fernández, P., Marco, E., Grimalt, J.O., 2021. Changes in urban gas-phase persistent organic pollutants during the covid-19 lockdown in barcelona. Frontiers in Environmental Science Volume 9 - 2021.
Pribylova, P., Kares, R., Boruvkova, J., Cupr, P., Prokes, R., Kohoutek, J., Holoubek, I., Klanova, J., 2012. Levels of persistent organic pollutants and polycyclic aromatic hydrocarbons in ambient air of central and eastern Europe. Atmospheric Pollution Research 3, 494-505.
Prithiviraj, B., Taneja, A., Chakraborty, P., 2021. Atmospheric polychlorinated biphenyls in a non-metropolitan city in northern India: Levels, seasonality and sources. Chemosphere 263, 127700.
Qi, A., Wang, P., Lv, J., Zhao, T., Huang, Q., Wang, Y., Zhang, X., Wang, M., Xiao, Y., Yang, L., Ji, Y., Wang, W., 2023. Distributions of PAHs, NPAHs, OPAHs, BrPAHs, and ClPAHs in air, bulk deposition, soil, and water in the Shandong Peninsula, China: Urban-rural gradient, interface exchange, and long-range transport. Ecotoxicology and Environmental Safety 265, 115494.
Qu, C., Albanese, S., Cicchella, D., Fortelli, A., Hope, D., Esposito, M., Cerino, P., Pizzolante, A., Qi, S., De Vivo, B., Lima, A., 2022. The contribution of persistent organic pollutants to the environmental changes in campania region, italy: Results from the campania trasparente project. Journal of Geochemical Exploration 241, 107071.
Qu, C., Albanese, S., Lima, A., Hope, D., Pond, P., Fortelli, A., Romano, N., Cerino, P., Pizzolante, A., De Vivo, B., 2019. The occurrence of OCPs, PCBs, and PAHs in the soil, air, and bulk deposition of the naples metropolitan area, southern italy: Implications for sources and environmental processes. Environment International 124, 89-97.
Qu, C., De Vivo, B., Albanese, S., Fortelli, A., Scafetta, N., Li, J., Hope, D., Cerino, P., Pizzolante, A., Qi, S., Lima, A., 2021. High spatial resolution measurements of passive-sampler derived air concentrations of persistent organic pollutants in the campania region, italy: Implications for source identification and risk analysis. Environmental Pollution 286, 117248.
Qu, C., Xing, X., Albanese, S., Doherty, A., Huang, H., Lima, A., Qi, S., De Vivo, B., 2015. Spatial and seasonal variations of atmospheric organochlorine pesticides along the plain-mountain transect in central China: Regional source vs. Long-range transport and air–soil exchange. Atmospheric Environment 122, 31-40.
Rauert, C., Harner, T., Schuster, J.K., Eng, A., Fillmann, G., Castillo, L.E., Fentanes, O., Ibarra, M.V., Miglioranza, K.S.B., Rivadeneira, I.M., Pozo, K., Aristizábal Zuluaga, B.H., 2018a. Air monitoring of new and legacy POPs in the Group of Latin America and Caribbean (GRULAC) region. Environmental Pollution 243, 1252-1262.
Rauert, C., Harner, T., Schuster, J.K., Eng, A., Fillmann, G., Castillo, L.E., Fentanes, O., Villa Ibarra, M., Miglioranza, K.S.B., Moreno Rivadeneira, I., Pozo, K., Aristizábal Zuluaga, B.H., 2018b. Atmospheric concentrations of new persistent organic pollutants and emerging chemicals of concern in the Group of Latin America and Caribbean (GRULAC) region. Environmental Science & Technology 52, 7240-7249.

Rauert, C., Harner, T., Schuster, J.K., Quinto, K., Fillmann, G., Castillo, L.E., Fentanes, O., Ibarra, M.V., Miglioranza, K.S., Rivadeneira, I.M., 2016. Towards a regional passive air sampling network and strategy for new POPs in the GRULAC region: Perspectives from the GAPS network and first results for organophosphorus flame retardants. Science of The Total Environment 573, 1294-1302.
Rauert, C., Schuster, J.K., Eng, A., Harner, T., 2018c. Global atmospheric concentrations of brominated and chlorinated flame retardants and organophosphate esters. Environmental Science & Technology 52, 2777-2789.
Rauert, C., Shoieb, M., Schuster, J.K., Eng, A., Harner, T., 2018d. Atmospheric concentrations and trends of poly- and perfluoroalkyl substances (PFAS) and volatile methyl siloxanes (VMS) over 7 years of sampling in the global atmospheric passive sampling (GAPS) network. Environmental Pollution 238, 94-102.
Ren, J., Wang, X., Xue, Y., Gong, P., Joswiak, D.R., Xu, B., Yao, T., 2014. Persistent organic pollutants in mountain air of the southeastern Tibetan Plateau: Seasonal variations and implications for regional cycling. Environmental Pollution 194, 210-216.
Ren, N., Sverko, E., Li, Y.-F., Zhang, Z., Harner, T., Wang, D., Wan, X., McCarry, B.E., 2008. Levels and isomer profiles of dechlorane plus in Chinese air. Environmental Science & Technology 42, 6476-6480.
Riaz, R., Malik, R.N., de Wit, C.A., 2021. Soil-air partitioning of semivolatile organic compounds in the lesser himalaya region: Influence of soil organic matter, atmospheric transport processes and secondary emissions. Environmental Pollution 291, 118006.
Roots, O., Lukki, T., Přibylová, P., Borůvková, J., Kukučka, P., Audy, O., Kalina, J., Klánová, J., Holoubek, I., Sweetman, A., 2015. Measurements of persistent organic pollutants in estonian ambient air (1990-2013). Proceedings of the Estonian Academy of Sciences 64.
Roots, O., Roose, A., Kull, A., Holoubek, I., Cupr, P., Klanova, J., 2010. Distribution pattern of PCBs, HCB and PeCB using passive air and soil sampling in estonia. Environmental Science and Pollution Research 17, 740-749.
Roots, O., Sweetman, A., 2007. Passive air sampling of persistent organic pollutants in two estonian air monitoring stations. Oil Shale 24.
Rosa, A.H., Stubbings, W.A., Akinrinade, O.E., Jeunon Gontijo, E.S., Harrad, S., 2024. Neural network for evaluation of the impact of the UK covid-19 national lockdown on atmospheric concentrations of PAHs and PBDEs. Environmental Pollution 341, 122794.
Roscales, J.L., Muñoz-Arnanz, J., Ros, M., Vicente, A., Barrios, L., Jiménez, B., 2018. Assessment of POPs in air from Spain using passive sampling from 2008 to 2015. Part I: Spatial and temporal observations of PBDEs. Science of The Total Environment 634, 1657-1668.
Ruggeri, M.F., Lana, N.B., Altamirano, J.C., Puliafito, S.E., 2020. Spatial distribution, patterns and source contributions of POPs in the atmosphere of great mendoza using the wrf/calmet/calpuff modelling system. Emerging Contaminants 6, 103-113.
Saini, A., Chinnadurai, S., Schuster, J.K., Eng, A., Harner, T., 2023. Per- and polyfluoroalkyl substances and volatile methyl siloxanes in global air: Spatial and temporal trends. Environmental Pollution 323, 121291.
Saini, A., Harner, T., Chinnadhurai, S., Schuster, J.K., Yates, A., Sweetman, A., Aristizabal-Zuluaga, B.H., Jiménez, B., Manzano, C.A., Gaga, E.O., 2020. GAPS-megacities: A new global platform for investigating persistent organic pollutants and chemicals of emerging concern in urban air. Environmental Pollution 267, 115416.

Sampath, S., Selvaraj, K.K., Shanmugam, G., Krishnamoorthy, V., Chakraborty, P., Ramaswamy, B.R., 2017. Evaluating spatial distribution and seasonal variation of phthalates using passive air sampling in southern India. Environmental Pollution 221, 407-417.
Sampath, S., Shanmugam, G., Selvaraj, K.K., Ramaswamy, B.R., 2015. Spatio-temporal distribution of polycyclic aromatic hydrocarbons (PAHs) in atmospheric air of tamil nadu, India, and human health risk assessment. Environmental Forensics 16, 76-87.
Sáňka, O., Melymuk, L., Čupr, P., Dvorská, A., Klánová, J., 2014. Dispersion modeling of selected PAHs in urban air: A new approach combining dispersion model with gis and passive air sampling. Atmospheric Environment 96, 88-95.
Sanli, G., Celik, S., Joubi, V., Tasdemir, Y., 2023. Concentrations, phase exchanges and source apportionment of polycyclic aromatic hydrocarbons (PAHs) in Bursa-Turkey. Environmental Research 232, 116344.
Sanli, G.E., Tasdemir, Y., 2022. Accumulations and temporal trends of polychlorinated biphenyls (PCBs) in olive tree components. Environmental Geochemistry and Health 44, 2577-2594.
Santiago, E.C., Cayetano, M.G., 2007. Polycyclic aromatic hydrocarbons in ambient air in the Philippines derived from passive sampler with polyurethane foam disk. Atmospheric Environment 41, 4138-4147.
Santiago, E.C., Cayetano, M.G., 2011. Organochlorine pesticides in ambient air in selected urban and rural residential areas in the Philippines derived from passive samplers with polyurethane disks. Bulletin of Environmental Contamination and Toxicology 86, 50-55.
Sari, M.F., Córdova Del Águila, D.A., Tasdemir, Y., Esen, F., 2020. Atmospheric concentration, source identification, and health risk assessment of persistent organic pollutants (POPs) in two countries: Peru and Turkey. Environmental Monitoring and Assessment 192, 655.
Sari, M.F., Esen, F., 2022. Atmospheric concentration, spatial variations, and source identification of persistent organic pollutants in urban and semi-urban areas using passive air samplers in Bursa, Turkey. Environmental Science and Pollution Research 29, 32082-32092.
Sari, M.F., Esen, F., Cetin, B., 2023. Concentration levels, spatial variations and exchanges of polychlorinated biphenyls (PCBs) in ambient air, surface water and sediment in Bursa, Türkiye. Science of The Total Environment 880, 163224.
Sari, M.F., Esen, F., Tasdemir, Y., 2021. Levels of polychlorinated biphenyls (PCBs) in honeybees and bee products and their evaluation with ambient air concentrations. Atmospheric Environment 244, 117903.
Sau, T.K., 2023. Concentrations of PCDD/fs and dl-PCBs in ambient air in hanoi, Vietnam, between 2017 and 2021, and health risk assessments. Environmental Science and Pollution Research 30, 98440-98451.
Schuster, J.K., Harner, T., Eng, A., Rauert, C., Su, K., Hornbuckle, K.C., Johnson, C.W., 2021a. Tracking POPs in global air from the first 10 years of the GAPS network (2005 to 2014). Environmental Science & Technology 55, 9479-9488.
Schuster, J.K., Harner, T., Fillmann, G., Ahrens, L., Altamirano, J.C., Aristizábal, B., Bastos, W., Castillo, L.E., Cortés, J., Fentanes, O., Gusev, A., Hernandez, M., Ibarra, M.n.V., Lana, N.B., Lee, S.C., Martínez, A.P., Miglioranza, K.S.B., Puerta, A.P., Segovia, F., Siu, M., Tominaga, M.Y., 2015. Assessing polychlorinated dibenzo-p-dioxins and

polychlorinated dibenzofurans in air across Latin American countries using polyurethane foam disk passive air samplers. Environmental Science & Technology 49, 3680-3686.

Schuster, J.K., Harner, T., Rauert, C., 2023. Impacts of proximity to primary source areas on concentrations of POPs at global sampling stations estimated from land cover information. ACS Omega 8, 36016-36024.

Schuster, J.K., Harner, T., Sverko, E., 2021b. Dechlorane plus in the global atmosphere. Environmental Science & Technology Letters 8, 39-45.

Shahin, N., Hussein, A., Reiman, J., Alkhalil, S., Salman, M., 2017. PCDD/PCDF and pl-PCBs concentration in ambient atmosphere in the city of tulkarm using passive air sampler. Environment and Pollution 6, 34.

Shen, P., Song, X., Li, N., Zhao, C., 2023. Concentrations and distributions of fluorotelomer alcohols and perfluoroalkane sulfonamido substances in the atmosphere in the Pearl River Delta, China. Journal of Environmental Science and Health, Part A 58, 183-190.

Skogeng, L.P., Lunder Halvorsen, H., Breivik, K., Eckhardt, S., Herzke, D., Moeckel, C., Krogseth, I.S., 2023. Spatial distribution of dechlorane plus and dechlorane related compounds in European background air. Frontiers in Environmental Science Volume 10 - 2022.

Sohail, M., Musstjab Akber Shah Eqani, S.A., Ilyas, S., Bokhari, H., Ali, N., Podgorski, J.E., Muhammad, S., Adelman, D., Lohmann, R., 2023. Gaseous and soil OCPs and PCBs along the Indus river, Pakistan: Spatial patterns and air–soil gradients. Environmental Science: Processes & Impacts 25, 531-541.

Srimurali, S., Govindaraj, S., Krishna Kumar, S., Babu Rajendran, R., 2015. Distribution of organochlorine pesticides in atmospheric air of tamilnadu, southern India. International Journal of Environmental Science and Technology 12, 1957-1964.

Stafilov, T., Škrbić, B., Klánová, J., Čupr, P., Holoubek, I., Kočov, M., Đurišić-Mladenović, N., 2011. Chemometric assessment of the semivolatile organic contaminants content in the atmosphere of the selected sites in the republic of macedonia. Journal of Chemometrics 25, 262-274.

Sun, R., Wang, X., Tian, C., Zong, Z., Ma, W., Zhao, S., Wang, Y., Tang, J., Cui, S., Li, J., Zhang, G., 2022. Exploring source footprint of organophosphate esters in the Bohai Sea, China: Insight from temporal and spatial variabilities in the atmosphere from june 2014 to may 2019. Environment International 159, 107044.

Sun, W., Liu, H., Zhang, J., Zhang, B., Qu, C., 2021. Status, sources, and health risk of hexachlorocyclohexanes in the air of the rural region of zhangzhou, southeast China. Bulletin of Environmental Contamination and Toxicology 106, 676-682.

Surenjav, E., Fiedler, H., 2023. POPs in the mongolian environment. Emerging Contaminants 9, 100251.

Syed, J.H., Malik, R.N., Li, J., Zhang, G., Jones, K.C., 2013a. Levels, distribution and air–soil exchange fluxes of polychlorinated biphenyls (PCBs) in the environment of Punjab province, Pakistan. Ecotoxicology and Environmental Safety 97, 189-195.

Syed, J.H., Malik, R.N., Liu, D., Xu, Y., Wang, Y., Li, J., Zhang, G., Jones, K.C., 2013b. Organochlorine pesticides in air and soil and estimated air–soil exchange in Punjab, Pakistan. Science of The Total Environment 444, 491-497.

Tahir, A., Abbasi, N.A., He, C., Ahmad, S.R., 2024. Spatial distribution and air-soil exchange of short and medium chain chlorinated paraffins in Lahore, Pakistan. Science of The Total Environment 953, 176054.

Tao, S., Liu, Y., Xu, W., Lang, C., Liu, S., Dou, H., Liu, W., 2007. Calibration of a passive sampler for both gaseous and particulate phase polycyclic aromatic hydrocarbons. Environmental Science & Technology 41, 568-573.

Thang, P.Q., Kim, S.-J., Lee, S.-J., Kim, C.H., Lim, H.-J., Lee, S.-B., Kim, J.Y., Vuong, Q.T., Choi, S.-D., 2020. Monitoring of polycyclic aromatic hydrocarbons using passive air samplers in Seoul, South Korea: Spatial distribution, seasonal variation, and source identification. Atmospheric Environment 229, 117460.

Tian, Y., Nie, Z., Tian, S., Liu, F., He, J., Yang, Y., Wang, X., Die, Q., Fang, Y., Huang, Q., 2015. Passive air sampling for determining the levels of ambient PCDD/fs and their seasonal and spatial variations and inhalation risk in Shanghai, China. Environmental Science and Pollution Research 22, 13243-13250.

Tombesi, N., Pozo, K., Harner, T., 2014. Persistent organic pollutants (POPs) in the atmosphere of agricultural and urban areas in the province of buenos aires in argentina using PUF disk passive air samplers. Atmospheric Pollution Research 5, 170-178.

Tominaga, M.Y., Silva, C.R., Melo, J.P., Niwa, N.A., Plascak, D., Souza, C.A.M., Sato, M.I.Z., 2016. PCDD, PCDF, dl-PCB and organochlorine pesticides monitoring in são paulo city using passive air sampler as part of the global monitoring plan. Science of The Total Environment 571, 323-331.

Tomsho, K.S., Basra, K., Rubin, S.M., Miller, C.B., Juang, R., Broude, S., Martinez, A., Hornbuckle, K.C., Heiger-Bernays, W., Scammell, M.K., 2018. Community reporting of ambient air polychlorinated biphenyl concentrations near a superfund site. Environmental Science and Pollution Research 25, 16389-16400.

Tong, L., Peng, C.-H., Huang, Z.-W., Zhang, J.-J., Dai, X.-R., Xiao, H., Xu, N.-B., He, J., 2019. Identifying the pollution characteristics of atmospheric polycyclic aromatic hydrocarbons associated with functional districts in ningbo, China. Bulletin of Environmental Contamination and Toxicology 103, 34-40.

Ukpebor, J., Omoruyi, C., Omonmhenle, S., Imhontu, M., Oghoje, S., Isara, A., Ukpebor, E., 2023. Concentrations of PAHs from urban and rural areas in southern nigeria using polyurethane foams (PUF) passive samplers: Health impact and cancer risk assessment. Scientific African 22, e01976.

Vakarelska, E., Nedyalkova, M., Nikolova, N., Angelov, C., Tonev, D., Prybilova, P., Klanova, J., Simeonov, V., 2021. Tracing the movement of persistent organic pollutants at a high-mountain sampling site by chemometric assessment. Journal of Environmental Science and Health, Part A 56, 1041-1049.

Valdivia, M.J., Sánchez, S., Saa, J., Bastías, R., Higuera, G., Fleming, Z.L., Manzano, C.A., Alcamán-Arias, M.E., 2024. Emission of polycyclic aromatic hydrocarbons in the north-west Antarctic peninsula region. Environmental Science and Pollution Research 31, 64950-64960.

Van Vu, T., Van Tran, C., Hoang, N., Do, H.T., Van Le, C., Thang, P.Q., Minh, T.B., Tran, T.M., 2024. Distributions of polycyclic aromatic hydrocarbons in ambient air samples from hanoi urban areas, Vietnam, and its implications for inhalation exposure. Environmental Science and Pollution Research 31, 55132-55144.

Vardar, N., Chemseddine, Z., Santos, J., 2013. Effect of ambient temperature on PUF passive samplers and PAHs distribution in Puerto Rico. Computational Water, Energy, and Environmental Engineering 2, 41-45.

Vasiljevic, T., Su, K., Harner, T., 2021. A first look at atmospheric concentrations and temporal trends of phthalates in distinct urban sectors of the greater Toronto area. Atmospheric Pollution Research 12, 173-182.
Veludo, A.F., Martins Figueiredo, D., Degrendele, C., Masinyana, L., Curchod, L., Kohoutek, J., Kukučka, P., Martiník, J., Přibylová, P., Klánová, J., Dalvie, M.A., Röösli, M., Fuhrimann, S., 2022. Seasonal variations in air concentrations of 27 organochlorine pesticides (OCPs) and 25 current-use pesticides (CUPs) across three agricultural areas of South Africa. Chemosphere 289, 133162.
Vlahos, P., Shipley, E.R., Rauert, C., Tung, P., Chandrajith, R., Wickramarathna, S., Harner, T., 2023. A hitchhiker's guide to persistent organic pollutants (POPs) monitoring in air: Establishing a baseline network in Sri Lanka. Atmospheric Pollution Research 14, 101901.
Wang, C., Wang, X., Ren, J., Gong, P., Yao, T., 2017. Using a passive air sampler to monitor air-soil exchange of organochlorine pesticides in the pasture of the central Tibetan Plateau. Science of The Total Environment 580, 958-965.
Wang, F., Zhao, D., Lu, P., Zhang, D., Guo, Z., Rose, N.L., Zhang, G., 2024a. Air–plant interaction and air–soil exchange of polycyclic aromatic hydrocarbons in a large human-influenced reservoir in southwest China. Environmental Pollution 355, 124216.
Wang, J., Guo, L., Li, J., Zhang, G., Lee, C.S.L., Li, X., Jones, K.C., Xiang, Y., Zhong, L., 2007. Passive air sampling of DDT, chlordane and HCB in the Pearl River Delta, South China: Implications to regional sources. Journal of Environmental Monitoring 9, 582-588.
Wang, Q., Ruan, Y., Zhao, Z., Zhang, L., Hua, X., Jin, L., Chen, H., Wang, Y., Yao, Y., Lam, P.K.S., Zhu, L., Sun, H., 2022a. Per- and polyfluoroalkyl substances (PFAS) in the three-north shelter forest in northern China: First survey on the effects of forests on the behavior of PFAS. Journal of Hazardous Materials 427, 128157.
Wang, S., Lin, X., Li, Q., Liu, C., Li, Y., Wang, X., 2022b. Neutral and ionizable per-and polyfluoroalkyl substances in the urban atmosphere: Occurrence, sources and transport. Science of The Total Environment 823, 153794.
Wang, S., Steiniche, T., Romanak, K.A., Johnson, E., Quirós, R., Mutegeki, R., Wasserman, M.D., Venier, M., 2019. Atmospheric occurrence of legacy pesticides, current use pesticides, and flame retardants in and around protected areas in Costa Rica and uganda. Environmental Science & Technology 53, 6171-6181.
Wang, T., Guan, Y., Zeng, Y., Yang, P., Xiang, K., Chen, S., 2024b. Spatiotemporal patterns and deposition of organophosphate esters (OPEs) in air, foliage and litter in a subtropical forest of South China. Environmental Research 252, 119059.
Wang, T., Xiang, K., Zeng, Y., Gu, H., Guan, Y., Chen, S., 2023. Polycyclic aromatic hydrocarbons (PAHs) in air, foliage, and litter in a subtropical forest: Spatioseasonal variations, partitioning, and litter-PAH degradation. Environmental Pollution 328, 121587.
Wang, W., Wang, Y., Zhang, R., Wang, S., Wei, C., Chaemfa, C., Li, J., Zhang, G., Yu, K., 2016. Seasonal characteristics and current sources of OCPs and PCBs and enantiomeric signatures of chiral OCPs in the atmosphere of Vietnam. Science of The Total Environment 542, 777-786.
Wang, X., Schuster, J., Jones, K.C., Gong, P., 2018a. Occurrence and spatial distribution of neutral perfluoroalkyl substances and cyclic volatile methylsiloxanes in the atmosphere of the Tibetan Plateau. Atmospheric Chemistry and Physics 18, 8745-8755.

Wang, X.Y., Li, Q.B., Luo, Y.M., Ding, Q., Xi, L.M., Ma, J.M., Li, Y., Liu, Y.P., Cheng, C.L., 2010. Characteristics and sources of atmospheric polycyclic aromatic hydrocarbons (PAHs) in Shanghai, China. Environmental Monitoring and Assessment 165, 295-305.
Wang, Y., Bao, M., Zhang, Y., Tan, F., Zhao, H., Zhang, Q., Li, Q., 2020a. Polycyclic aromatic hydrocarbons in the atmosphere and soils of Dalian, China: Source, urban-rural gradient, and air-soil exchange. Chemosphere 244, 125518.
Wang, Y., Li, J., Cheng, Z., Li, Q., Pan, X., Zhang, R., Liu, D., Luo, C., Liu, X., Katsoyiannis, A., Zhang, G., 2013. Short- and medium-chain chlorinated paraffins in air and soil of subtropical terrestrial environment in the Pearl River Delta, South China: Distribution, composition, atmospheric deposition fluxes, and environmental fate. Environmental Science & Technology 47, 2679-2687.
Wang, Y., Li, Q., Xu, Y., Luo, C., Liu, X., Li, J., Zhang, G., 2012. Improved correction method for using passive air samplers to assess the distribution of PCNs in the Dongjiang River basin of the Pearl River Delta, South China. Atmospheric Environment 54, 700-705.
Wang, Y., Li, Z., Tan, F., Xu, Y., Zhao, H., Chen, J., 2020b. Occurrence and air-soil exchange of organophosphate flame retardants in the air and soil of Dalian, China. Environmental Pollution 265, 114850.
Wang, Y., Wu, X., Zhang, Q., Zhao, H., Hou, M., Xie, Q., Chen, J., 2018b. Occurrence, distribution, and air-water exchange of organophosphorus flame retardants in a typical coastal area of China. Chemosphere 211, 335-344.
White, K.B., Kalina, J., Scheringer, M., Přibylová, P., Kukučka, P., Kohoutek, J., Prokeš, R., Klánová, J., 2021a. Temporal trends of persistent organic pollutants across Africa after a decade of MONET passive air sampling. Environmental Science & Technology 55, 9413-9424.
White, K.B., Kalina, J.i., Scheringer, M., Přibylová, P., Kukučka, P., Kohoutek, J.i., Prokeš, R., Klánová, J., 2023. Spatial and temporal trends of persistent organic pollutants across Europe after 15 years of MONET passive air sampling. Environmental Science & Technology 57, 11583-11594.
White, K.B., Sáňka, O., Melymuk, L., Přibylová, P., Klánová, J., 2021b. Application of land use regression modelling to describe atmospheric levels of semivolatile organic compounds on a national scale. Science of The Total Environment 793, 148520.
Wong, F., Alegria, H.A., Bidleman, T.F., Alvarado, V., Angeles, F., Galarza, A.Á., Bandala, E.R., Hinojosa, I.d.l.C., Estrada, I.G., Reyes, G.G., 2009. Passive air sampling of organochlorine pesticides in mexico. Environmental Science & Technology 43, 704-710.
Wu, D., Liu, H., Wang, Z., Zhang, J., Zhan, C., Liu, S., Liu, T., Zheng, J., Yao, R., Cao, J., 2020. Atmospheric concentrations and air–soil exchange of polycyclic aromatic hydrocarbons (PAHs) in typical urban–rural fringe of Wuhan–Ezhou region, Central China. Bulletin of Environmental Contamination and Toxicology 104, 96-106.
Wu, X., Davie-Martin, C.L., Steinlin, C., Hageman, K.J., Cullen, N.J., Bogdal, C., 2017. Understanding and predicting the fate of semivolatile organic pesticides in a glacier-fed lake using a multimedia chemical fate model. Environmental Science & Technology 51, 11752-11760.
Wu, X., Wang, Y., Zhang, Q., Zhao, H., Yang, Y., Zhang, Y., Xie, Q., Chen, J., 2019. Seasonal variation, air-water exchange, and multivariate source apportionment of polycyclic aromatic hydrocarbons in the coastal area of Dalian, China. Environmental Pollution 244, 405-413.

Wu, Z., Lin, T., Sun, H., Li, R., Liu, X., Guo, Z., Ma, X., Yao, Z., 2023. Polycyclic aromatic hydrocarbons in fildes peninsula, maritime Antarctica: Effects of human disturbance. Environmental Pollution 318, 120768.
Xia, C., Capozzi, S.L., Romanak, K.A., Lehman, D.C., Dove, A., Richardson, V., Greenberg, T., McGoldrick, D., Venier, M., 2024. The ins and outs of per- and polyfluoroalkyl substances in the Great Lakes: The role of atmospheric deposition. Environmental Science & Technology 58, 9303-9313.
Xia, Z., Duan, X., Tao, S., Qiu, W., Liu, D., Wang, Y., Wei, S., Wang, B., Jiang, Q., Lu, B., Song, Y., Hu, X., 2013. Pollution level, inhalation exposure and lung cancer risk of ambient atmospheric polycyclic aromatic hydrocarbons (PAHs) in Taiyuan, China. Environmental Pollution 173, 150-156.
Xu, C., Niu, L., Zou, D., Zhu, S., Liu, W., 2019. Congener-specific composition of polychlorinated biphenyls (PCBs) in soil-air partitioning and the associated health risks. Science of The Total Environment 684, 486-495.
Xu, D., Zhang, Z., Jiang, Q., Li, Y., Chai, Z., 2008. Hybrid naa method for assessment of the levels of organic halogen compounds in the atmosphere in China. Journal of Radioanalytical and Nuclear Chemistry 278, 241-245.
Xu, Y., Li, J., Chakraborty, P., Syed, J.H., Malik, R.N., Wang, Y., Tian, C., Luo, C., Zhang, G., Jones, K.C., 2014. Atmospheric polychlorinated naphthalenes (PCNs) in India and Pakistan. Science of The Total Environment 466, 1030-1036.
Yadav, I.C., Devi, N.L., Li, J., Zhang, G., 2018. Altitudinal and spatial variations of polycyclic aromatic hydrocarbons in Nepal: Implications on source apportionment and risk assessment. Chemosphere 198, 386-396.
Yadav, I.C., Devi, N.L., Li, J., Zhang, G., Breivik, K., 2017. Possible emissions of POPs in plain and hilly areas of Nepal: Implications for source apportionment and health risk assessment. Environmental Pollution 220, 1289-1300.
Yao, Y., Harner, T., Ma, J., Tuduri, L., Blanchard, P., 2007. Sources and occurrence of dacthal in the Canadian atmosphere. Environmental Science & Technology 41, 688-694.
Yoonki, M., Jongwon, H., Meehye, L., 2014. Determination of toxic congeners of 17 PCDDs/pcdfs and 12 dl-PCBs using polyurethane foam passive air samplers in ten cities around Seoul. Science of The Total Environment 491-492, 17-27.
Zaller, J.G., Kruse-Plaß, M., Schlechtriemen, U., Gruber, E., Peer, M., Nadeem, I., Formayer, H., Hutter, H.-P., Landler, L., 2023. Unexpected air pollutants with potential human health hazards: Nitrification inhibitors, biocides, and persistent organic substances. Science of The Total Environment 862, 160643.
Zencak, Z., Klanova, J., Holoubek, I., Gustafsson, O., 2007. Source apportionment of atmospheric PAHs in the western Balkans by natural abundance radiocarbon analysis. Environmental Science & Technology 41, 3850-3855.
Zhang, G., Chakraborty, P., Li, J., Sampathkumar, P., Balasubramanian, T., Kathiresan, K., Takahashi, S., Subramanian, A., Tanabe, S., Jones, K.C., 2008a. Passive atmospheric sampling of organochlorine pesticides, polychlorinated biphenyls, and polybrominated diphenyl ethers in urban, rural, and wetland sites along the coastal length of India. Environmental Science & Technology 42, 8218-8223.
Zhang, J., Wang, X., Gong, P., Wang, C., Sun, D., 2018a. Seasonal variation and source analysis of persistent organic pollutants in the atmosphere over the western Tibetan Plateau. Environmental Science and Pollution Research 25, 24052-24063.

Zhang, J., Zhao, J., Cai, J., Gao, S., Li, J., Zeng, X., Yu, Z., 2018b. Spatial distribution and source apportionment of atmospheric polycyclic aromatic hydrocarbons in the Pearl River Delta, China. Atmospheric Pollution Research 9, 887-893.
Zhang, J., Zhao, J., Cai, J., Zeng, X., Li, J., Gao, S., Yu, Z., 2018c. Distribution of polybrominated diphenyl ethers in the atmosphere of the Pearl River Delta region, South China. Environmental Science and Pollution Research 25, 27013-27020.
Zhang, L., Dong, L., Huang, Y., Shi, S., Yang, W., Zhou, L., 2016a. Seasonality in polybrominated diphenyl ether concentrations in the atmosphere of the Yangtze River Delta, China. Chemosphere 150, 438-444.
Zhang, L., Dong, L., Yang, W., Zhou, L., Shi, S., Zhang, X., Niu, S., Li, L., Wu, Z., Huang, Y., 2013. Passive air sampling of organochlorine pesticides and polychlorinated biphenyls in the Yangtze River Delta, China: Concentrations, distributions, and cancer risk assessment. Environmental Pollution 181, 159-166.
Zhang, R., Xie, S., Li, J., Jiang, H., Zhang, Z.-e., Liu, F., Zhao, S., Wang, Y., Yu, K., Zhang, G., 2024. Occurrence, distribution, and sources of organophosphate esters (OPEs) in the air of the Indo-China Peninsula based on a passive air monitoring network. Science of The Total Environment 929, 172762.
Zhang, Y., Lin, Y., Cai, J., Liu, Y., Hong, L., Qin, M., Zhao, Y., Ma, J., Wang, X., Zhu, T., Qiu, X., Zheng, M., 2016b. Atmospheric PAHs in North China: Spatial distribution and sources. Science of The Total Environment 565, 994-1000.
Zhang, Z., Liu, L., Li, Y.-F., Wang, D., Jia, H., Harner, T., Sverko, E., Wan, X., Xu, D., Ren, N., Ma, J., Pozo, K., 2008b. Analysis of polychlorinated biphenyls in concurrently sampled Chinese air and surface soil. Environmental Science & Technology 42, 6514-6518.
Zhao, M., Wu, J., Figueiredo, D.M., Zhang, Y., Zou, Z., Cao, Y., Li, J., Chen, X., Shi, S., Wei, Z., Li, J., Zhang, H., Zhao, E., Geissen, V., Ritsema, C.J., Liu, X., Han, J., Wang, K., 2023. Spatial-temporal distribution and potential risk of pesticides in ambient air in the North China plain. Environment International 182, 108342.
Zhao, S., Jones, K.C., Li, J., Sweetman, A.J., Liu, X., Xu, Y., Wang, Y., Lin, T., Mao, S., Li, K., 2019. Evidence for major contributions of unintentionally produced PCBs in the air of China: Implications for the national source inventory. Environmental Science & Technology 54, 2163-2171.
Zhao, S., Jones, K.C., Li, J., Sweetman, A.J., Liu, X., Xu, Y., Wang, Y., Lin, T., Mao, S., Li, K., Tang, J., Zhang, G., 2020. Evidence for major contributions of unintentionally produced PCBs in the air of China: Implications for the national source inventory. Environmental Science & Technology 54, 2163-2171.
Zhao, Y., Ma, J., Qiu, X., Lin, Y., Yang, Q., Zhu, T., 2013. Gridded field observations of polybrominated diphenyl ethers and decabromodiphenyl ethane in the atmosphere of North China. Environmental Science & Technology 47, 8123-8129.
Zhou, L., Dong, L., Huang, Y., Shi, S., Zhang, L., Zhang, X., Yang, W., Li, L., 2014. Spatial distribution and source apportionment of polycyclic aromatic hydrocarbons (PAHs) in camphor (cinnamomum camphora) tree bark from southern jiangsu, China. Chemosphere 107, 297-303.
Zhou, L., Dong, L., Huang, Y.R., Shi, S.X., Zhang, L.F., Zhang, X.L., Yang, W.L., 2015. Tree bark as a biomonitor for the determination of polychlorinated biphenyls and polybrominated diphenyl ethers from southern jiangsu, China: Levels, distribution, and possible sources. Environmental Monitoring and Assessment 187, 603.

Zhu, Y.-J., Sun, D., Yang, N.-E., Ding, Y.-S., Feng, W.-B., Hong, W.-J., Zhu, S.-M., Li, Y.-F., 2017. Temporal and spatial distributions of PBDEs in atmosphere at Shanghai rural regions, China. Environmental Science and Pollution Research 24, 10885-10892.
